# Acupuncture for tension-type headache: a systematic review and meta-analysis of randomized controlled trials

**DOI:** 10.3389/fneur.2022.943495

**Published:** 2023-05-10

**Authors:** Wen-lin Kang, Xian-jun Xiao, Rong Fan, Dong-ling Zhong, Yu-xi Li, Jian She, Juan Li, Yue Feng, Rong-jiang Jin

**Affiliations:** ^1^School of Health Preservation and Rehabilitation, Chengdu University of Traditional Chinese Medicine, Chengdu, Sichuan, China; ^2^Department of Rehabilitation Medicine, Nanbu County People's Hospital, Nanbu, Sichuan, China; ^3^School of Acupuncture-Moxibustion and Tuina/The Third Affiliated Hospital, Chengdu University of Traditional Chinese Medicine, Chengdu, Sichuan, China

**Keywords:** acupuncture, safety, tension-type headache, systematic review, meta-analysis, clinical effects

## Abstract

**Background:**

Tension-type headache (TTH) is the most common neurologic disease worldwide. Acupuncture is commonly applied to treat TTH, but evidence of acupuncture for TTH is contradictory based on previous meta-analyses. Therefore, we conducted this systematic review and meta-analysis to update the evidence of acupuncture for TTH and aimed to provide a valuable reference for clinical application.

**Methods:**

We searched 9 electronic databases from their inceptions to July 1, 2022 for randomized controlled trials (RCTs) of acupuncture for TTH. We also manually searched reference lists and relevant websites, and the experts in this field were consulted for possible eligible studies. Two independent reviewers conducted literature screening, data extraction, and risk of bias assessment. The revised Cochrane risk-of-bias tool (ROB 2) was used to assess the risk of bias of included studies. Subgroup analyses were carried out based on frequency of acupuncture, total sessions, treatment duration, needle retention, types of acupuncture and categories of medication. Data synthesis was performed using Review Manager 5.3 and Stata 16. The Grading of Recommendations Assessment, Development and Evaluation Approach (GRADE) was used to evaluate the certainty of evidence of each outcome. Meanwhile, the Standards for Reporting Interventions in Clinical Trials of Acupuncture (STRICTA) was used to assess the reporting quality of interventions in clinical trials of acupuncture.

**Results:**

30 RCTs involving 2,742 participants were included. According to ROB 2, 4 studies were considered as low risk, and the rest studies were some concerns. After treatment, compared with sham acupuncture, acupuncture had greater effect in improvement of responder rate [3 RCTs, RR = 1.30, 95%CI (1.13, 1.50), *I*^2^ = 2%, moderate certainty] and headache frequency [5 RCTs, SMD = −0.85, 95%CI (−1.58, −0.12), *I*^2^ = 94%, very low certainty]. In contrast to medication, acupuncture was more effective to reduce pain intensity [9 RCTs, SMD = −0.62, 95%CI (−0.86, −0.38), *I*^2^ = 63%, low certainty]. Adverse events were evaluated in 16 trials, and no serious event associated with acupuncture occurred.

**Conclusions:**

Acupuncture may be an effective and safe treatment for TTH patients. Due to low or very low certainty of evidence and high heterogeneity, more rigorous RCTs are needed to verify the effect and safety of acupuncture in the management of TTH.

## 1. Introduction

Tension-type headache (TTH) is manifested by bilateral compression or crunching pain in head, which is usually accompanied by photophobia or phonophobia (1), and is more prevalent in women than in men. The International Headache Society (IHS) claimed that TTH was the most common neurological disease in the world, with a reported incidence of 30–78% in the general population (2). According to data from the Global Burden of Disease (GBD), 2.33 billion individuals worldwide had TTH in 2017 (3). Of note, over the past 10 years, the global prevalence of TTH increased at a rate of 15.3% (4). Additionally, anxiety, depression and sleep issues were prevalent in patients with TTH (5–7). About 60% of TTH patients reported diminished social and occupational function (8). According to a cross sectional epidemiological survey from Danish, the absence days from work due to headache were estimated to be 820 days per 1,000 TTH patients a year (9), which bring a significant financial burden to patients and society (10).

According to the latest diagnostic criteria (2), TTH could be divided into three subtypes based on headache frequency: episodic (<1 headache day/month), frequent (1–14 headache days/month), and chronic (≥15 headache days/month). Episodic TTH can be controlled with acute medication and lifestyle modification, while frequent or chronic TTH may require special interventions, such as pharmacotherapy, acupuncture, exercise, stress reduction, etc. Antidepressants or non-steroidal anti-inflammatory medicines (NSAIDs) are main medications to treat TTH (11, 12). Currently, amitriptyline is the most widely used prophylactic medication for TTH (13). It is reported that the common adverse effects of amitriptyline are urine retention, constipation, agitation, cognitive dysfunction, etc. (14, 15). Thus, undesirable adverse events and low adherence rate of medication may be associated with poor clinical outcome (16). Furthermore, the studies revealed that TTH patients who experienced frequent headache, were more prone to take medication in excess, and increased the risk of developing medication overuse headache (17, 18). As a consequence, non-pharmacological therapy is important for TTH sufferers (17).

As an alternative medicine treatment, acupuncture is commonly used for headache sufferers with better clinical efficacy and less side effects (19, 20). Endres et al. found that acupuncture was superior to sham acupuncture in increasing responder rate for TTH patients (21). Melchart and his colleagues concluded that manual acupuncture was better than no treatment in reducing headache frequency of TTH (22). Zheng et al. reported that 8-week acupuncture treatment was effective to alleviate pain intensity in patients with chronic TTH (23). However, previous systematic reviews and meta-analyses of acupuncture for TTH hold inconsistent results (20, 24–26). In addition, several randomized controlled trials **(**RCTs) have been carried out in recent years (23, 27–36). Therefore, we conducted this systematic review and meta-analysis to update the evidence of the effect and safety of acupuncture for TTH.

## 2. Methods

This systematic review and meta-analysis was conducted in accordance with A Measurement Tool to Assess Systematic Reviews (AMSTAR 2) (37) and reported according to the Preferred Reporting Items for Systematic Reviews and Meta-Analyses (PRISMA) guidelines (38). The protocol of this systematic review and meta-analysis was registered in the INPLASY (https://inplasy.com/inplasy-2022-3-0047/).

### 2.1. Inclusion criteria

We employed the following inclusion criteria when selecting studies: (1) Participants: adults who were diagnosed with TTH (diagnostic criteria released by the International Headache Society) (2, 39–41); (2) Intervention: acupuncture (manual acupuncture/electro-acupuncture) (42); (3) Control: sham acupuncture, medication, exercise, and other controls (such as waiting list, usual care, etc.); (4) Outcomes: primary outcome was responder rate, and secondary outcomes included headache frequency, pain intensity, headache duration, consumption of medication, other relevant outcomes and acupuncture related adverse events; (5) Study design: RCTs, or cross-over RCTs which investigated the effect and safety of acupuncture for TTH.

### 2.2. Exclusion criteria

We adopted the following exclusion criteria: (1) Full text or data could not be obtained through useful approaches; (2) Acupuncture combined with traditional Chinese medicine therapy (other types of acupuncture, moxibustion, herbal medicine, etc.); (3) No details of diagnostic criteria, acupuncture treatment or control intervention were provided; (4) Overlapping publications.

### 2.3. Literature search

We searched 5 English databases (PubMed, Web of Science, Embase, the Cochrane Library and Epistemonikos) and 4 Chinese databases (China National Knowledge Infrastructure, Wanfang Database, Chinese Science and Technology Periodical Database and China Biology Medicine) from their inceptions to July 1, 2022. To retrieve additional trials, we manually searched reference lists of included articles and relevant reviews. The gray literature including dissertations and conference proceedings was also examined. In addition, we searched clinical registries (e.g., Chinese Clinical Trial Registry, Clinical Tials.gov), and the experts in this field were consulted for possible eligible studies. The search strategies of the above databases are shown in Appendix 1.

### 2.4. Outcome measurement

#### 2.4.1. Primary outcome

Responder rate (43): at least 50% reduction of headache days.

#### 2.4.2. Secondary outcomes

Headache frequency: number of headache days per defined period.

Pain intensity: (1) Visual Analog Scale (VAS); (2) Von Korff (questions 1–3) pain intensity score; (3) German version of the pain disability index; (4) Numerical Rating Scale (NRS); (5) Verbal Rating Scale (VRS).

Headache duration: hours with headache per defined period.

Consumption of medication: sum of analgesics taken per month.

Other related outcomes: depression and anxiety level assessed with valid and reliable scales.

Acupuncture-related adverse events: subcutaneous hematoma, pain, acupuncture syncope reaction, etc.

### 2.5. Literature screening

ENDNOTE X9 was used to manage the retrieved records. After removing duplicates, two reviewers (WLK & XJX) independently scrutinized the titles and abstracts for potential eligible literature. Then, two reviewers (RF & JS) independently screened the full text according to inclusion and exclusion criteria. After cross-checking, disagreements were settled through consultation with an experienced reviewer (JL).

### 2.6. Data extraction

Two reviewers (RF & JS) extracted data using a pre-designed extraction form. The following data were extracted: (1) Study information (e.g., first author, year, country, etc.); (2) Participant characteristics (e.g., gender, age, etc.); (3) Details of intervention and control group (e.g., duration, types of acupuncture, etc.); (4) Results of each outcome; (5) Information related to the risk of bias. In case of missing data, we contacted the corresponding authors for necessary data. As for overlapping publications, the most recent report or complete report was included for data analysis. With regard to cross-over RCTs, the data before the intersection was extracted. For data expressed as mean and standard error, mean and 95% confidence intervals (CI), or median and interquartile range, we converted these data into mean and standard deviation. If the data was displayed in the graph, the GetData Graph Digitizer 2.26 was used to extract the data. After extraction, two reviewers (RF & JS) cross-checked the extracted data. Any inconsistency during the process of data extraction was resolved through discussions with an experienced reviewer (DLZ).

### 2.7. Risk of bias assessment

Two researchers (YXL & DLZ) independently used the version 2 of the Cochrane tool for assessing risk of bias in randomized trials (ROB 2) (44) to appraise the risk of bias of the included RCTs. The ROB 2 considers bias from 5 different domains: randomization process, deviations from intended interventions, missing outcome data, measurement of the outcome, and selection of the reported results. The risk of bias in each domain and overall are categorized into “low risk of bias”, “some concerns”, or “high risk of bias”. In the case of disagreements, a third reviewer (JL) was involved.

### 2.8. Evaluation of the reporting quality of interventions in clinical trials of acupuncture

The Revised Standards for Reporting Interventions in Clinical Trials of Acupuncture (STRICTA) (45) is designed to assess the reporting quality of interventions in clinical trials of acupuncture. The revised STRICTA checklist comprises six items, including the acupuncture rationale, the details of needling, the treatment regimen, other components of treatment, the practitioner's background, and the control or comparator interventions. Two reviewers (DLZ & YXL) independently evaluated the included RCTs with the revised STRICTA checklist, and any disagreement was arbitrated by consultation with a third reviewer (JL).

### 2.9. Certainty of evidence assessment

The certainty of the evidence was evaluated by two independent reviewers (WLK & RF) using the Grading of Recommendation, Assessment, Development, and Evaluation (GRADE) system (46). Additionally, the “Summary of findings” table was constructed to present the certainty of each outcome with GRADE pro V 3.6 software.

### 2.10. Data analysis

Since responder rate was dichotomous data, risk ratio (RR) was used for data synthesis. Due to the different scoring standards of outcomes, such as headache frequency, pain intensity, headache duration, anxiety, depression and medication consumption, standardized mean difference (SMD) was calculated. The uncertainty was expressed with 95% confidence intervals (CI), and *P* < 0.05 was considered significant. Chi-square test and *I*^2^ statistic were used to test the statistical heterogeneity of included studies. We utilized a random-effect model (REM) to aggregate studies when *I*^2^ > 50% and *P* > 0.05, and a fixed-effect model (FEM) to merge studies in case of *I*^2^ ≤ 50% and *P* ≤ 0.05. We conducted subgroup analyses based on: (1) frequency of acupuncture, (2) total sessions, (3) treatment duration, (4) needle retention, (5) types of acupuncture, and (6) categories of medication. We carried out sensitivity analysis to verify the robustness of the results by excluding the literature one by one. Publication bias of the primary outcome was assessed by funnel plot, *Begg's* and *Egger's* test when ≥10 studies of the same comparison were synthesized. Statistical analyses were performed with Review Manager 5.3 and Stata 16.

## 3. Results

**Figure 1:**
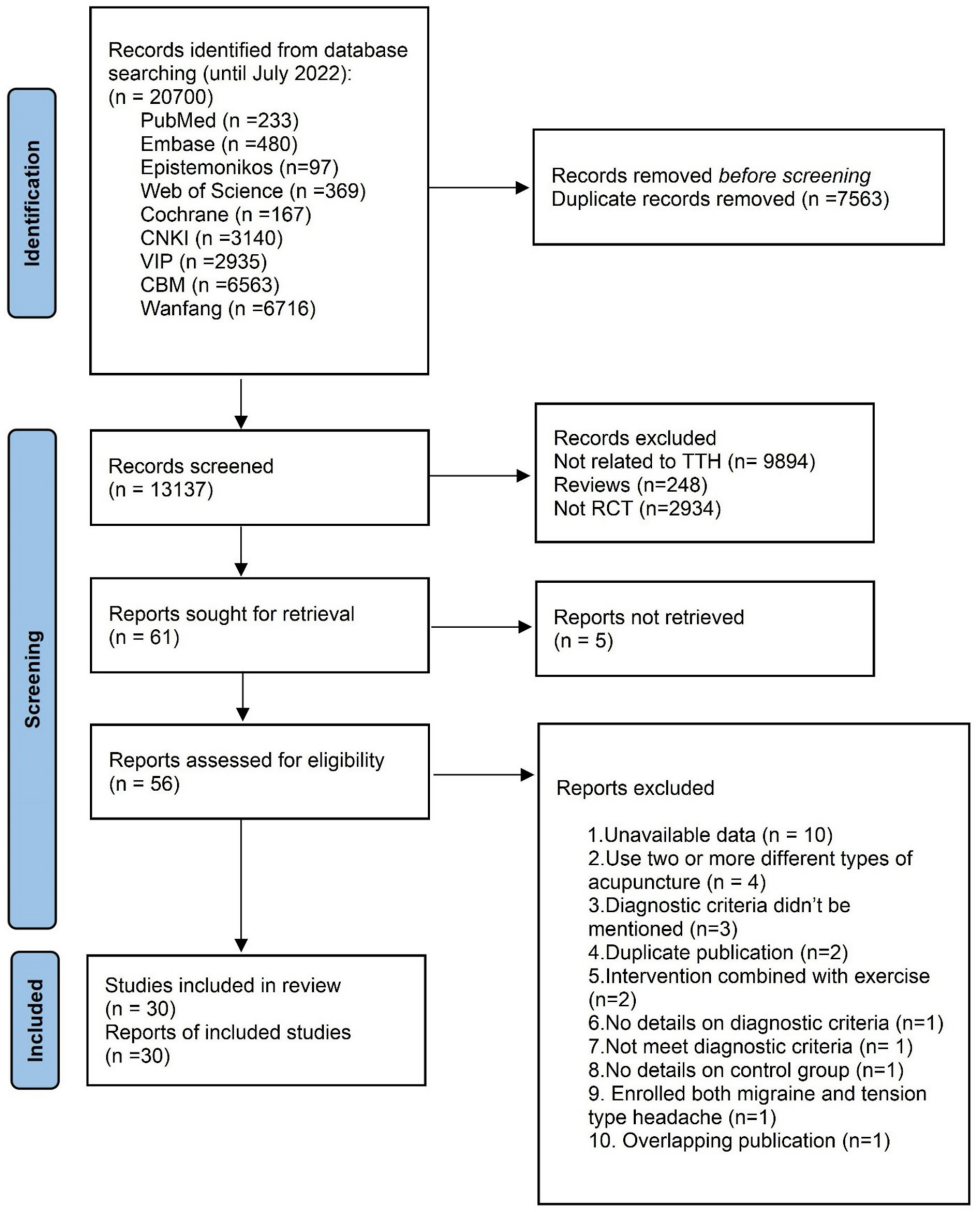
PRISMA flow chart.

We retrieved 20,700 articles from 9 databases and relevant websites. After excluding 7,563 duplicates and 13,076 irrelevant records by screening the titles and abstracts, and 5 RCTs were not retrieved, 56 articles remained for further assessment. Through reading full texts, 26 studies were excluded, and the reasons for exclusion are listed in Appendix 2. We included an article (47) as a supplement to Zhang (34), a total of 30 RCTs (21–23, 27–36, 48–64) involving 2,742 participants were included (1,349 in the intervention group and 1,393 in the control group) (Figure 1).

### 3.1. Study characteristics

The details of the included studies are shown in Table 1. Sample size ranged from 9 (61) to 409 (21). The average age of patients varied from 31 to 58 years old. The trials were conducted in 7 countries [China (23, 27, 28, 30–32, 34–36, 51–56, 62, 63) (*n* = 17), Germany (21, 22, 33, 58) (*n* = 4), Korea (29, 59) (*n* = 2), Sweden (48, 60) (*n* = 2), England (49, 61) (*n* = 2), Brazil (57) (*n* = 1), Australia (50) (*n* = 1), and Turkey (64) (*n* = 1)]. Among the included studies, two were cross-over RCTs (50, 57).

**Table 1:** The details of the included studies.

**References**	**Country**	**Diagnostic criterion**	**Sample size**	**Gender (male/ female)**	**Mean age (year)**	**Intervention**	**Frequency and sessions of acupuncture**	**Needle retention**	**Treatment duration**	**Comparison**	**Outcome**	**Follow-up**
Chassot et al. (57)	Brazil	ICHD-3	EA:18 SA:16	EA: 0/18 SA: 0/16	EA: 39.11 ± 10.5 SA: 41.44 ± 10.5	EA	Twice a week, 10 sessions	30 min	5 weeks	SA	3, 8	NR
Duan (62)	China	ICHD-2	MA:48 M:48	MA: 14/34 M: 17/31	MA: 42.7 ± 11.5 M: 43.5 ± 11.2	MA	Once a day, 21 sessions	40 min	3 weeks	Eperisone Hydrochloride 50mg, tid	3	NR
Deng (63)	China	ICHD-2	EA:25 M:25	EA: 8/17 M: 6/19	EA:32.18 ± 11.56 M: 31.62 ± 10.07	EA	Once a day, 10 sessions	30 min	10 days	Acetaminophen 0.5g, qid + Amitriptyline 25mg, qn	2,3	NR
Endres et al. (21)	Germany	ICHD-2	MA:208 M:195	MA: 46/163 M: 42/158	MA: 39.2 ± 11.4 M: 38.9 ± 12.2	MA	Twice a week, 10 sessions	30 min	6 weeks	SA	1,2,3,8	6 weeks, 18 weeks
Guo (31)	China	ICHD-2	MA:50 M:50	MA: 16/34 M: 19/31	MA: 33.2 ± 10.2 M: 34.0 ± 10.6	MA	Once every other day, 14 sessions	30 min	4 weeks	Eperisone Hydrochloride 50 mg, tid; + Flunarizine hydrochloride 5mg, qd	3	NR
Guo (28)	China	ICHD-2	MA:32 M:32	MA: 25/70 M: 26/68	MA: 42.5 ± 7.13 M: 41.77 ± 8.42	MA	Once every other day, 14 sessions	30 min	4 weeks	Deanxit bid	6, 7	4 weeks
Huang (51)	China	ICHD-3	MA:34 M:21	MA: 16/17 M: 8/9	MA:49.39 ±7.49 M:51.94 ± 7.79	MA	Once every other day, 14 sessions	30 min	4 weeks	Acetaminophen 0.3 g, qd or bid	8	NR
Jeon and Lee (29)	Korea	ICHD-3	EA:15 SA:15	EA: 6/9 SA: 8/7	EA:40.00 ±13.11 SA:34.33 ± 11.48	EA	3 times a week, 3 sessions	20 min	1 week	SA	2,3	1 week
Karst et al. (58)	Germany	IHS1988	MA:34 SA:35	MA: 17/17 SA: 14/21	MA: 47.9 ±13.8 SA: 48.2 ±14.6	MA	Twice a week, 10 sessions	30 min	5 weeks	SA	2,3,5	6 weeks, 20 weeks
Kwak et al. (59)	Korea	ICHD-2	MA:17 SA:15	MA: 3/14 SA: 3/12	MA:45.05 ± 12.57 SA: 49.4 ±11.14	MA	Twice a week, 8 sessions	25 min	4 weeks	SA	3,8	4 weeks, 8 weeks, 12 weeks
Koran et al. (64)	Turkey	ICHD-3	MA:40 SA:41 M:48	MA: 18/22 SA: 18/23 M: 22/26	NR	MA	2–3 times a week, 8 sessions	20 min	4 weeks	1.SA 2. Antidepressant + analgesic	3	1 week, 12 weeks
Liu (36)	China	ICHD-3	MA:32 M:30	MA: 13/19 M: 12/18	MA:50.53 ± 13.70 M: 48.33 ± 13.17	MA	5 times a week, 20 sessions	30 min	4 weeks	Escitalopram oxalate 10 mg, qd	7,8	4 weeks
Melchart et al. (22)	Germany	ICH-10	MA:132 SA:63 WT:75	MA: 72/95 SA: 73/46 WT:77/58	MA:42.3 ±13.5 SA:43.4 ± 12.9 WT:42.8 ± 13.2	MA	1–2 times a week, 12 sessions	30 min	8 weeks	1.SA 2.WT	1,2,3 4,7,8	4 weeks, 16 weeks
Nie (30)	China	ICHD-3	MA:41 M:41	MA: 12/29 M: 18/23	MA:50.02 ± 10.66 M: 46.54 ±11.46	MA	3 times a week, 12 sessions	20 min	4 weeks	Eperisone Hydrochloride 50 mg, tid	2,3,8	4 weeks, 8 weeks
Söderberg et al. (60)	Sweden	IHS1988	MA:30 E:30 RT:30	MA: 7/23 E: 7/23 RT: 3/27	MA: 35 ± 10.25 E: 35 ± 9.5 RT: 43.5 ± 9.25	MA	Once a week, 10–12 sessions	30 min	10–12 weeks	1.E 2.RT	3	12 weeks, 24 weeks
Schiller et al. (33)	Germany	ICHD-3	MA:24 UC:24 E:24 MA+E:24	MA: 6/18 UC:7/17 E: 5/18 MA+E: 2/22	MA: 39.8 ±12.2 UC: 38.7 ±14.6 E: 37 ±15.3 MA+E: 39 ± 11.6	MA	1–3 times a week, 12 sessions	30 min	6 weeks	1.UC 2.E 3. MA+E	1,3,8	6 weeks, 18 weeks
Tavola et al. (48)	Sweden	IHS 1988	MA:15 SA:15	MA: 2/13 SA: 2/13	MA: 32.5 ± 10 SA: 33.3 ± 13.3	MA	Once a week, 8 sessions	20 min	8 weeks	SA	5	4 weeks, 16 weeks, 40 weeks
White et al. (49)	England	IHS 1988	MA:25 SA:25	MA: 7/18 SA: 5/20	MA: 49.8 ± 2.9 SA: 48.2 ± 2.9	MA	Once a week, 6 sessions	Without needle retention	6 weeks	SA	1,2,3,4	4 weeks, 8 weeks
Wu (52)	China	ICHD-2	MA:30 M:30	MA: 18/12 M: 15/15	MA: 44.3 ± 12.49 M: 42.87 ± 9.38	MA	5 times a week, 10 sessions	30 min	2 weeks	Eperisone Hydrochloride 50 mg, tid	6,7,8	NR
Wang (27)	China	ICHD-2	MA:29 M:27	MA: 8/21 M: 7/20	MA: 38 ± 10 M: 39 ± 11	MA	3 times a week, 18 sessions	30–45 min	48 days	Eperisone Hydrochloride 50 mg, tid	3,4	NR
White et al. (61)	England	IHS 1988	MA:4 SA:5	MA: 1/3 SA: 1/4	MA: 57.2 ±13.6 SA: 57.4 ±19.9	MA	Once a week, 6 sessions	Without needle retention	6 weeks	SA	8	NR
Wang (35)	China	ICHD-3	MA:150 M:100	MA: 63/87 M: 42/58	MA: 44 ± 13 M: 43 ±13	MA	3 times a week, 9 sessions	4–6 hours	3 weeks	Eperisone Hydrochloride 50mg, tid	3	NR
Xiang (53)	China	ICHD-2	MA:30 M:30	MA: 13/17 M: 10/20	MA: 40.7 ± 10.6 M: 40.43 ± 12.92	MA	6 times a week, 24 sessions	30 min	4 weeks	Amitriptyline hydrochloride 25mg, tid	3	NR
Xue et al. (50)	Australia	IHS 1988	EA:20 SA:20	EA: 7/13 SA: 7/13	EA: 42.6 ±1.8 SA: 41.5 ±1.9	EA	Twice a week, 8 sessions	30 min	4 weeks	SA	2,3,8	NR
Yang (32)	China	ICHD-2	MA:41 M:41	MA: 46/163 M: 42/158	MA: 40.96 ± 8.23 M: 41.29 ± 8.32	MA	Once a day, 14 sessions	30 min	2 weeks	Ibuprofen 0.3 g, bid	3	NR
Zheng et al. (23)	China	ICHD-3	MA:110 SA:108	MA: 28/82 SA: 33/75	MA: 43 ± 12.5 SA: 43.2 ± 12.8	MA	2–3 times a week, 20 sessions	30 min	8 weeks	SA	1,2,3,8	4, 8, 12, 16, 20, 24 weeks
Zhou (55)	China	ICHD-2	MA:30 M:30	MA: 12/18 M: 14/16	MA: 44.77 ±12.42 M: 45.93 ±12.30	MA	5 times a week, 20 sessions	30 min	4 weeks	Amitriptyline hydrochloride 25 mg, qd	6,7	NR
Zhang (54)	China	ICHD-2	MA:26 SA+M:20	NR	NR	MA	3 times a week, 12 sessions	30 min	4 weeks	SA+ Estazolam 0.5 mg, qn	1,2,4,8	12 weeks
Zhang (34, 47)	China	ICHD-3	MA:29 M:30	MA: 13/16 M: 13/17	MA: 31.79 ± 8.56 M: 32.03 ± 6.49	MA	Once a day, 28 sessions	30 min	4 weeks	Amitriptyline	3,6,7	NR
Zhu (51)	China	ICHD-2	MA:30 M:30	MA: 9/21 M: 7/23	MA: 39.17 ± 10.15 M: 40.07 ± 9.82	MA	6 times a week, 24 sessions	30 min	4 weeks	Amitriptyline 25 mg, bid; + Oryzanol 30 mg, tid	8	4 weeks

1. Responder rate (at least 50% reduction of headache days); 2. Headache frequency; 3. Pain intensity; 4. Headache duration; 5. Consumption of medication; 6. Anxiety; 7. Depression; 8. Adverse event. MA, manual acupuncture; EA, electro-acupuncture; SA, sham acupuncture; RT, relaxation training; WT, waiting list; UC, usual care; M, medication; E, exercise; NR, no report; IHS, International Headache Society; ICHD, International Classification of Headache Disorders; min, minutes.

For acupuncture treatment, 4 RCTs applied electro-acupuncture (29, 50, 57, 63), and the rest 26 studies used manual acupuncture. With regard to the needle retention time, 2 studies applied no needle retention (49, 61), 1 RCT retained for 4–6 hours (35), the remaining RCTs retained needles from 20 to 45 minutes. The frequency of acupuncture treatment was usually 2 or 3 times per week. The treatment duration of acupuncture ranged from 1 week (29) to 12 weeks (60). A total of 16 RCTs (21–23, 28–30, 33, 36, 48, 49, 54, 56, 58–60, 64) observed the effect of follow-up, the follow-up period was from 1 week (29) to 40 weeks (48). Among included studies, 1 study was four-arm trial (33), 3 RCTs were three-arm (22, 60, 64), the rest studies were two-arm. With regard to comparison, 16 studies applied medication [*antidepressant* (28, 34, 36, 53, 55, 56), *muscle relaxant* (27, 30, 31, 35, 52, 62), *analgesics* (32, 51), *antidepressant plus analgesics* (63, 64)], 12 studies used sham acupuncture (21–23, 29, 48–50, 57–59, 61, 64), 2 applied exercise (33, 60), 1 utilized relaxation training (60), 1 applied usual care (33), 1 was waiting list (22), 1 was acupuncture plus exercise (33), and 1 adopted sham acupuncture plus medication (54). The most commonly used acupoints were Baihui (GV20), Taiyang (EX-HN5), Fengchi (GB20), Hegu (LI4), Yintang (GV29), Taichong (LR3), Neiguan (PC6), Zusanli (ST36), and “Ashi” points (Appendix 3).

### 3.2. The reporting quality of interventions in clinical trials of acupuncture

The STRICTA checklist is shown in Appendix 4. All studies reported the acupoint selection, needle retention time, total sessions of treatment, frequency and duration. Five studies (49, 57, 58, 60, 61) did not describe the style of acupuncture. 17 trials (21–23, 28, 31, 33, 35, 36, 48–53, 60, 63, 64) specified the reasoning of treatment. Patients in 10 RCTs (23, 27, 29, 32, 34, 36, 52, 53, 57, 62) were treated with fixed acupoint protocols, 14 studies (21, 22, 28, 31, 33, 49, 54–56, 58, 60, 61, 63, 64) used a fixed set of acupoints combined with acupoints based on syndrome differentiation, and 5 RCTs (30, 35, 48, 50, 59) applied individualized acupoint protocols. A total of 15 studies (21–23, 27, 29, 31, 33, 36, 48, 52, 57, 58, 60, 61, 64) mentioned the number of needle insertions. More than half of the studies (21, 23, 27, 30–36, 48, 51–53, 55, 60, 63, 64) described the depth of insertion. De qi sensation or other response sought were required in 21 RCTs (21–23, 27, 30, 33, 36, 48–56, 60–64). Except for 5 trials (21–23, 33, 64), the rest studies did not specify the setting and context of treatment. Among included studies, 9 RCTs (21–23, 33, 49, 57, 60, 61, 64) provided information about the acupuncturist's background. Six studies (21–23, 33, 55, 64) reported details of other interventions administered to the acupuncture group. All studies described the control group in detail, and 13 RCTs (21–23, 33, 48–50, 57–61, 64) elucidated the rationale of control group.

### 3.3. Risk of bias of included studies

**Figure 2:**
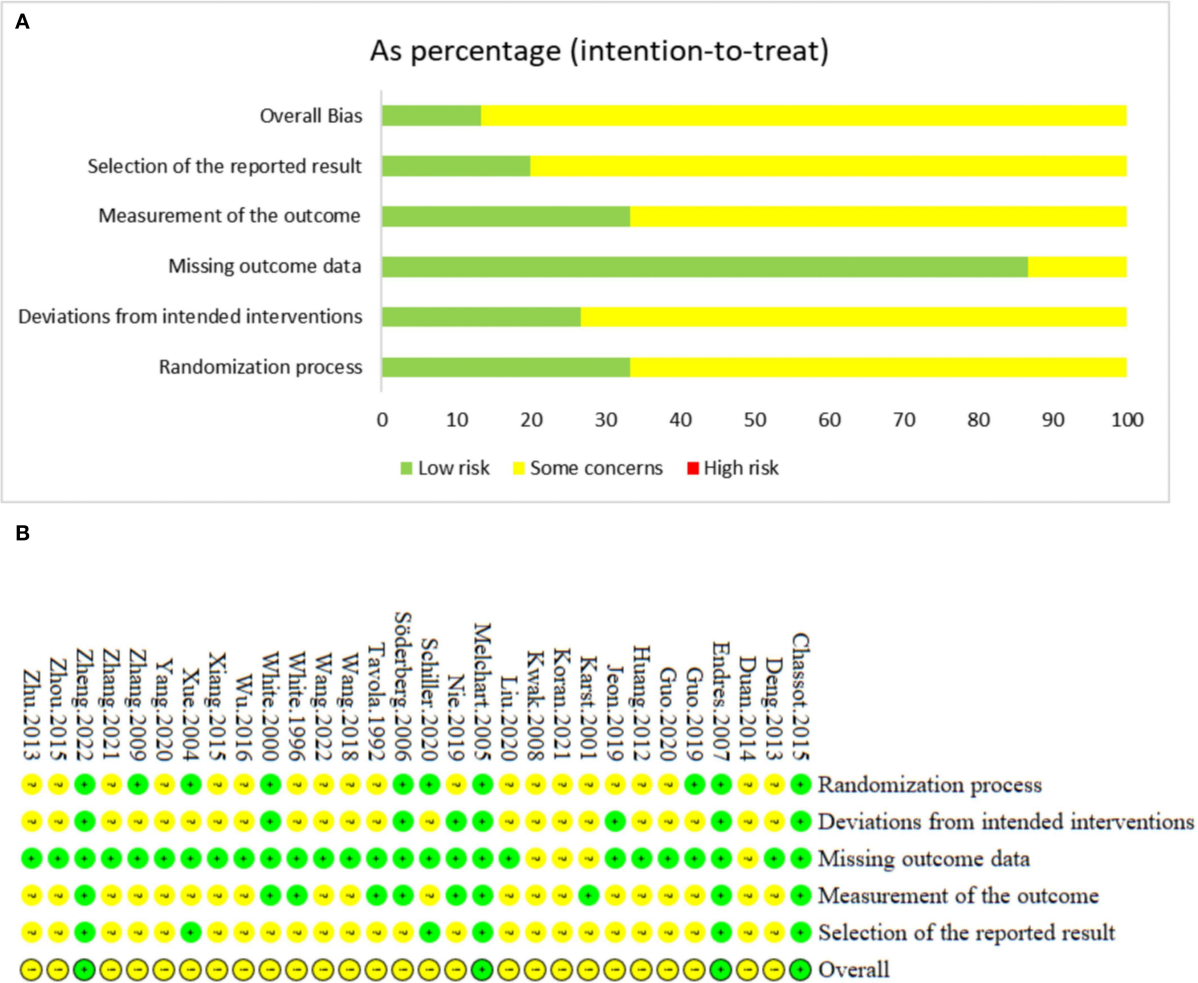
**(A)** The graph of risk of bias. **(B)** The summary of risk of bias.

The results of risk of bias are shown in Figure 2. In the randomization process, a total of 10 studies (21–23, 28, 33, 49, 50, 54, 57, 60) were judged as low risk, while the rest 20 studies were assessed as some concerns because of neglecting the allocation concealment. As for deviation from intended interventions, 8 trials (21–23, 30, 33, 49, 57, 60) reported that no deviations from the intended intervention were related to experimental context and intention-to-treat (ITT) analysis was applied, thus they were considered as low risk. The remaining 22 RCTs were judged as some concerns due to no double-blinding and lacking of ITT analysis. For the missing outcome, 4 RCTs (58, 59, 62, 64) did not provided details of dropped-outs, which were assessed as some concerns. The rest 26 RCTs were judged as low risk. Considering the measurement of outcomes, 10 studies were rated as low risk (21–23, 30, 48, 49, 57, 58, 60, 61). The rest 20 RCTs were judged as some concerns for lacking of blinding method of outcome assessors. With regard to the selection of the reported result, 6 trials (21–23, 33, 50, 57) provided protocol information and reported all the expected outcomes, thus were considered as low risk. The rest 24 RCTs were some concerns. In summary, the overall bias of 4 RCTs were judged as low risk, and the rest 26 trials were some concerns (Appendix 5).

### 3.4. Effects of acupuncture for TTH

#### 3.4.1. Acupuncture vs. sham acupuncture

##### 3.4.1.1. Responder rate

**Figure 3:**
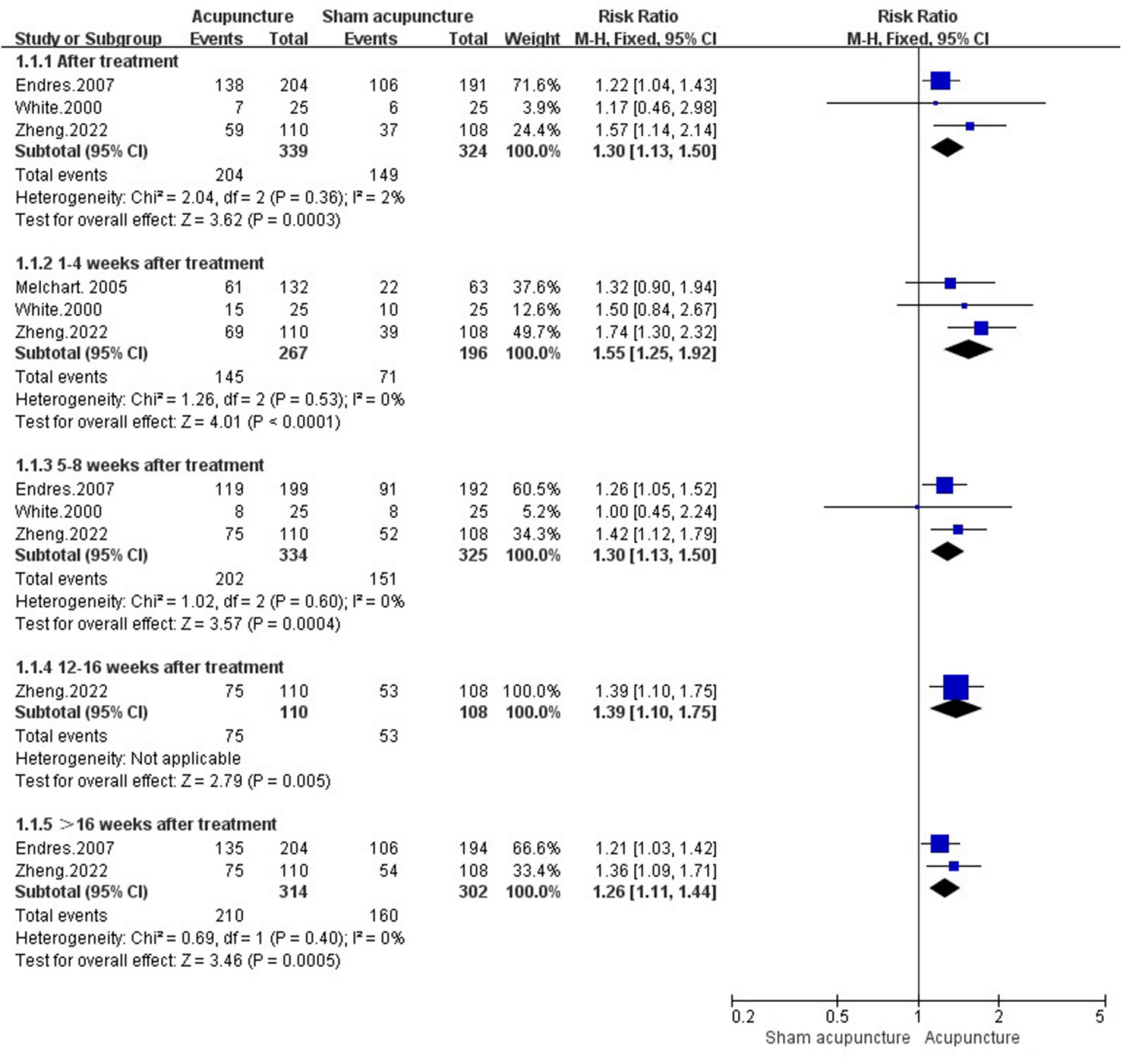
Responder rate in comparison of acupuncture vs. sham acupuncture.

Four studies (I: 471 participants, C: 387 participants) (21–23, 49) reported the responder rate. After treatment, the responder rate in acupuncture group was higher than sham acupuncture group [RR = 1.30, 95%CI (1.13, 1.50), *P* = 0.0003, *I*^2^ = 2%]. During the follow-up, acupuncture had long-term therapeutic effect in improving responder rate [1–4 weeks after treatment: RR = 1.55, 95%CI (1.25, 1.92), *P* < 0.0001, *I*^2^ = 0%; 5–8 weeks after treatment: RR = 1.30, 95%CI (1.13, 1.50), *P* = 0.0004, *I*^2^ = 0%; 12–16 weeks after treatment: RR = 1.39, 95%CI (1.10, 1.75), *P* = 0.005; **>**16 weeks after treatment: RR = 1.26, 95%CI (1.11, 1.44), *P* = 0.0005, *I*^2^ = 0%] (Figure 3).

##### 3.4.1.2. Headache frequency

**Figure 4:**
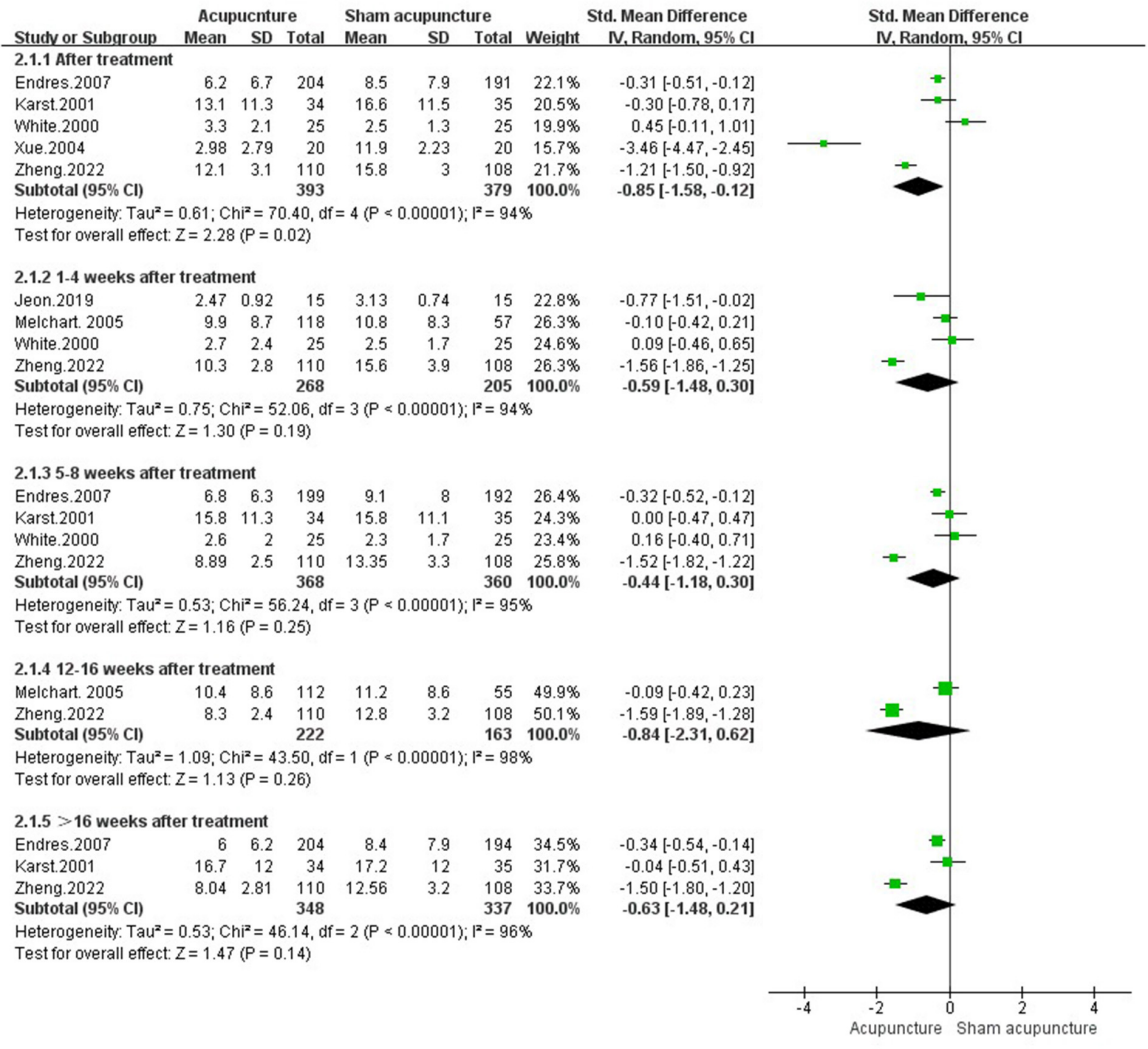
Headache frequency in comparison of acupuncture vs. sham acupuncture.

Seven studies (21–23, 29, 49, 50, 58) evaluated headache frequency in comparison of acupuncture vs. sham acupuncture. Results demonstrated that acupuncture had less frequent headache attacks [SMD = −0.85, 95%CI (−1.58, −0.12), *P* = 0.02, *I*^2^ = 94%]. While there was no long-term therapeutic effect in acupuncture group [1–4 weeks after treatment: SMD = −0.59, 95%CI (−1.48, 0.30), *P* = 0.19, *I*^2^ = 94%; 5–8 weeks after treatment: SMD = −0.44, 95%CI (−1.18, 0.30), *P* = 0.25, *I*^2^ = 95%; 12–16 weeks after treatment: SMD = −0.84, 95%CI (−2.31, 0.62), *P* = 0.26, *I*^2^ = 98%; >16 weeks after treatment: SMD = −0.63, 95%CI (−1.48, 0.21), *P* = 0.14, *I*^2^ = 96%] (Figure 4).

##### 3.4.1.3. Pain intensity

**Figure 5:**
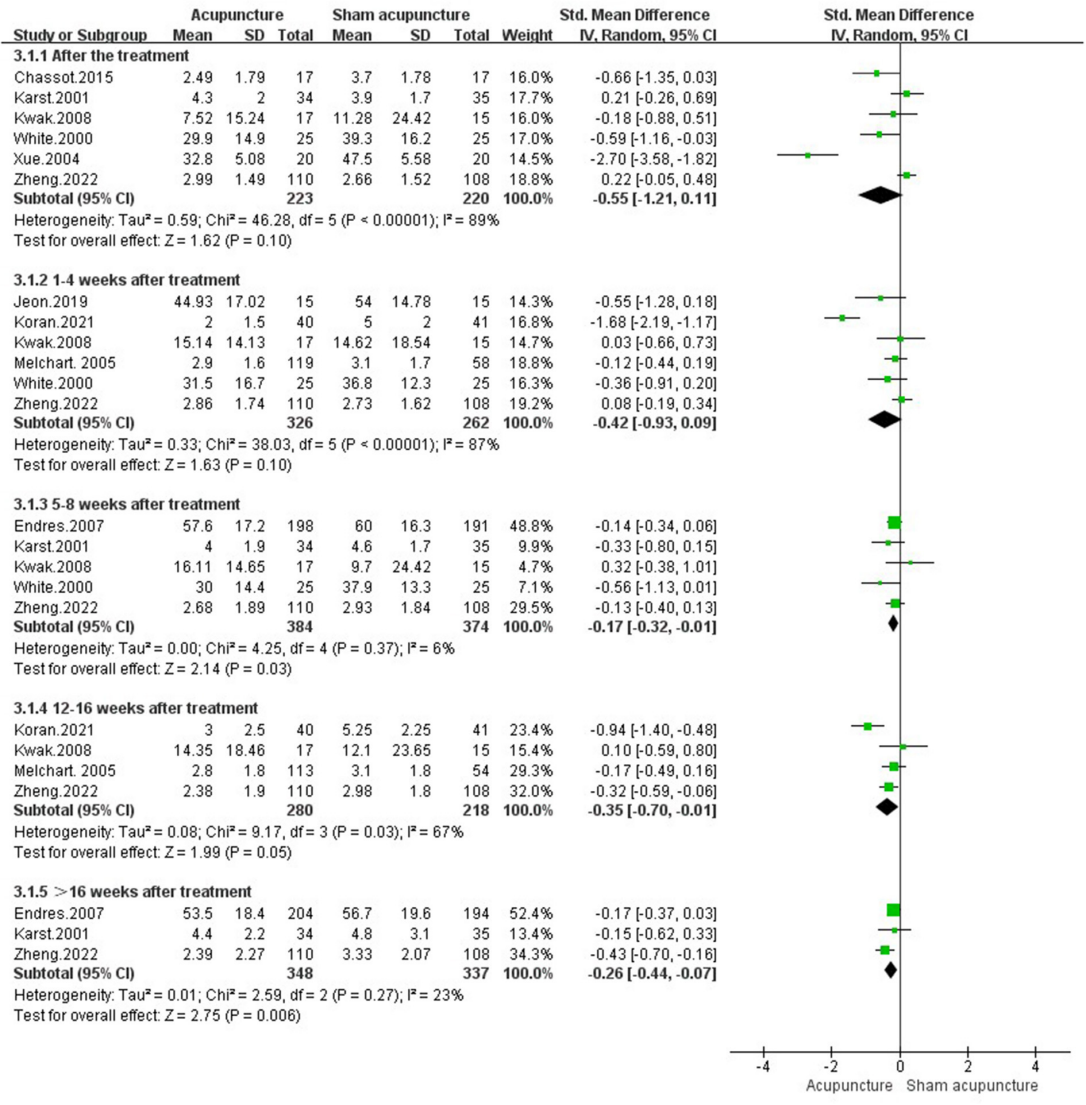
Pain intensity in comparison of acupuncture vs. sham acupuncture.

Ten studies with 1,129 participants (I: 601, C: 528) observed pain intensity (21–23, 29, 49, 50, 57–59, 64). After treatment, there was no difference between acupuncture and sham acupuncture [SMD = −0.55, 95%CI (−1.21, 0.11), *P* = 0.10, *I*^2^ = 89%]. However, 5 weeks after treatment, acupuncture showed significant effect in reducing pain intensity [5–8 weeks after treatment: SMD = −0.17, 95%CI (−0.32, −0.01), *P* = 0.03, *I*^2^ = 6%; 12–16 weeks after treatment: SMD = −0.35, 95%CI (−0.70, −0.01), *P* = 0.05, *I*^2^ = 67%;**>**16 weeks after treatment: SMD = −0.26, 95%CI (−0.44, −0.07), *P* = 0.006, *I*^2^ = 23%] (Figure 5).

##### 3.4.1.4. Headache duration

Results of 2 RCTs (I: 143 participants, C: 82 participants) (22, 49) showed that there was no difference between acupuncture and sham acupuncture in reducing headache duration whether after treatment [SMD = 0.34, 95%CI (−0.21, 0.90), *P* = 0.23] or during follow up [1–4 weeks after treatment: SMD = −0.14, 95%CI (−0.41, 0.14), *P* = 0.32, *I*^2^ = 0%; 5–8 weeks after treatment: SMD = 0.28, 95%CI (−0.28, 0.84), *P* = 0.33; 12–16 weeks after treatment: SMD = −0.15, 95%CI (−0.47, 0.18), *P* = 0.37] (Appendix Figure 1).

##### 3.4.1.5. Consumption of medication

Two studies reported consumption of analgesics after treatment (48, 58). However, no significant difference was detected between acupuncture and sham acupuncture after treatment [SMD = −1.23, 95%CI (−3.24, 0.78), *P* = 0.23, *I*^2^ = 93%]. However, the effect of acupuncture during follow-up was inconsistent [1–4 weeks after treatment: SMD = −1.90, 95%CI (−2.78, −1.02), *P* < 0.0001, *I*^2^ = 93%; 5–8 weeks after treatment: SMD = −0.39, 95%CI (−0.86, 0.09), *P* = 0.11; 12–16 weeks after treatment: SMD = −1.86, 95%CI (−2.73, −0.98), *P* < 0.0001; >16 weeks after treatment: SMD = −1.35, 95%CI (−2.15, −0.54), *P* = 0.001] (Appendix Figure 2).

##### 3.4.1.6. Depression

Two trials involving 233 adults (I:145, C:88) focused on depressive state of TTH patients (22, 58), and no difference was identified between acupuncture and sham acupuncture after treatment [SMD = −0.04, 95%CI (−0.51, 0.43), *P* = 0.87] or during follow up period [1–4 weeks after treatment: SMD = −0.16, 95%CI (−0.49, 0.17), *P* = 0.34; 5–8 weeks after treatment: SMD = −0.31, 95%CI (−0.78, 0.17), *P* = 0.20; 12–16 weeks after treatment: SMD = −0.07, 95%CI (−0.40, 0.27), *P* = 0.69] (Appendix Figure 3).

#### 3.4.2. Acupuncture vs. medication

##### 3.4.2.1. Pain intensity

**Figure 6:**
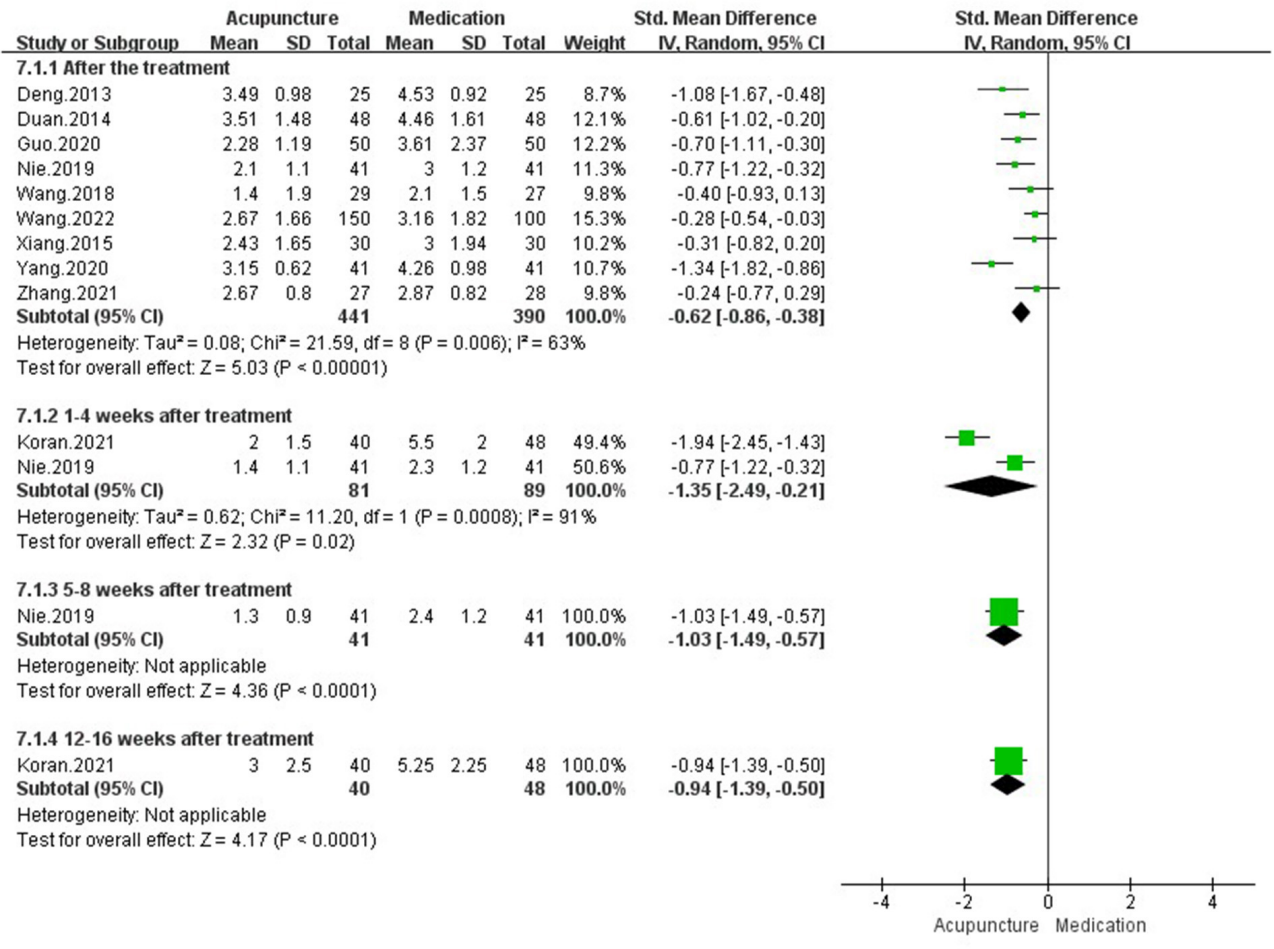
Pain intensity in comparison of acupuncture vs. medication.

After pooling data from 10 studies (27, 30–32, 34, 35, 53, 62–64) with 919 adults (I: 481, C: 438), we found acupuncture could relieve more pain intensity than medication after treatment [SMD = −0.62, 95%CI (−0.86, −0.38), *P* < 0.00001, *I*^2^ = 63%] and during follow up period [1–4 weeks after treatment: SMD = −1.35, 95%CI (−2.49, −0.21), *P* = 0.02, *I*^2^ = 91%; 5–8 weeks after treatment: SMD = −1.03, 95%CI (−1.49, −0.57), *P* < 0.0001; 12–16 weeks after treatment: SMD = −0.94, 95%CI (−1.39, −0.50), *P* < 0.0001] (Figure 6).

##### 3.4.2.2. Headache frequency

The synthesized results from 2 studies (I: 66 participants, C: 66 participants) (30, 63) showed that acupuncture was not superior to medication in reducing headache attacks after treatment [SMD = −0.60, 95%CI (−1.41, 0.20), *P* = 0.14, *I*^2^ = 79%] or during follow up [1–4 weeks after treatment: SMD = −0.20, 95%CI (−0.64, 0.23), *P* = 0.36; 5–8 weeks after treatment: SMD = −0.30, 95%CI (−0.73, 0.14), *P* = 0.18] (Appendix Figure 4).

##### 3.4.2.3. Headache duration

Wang et al. (27) reported that acupuncture could decrease more headache duration than medication after treatment (*P*<0.05).

##### 3.4.2.4. Depression

Five studies (I: 150 participants, C: 149 participants) evaluated the depressive symptoms of patients with TTH (28, 36, 47, 52, 55), and no difference was found between acupuncture and medication in relieving depression after treatment [SMD = −0.37, 95%CI (−0.90, 0.15), *P* = 0.161, *I*^2^ = 80%] or during follow up [1–4 weeks after treatment: SMD = −0.05, 95%CI (−0.65, 0.56), *P* = 0.88, *I*^2^ = 65%] (Appendix Figure 5).

##### 3.4.2.5. Anxiety

Four studies (I: 118 participants, C: 119 participants) observed anxiety in TTH patients (28, 47, 52, 55), and acupuncture was not better than medication in relieving anxiety after treatment [SMD = −0.12, 95%CI (−0.69, 0.45), *P* = 0.68, *I*^2^ = 79%] or during follow up [1–4 weeks after treatment: SMD = 0.09, 95%CI (−0.41, 0.60), *P* = 0.71] (Appendix Figure 6).

#### 3.4.3. Acupuncture vs. exercise

##### 3.4.3.1. Responder rate

Schiller et al. (33) found that acupuncture was not superior to exercise in improving responder rate (*P* > 0.05) during follow up period.

##### 3.4.3.2. Headache frequency

Schiller et al. (33) discovered that no differences were detected between acupuncture and exercise in reducing headache frequency during follow up period.

##### 3.4.3.3. Pain intensity

Two studies (33, 60) reported the pain intensity after treatment, the result showed that acupuncture did not differ from exercise in ameliorating pain intensity after treatment [SMD = 0.36, 95%CI (−0.15, 0.87), *P* = 0.16] or during follow up [5–8 weeks after treatment: SMD = −0.11, 95%CI (−0.68, 0.46), *P* = 0.70; 12–16 weeks after treatment: SMD = 0.14, 95%CI (−0.37, 0.65), *P* = 0.59; >16 weeks after treatment: SMD = 0.25, 95%CI (−0.13, 0.63), *P* = 0.20, *I*^2^ = 0%] (Appendix Figure 7).

#### 3.4.4. Acupuncture vs. waiting list

One study (22) demonstrated that acupuncture had better improvement of responder rate, headache frequency, pain intensity, and depression score than waiting list group. Nevertheless, there was no difference between acupuncture and waiting list in improving Medical Outcomes Study Short-Form 36 mental health.

#### 3.4.5. Acupuncture vs. usual care

Schiller et al. (33) investigated the effect of acupuncture in contrast with usual care for TTH. The patients in usual care group were allowed to take preventive medication. At 6 weeks after treatment, acupuncture had better effect over usual care in reducing pain, but no difference in responder rate and headache frequency between acupuncture and usual care. At 18 weeks after treatment, acupuncture increased more responder rate than usual care, but no difference was found in headache frequency.

#### 3.4.6. Acupuncture vs. relaxation training

Söderberg et al. (60) found relaxation training was superior to acupuncture in reduction of headache frequency after treatment, but no significant differences during follow up. And there was no differences between acupuncture and relaxation training in relieving pain intensity.

#### 3.4.7. Acupuncture vs. sham acupuncture plus medication

Zhang (54) reported that, acupuncture group had higher responder rate than sham acupuncture plus medication (Estazolam 0.5 mg per day) group (*P* < 0.05), and the number of headache days and headache hours in the acupuncture group was shorter than those in sham acupuncture plus medication group (*P* < 0.05).

#### 3.4.8. Acupuncture vs. acupuncture plus exercise

Schiller et al. (33) demonstrated that no differences were found between acupuncture and acupuncture plus exercise in improving responder rate and pain intensity.

### 3.5. Adverse events

In total of 16 studies evaluated the adverse events (21–23, 29, 30, 33, 36, 49–52, 54, 56, 57, 59, 61), among which 7 RCTs reported no adverse events (29, 50, 52, 54, 57, 59, 61), and the rest 9 RCTs documented relevant adverse events. The common acupuncture related adverse events were hematoma, post-needling pain, exacerbation of headache, and acupuncture syncope reaction. Endres et al. (21) found one severe headache was possibly triggered by sham acupuncture.

### 3.6. Subgroup analysis

The results of subgroup analysis in the comparison of acupuncture vs. sham acupuncture or medication are shown in Table 2 (Appendix 7).

**Table 2:** The results of subgroup analyses.

			**No. of studies**	**No. of patients**	**RR/SMD (95% CI)**	***P***	***I^2^***
**Acupuncture vs. sham acupuncture**							
**Responder rate**	Frequency of acupuncture	Once a week	1	50	1.17 (0.46, 2.98)	0.748	0%
		2–3 times a week	2	613	1.31 (1.13, 1.51)	< 0.001	50.4%
	Total sessions	< 10 sessions	1	50	1.17 (0.46, 2.98)	0.748	0%
		≥10 sessions	2	613	1.31 (1.13, 1.51)	< 0.001	50.4%
	Needle retention	0 minutes	1	50	1.17 (0.46, 2.98)	0.748	0%
		25–30 minutes	2	613	1.31 (1.13, 1.51)	< 0.001	50.4%
**Headache frequency**	Frequency of acupuncture	Once a week	1	50	0.46 (−0.10, 1.02)	0.110	0%
		2–3 times a week	4	722	−1.19 (−2.00, −0.38)	0.004	94.9%
	Total sessions	< 10 sessions	2	90	−1.51 (−5.42, 2.40)	0.448	97.8%
		≥10 sessions	3	682	−0.62 (−1.25, 0.01)	0.055	92.5%
	Treatment duration	4–5 weeks	3	109	−1.89 (−5.05, 1.27)	0.242	96.9%
		6–8 weeks	2	663	−0.39 (−1.17, 0.40)	0.334	94.7%
	Needle retention	0 minutes	1	50	0.46 (−0.10, 1.02)	0.110	0%
		30 minutes	4	722	−1.19 (−2.00, −0.38)	0.004	94.9%
	Types of acupuncture	EA	1	40	−3.53 (−4.54, −2.52)	< 0.001	0%
		MA	4	732	−0.37 (−0.98, 0.23)	0.228	92.2%
**Pain intensity**	Frequency of acupuncture	Once a week	1	50	−0.60 (−1.17, −0.04)	0.037	0%
		2–3 times a week	5	393	−0.57 (−1.37, 0.24)	0.166	91.1%
	Total sessions	< 10 sessions	3	122	−1.15 (−2.51, 0.21)	0.096	91%
		≥ 10 sessions	3	321	0.01 (−0.44, 0.46)	0.964	65.4%
	Treatment duration	4–5 weeks	4	175	−0.81 (−1.95, 0.32)	0.161	91.5%
		6–8 weeks	2	268	−0.15 (−0.96, 0.65)	0.709	84.9%
	Needle retention	0 minute	1	50	−0.60 (−1.17, −0.04)	0.037	0%
		25–30 minutes	5	393	−0.57 (−1.37, 0.24)	0.166	91.1%
	Types of acupuncture	EA	2	74	−1.70 (−3.73, 0.34)	0.102	92.5%
		MA	4	369	−0.03 (−0.40, 0.34)	0.873	60.1%
**Acupuncture vs. medication**							
**Pain intensity**	Categories of medication	Antidepressant	2	115	−0.28 (−0.65, 0.08)	0.131	0%
		Muscle relaxant	5	584	−0.52 (−0.73, −0.31)	< 0.001	30.1%
		Analgesics	1	82	−1.35 (−1.83, −0.87)	< 0.001	0%
		Antidepressant **+** Analgesics	1	50	−1.09 (−1.69, −0.50)	< 0.001	0%
**Depression**	Categories of medication	Antidepressant	4	239	−0.18 (−0.65, 0.29)	0.451	70%
		Muscle relaxant	1	60	−1.19 (−1.75, −0.64)	< 0.001	0%
**Anxiety**	Categories of medication	Antidepressant	3	177	0.14 (−0.22, 0.50)	0.442	32.4%
		Muscle relaxant	1	60	−0.94 (−1.47, −0.40)	0.001	0%

No, number; EA, electro-acupuncture; MA, manual acupuncture; CI, confidence interval.

In comparison of acupuncture vs. sham acupuncture, we conducted subgroup analyses based on frequency of acupuncture, total sessions, treatment duration, needle retention and types of acupuncture.

Acupuncture with a frequency at 2–3 times a week was superior to sham acupuncture in improving responder rate and headache frequency. And once a week acupuncture treatment had better efficacy than sham acupuncture in relieving pain intensity. As for total sessions, acupuncture with total sessions ≥10 was more effective than sham acupuncture to improve responder rate. With regard to needle retention, acupuncture with 25–30 minutes needle retention could improve more responder rate and headache frequency than sham acupuncture. Whereas acupuncture with retaining needle eased more pain intensity than sham acupuncture. For types of acupuncture, EA was more effective than sham acupuncture in the reduction of headache frequency.

Subgroup analyses in comparison of acupuncture vs. medication were performed according to categories of medication. The results demonstrated that acupuncture had better effect than muscle relaxants in improvement of pain intensity, depression and anxiety. While there was no difference between acupuncture and antidepressants in relieving the above symptoms.

### 3.7. Sensitivity analysis

Sensitivity analysis was performed by omitting study one by one. Except for responder rate [*acupuncture vs. sham acupuncture* (after treatment)], the pooled results of the rest outcomes were robust (Appendix 8).

### 3.8. Publication bias

Due to insufficient studies of primary outcome (*n* ≤ 10), we failed to explore the publication bias.

### 3.9. Assessment of evidence

The outcomes of pain intensity [>16 weeks after treatment (acupuncture vs. sham acupuncture)] were rated as “High” certainty. The outcomes of responder rate [after treatment, 1–4 weeks after treatment, 5–8 weeks after treatment, >16 weeks after treatment (acupuncture vs. sham acupuncture)] and pain intensity [5–8 weeks after treatment (acupuncture vs. sham acupuncture)] were rated as “Moderate” certainty, while the remaining outcomes were considered as “Low” or “Very low” certainty. The certainty of evidence was primarily downgraded by the inconsistency and imprecision of results in the included studies. The summary of findings is presented in Appendix 10.

## 4. Discussion

### 4.1. The effect of acupuncture for TTH

In the present study, the results showed that acupuncture had better efficacy than sham acupuncture in improvement of responder rate and headache frequency, the findings were consistent with previous systematic reviews from Linde (20, 25). In addition, acupuncture was more effective than medication to alleviate pain intensity. Patients receiving acupuncture fared significantly better than waiting list in outcomes of responder rate, headache frequency, pain intensity, and depression score. Acupuncture did not differ from exercise in ameliorating pain intensity after treatment. There was no difference between acupuncture and relaxation training in relieving pain intensity. Moreover, acupuncture had long-term therapeutic effect to improve responder rate and pain intensity, and the improvement persisted for at least 16 weeks. According to subgroup analysis, acupuncture was not effective than antidepressants in relieving pain intensity, depression and anxiety. Due to the undesirable side effects of antidepressants (65–67), acupuncture may be a reasonable option for patients with TTH.

Different parameters (frequency, total sessions, treatment duration, retention time, and types of acupuncture etc.) are important to the effect of acupuncture treatment for TTH. As for total sessions, the effect sizes of acupuncture (≥10 sessions) were better than sham acupuncture. In terms of the types of acupuncture, EA could reduce more headache frequency than MA. Acupuncture with a frequency at 2–3 times a week was superior to sham acupuncture in improving responder rate and headache frequency. And acupuncture treatment once a week had better efficacy than sham acupuncture in relieving pain intensity. With regard to needle retention, acupuncture with 25–30 minutes needle retention could improve more responder rate and headache frequency than sham acupuncture. Whereas acupuncture without needle retention eased the pain intensity better than sham acupuncture. However, these findings should be treated with caution, and more rigorous RCTs are needed to explore optimal acupuncture protocol.

### 4.2. Implications for future studies

The overall risk of bias of 4 RCTs was considered as low risk of bias, the remaining 26 studies were rated as some concerns. The main problems existed in neglection of the allocation concealment, no ITT analysis, lack of the appropriate blinding methods, no details of drop-outs, no pre-specified protocol and registration information. Therefore, investigators should pay attention to these issues during the whole process of clinical trial (68). The Consolidated Standards of Reporting Trials (CONSORT) (69) was recommended to improve the reporting quality of RCTs.

Based on the assessment of STRICTA, most included studies did not report the following items: (1b) the reasoning of acupuncture treatment. (2a) the number of needle insertions, (2c) depth of insertion, (2d) response sought, (4a) details of other interventions administered to the acupuncture group, (5) information about the acupuncturist's background, (6b) rationale for the control or comparator. To improve the reporting quality of interventions in clinical trials of acupuncture, STRICTA (45) should be used.

Among included studies, the meta-analysis involved 3 comparisons including acupuncture vs. sham acupuncture, acupuncture vs. medication, acupuncture vs. exercise. And descriptive analysis included 5 comparisons such as acupuncture vs. waiting list, acupuncture vs. usual care, acupuncture vs. relaxation training, acupuncture vs. sham acupuncture plus medication, acupuncture vs. acupuncture plus exercise. Nevertheless, due to limited RCTs, high heterogeneity and low or very low certainty of evidence, the above results should be interpreted with caution. More RCTs comparing acupuncture with other active control groups (medication, exercise, etc.) are needed.

### 4.3. Strengths and limitations

This is the lasted systematic review and meta-analysis of acupuncture for TTH. We comprehensively assessed the risk of bias of included studies using ROB 2, utilized STRICTA to appraise the reporting quality of interventions in clinical trials of acupuncture, and employed GRADE to evaluate the certainty of evidence. Meanwhile, this systematic review and meta-analysis was conducted in accordance with AMSTAR 2 and reported complying the PRISMA.

However, some limitations should be considered. First, since the certainty of majority outcomes was assessed as low or very low, and the risk of bias in most included studies was some concerns, the findings should be treated with discretion. Second, owing to limited studies, the optimal protocol of acupuncture for patients with TTH was not identified. More RCTs are required to investigate the optimal protocol of acupuncture for TTH in the future. Third, the majority of the included patients were from China, which might limit the applicability of the findings to other races.

## 5. Conclusion

Acupuncture may be an effective and safe treatment for patients with TTH. Notwithstanding, due to the low and very low certainty of most evidence and high heterogeneity, more rigorous RCTs are needed to verify the effect and safety of acupuncture in the management of TTH.

## Data availability statement

The original contributions presented in the study are included in the article/Supplementary material, further inquiries can be directed to the corresponding authors.

## Author contributions

W-lK, RF, and X-jX designed the protocol and drafted the manuscript. YF, JL, and R-jJ revised the manuscript. D-lZ, JS, and Y-xL screened the articles, extracted data, and conducted data synthesis. All authors reviewed and approved this review.
