# Supplementary material for: Acupuncture for tension-type headache: a systematic review and meta-analysis of randomized controlled trials
Source: Front Neurol. 2023 May 10;13:943495. doi: 10.3389/fneur.2022.943495 (PMC10208222; doi:10.3389/fneur.2022.943495)
Supplement: Supplementary file 1 [file Data_Sheet_1.docx]

# *Appendix*

**1: Search strategy**

**1.1 For English databases**

|  | ***Search strategy*** |
| --- | --- |
| *Randomized Controlled Trial* | 1. "Randomized Controlled Trial"[Publication Type] 2. "randomized" [Title/Abstract] 3. "placebo" [Title/Abstract] 4. #1 OR #2 OR #3 |
| *Tension-Type Headache* | 1. " Tension-Type Headache" [MeSH Terms] 2. " Headache*, Tension-Type " [Title/Abstract] 3. " Tension Type Headache" [Title/Abstract] 4. " Tension-Type Headaches" [Title/Abstract] 5. " Idiopathic Headache*" [Title/Abstract] 6. " Headache*, Idiopathic" [Title/Abstract] 7. " Stress Headache*" [Title/Abstract] 8. " Headache*, Stress" [Title/Abstract] 9. " Tension Headache*" [Title/Abstract] 10. " Headache*, Tension " [Title/Abstract] 11. " Psychogenic Headache*" [Title/Abstract] 12. " Headache*, Psychogenic" [Title/Abstract] 13. " Tension-Vascular Headache* " [Title/Abstract] 14. " Headache*, Tension-Vascular " [Title/Abstract] 15. " Tension Vascular Headache" [Title/Abstract] 16. #5 OR #6 OR #7 OR #8 OR #9 OR #10 OR #11 OR #12 OR #13 OR #14 OR #15 OR #16 OR #17 OR #18 OR #19 |
| *Acupuncture* | 1. " Acupuncture " [MeSH Terms] 2. " Pharmacopuncture " [Title/Abstract] 3. #21 OR #22 |
|  | 1. #4 OR #20 OR #23 |

**1.2 For Chinese databases**

|  | ***Search strategy*** |
| --- | --- |
| *#*1 | 主题: (紧张性头痛 OR 紧张型头痛 OR 头痛 OR 肌收缩性头痛) |
| *#*2 | 主题: (针灸 OR 针刺 OR 针灸疗法 OR 针刺疗法 OR 电针) |
|  | #1 AND #2 |

**2: Excluded studies**

| **No.** | **First Author and**  **Year of**  **publication** | **Title** | **Excluded Reason** |
| --- | --- | --- | --- |
| 1 | Airaksinen.1992 | EFFECTS OF THE ELECTRICAL STIMULATION OF MYOFASCIAL TRIGGER POINTS WITH TENSION HEADACHE | Unavailable data |
| 2 | Akhmadeeva.2015 | A double blind randomized placebo controlled trial for non-Invasive dynamic trans-cutaneous electrical nerves stimulation in management of tension type headaches: the first results | Diagnostic criteria didn’t be mentioned |
| 3 | Carlsson.1990 | Health status in patients with tension headache treated with acupuncture or physiotherapy | Not meet diagnostic criteria |
| 4 | Chen.2017 | Clinical observation of acupuncture on episodic tension-type headache | Unavailable data |
| 5 | Chen.2008 | Effect of acupuncture on tension-type headache | Use two or more different types of acupuncture |
| 6 | Fu.2013 | Clinical Observation on Acupuncture for Tension-type Headache | Unavailable data |
| 7 | Georgoudis.2018 | The effect of physiotherapy and acupuncture on psychocognitive, somatic, quality of life, and disability characteristics in TTH patients | Intervention combined with exercise |
| 8 | Gildir.2019 | A randomized trial of trigger point dry needling versus sham needling for chronic tension-type headache | No details on control group |
| 9 | Gu.2017 | Evaluation of the therapeutic effect of acupuncture on tension headache | No details on diagnostic criteria |
| 10 | Hansen.1985 | Acupuncture treatment of chronic tension headache -- a controlled cross-over trial | Unavailable data |
| 11 | Karakurum.2001 | The `dry-needle technique': intramuscular stimulation in  tension-type headache | Unavailable data |
| 12 | Karst.2000 | Pressure pain threshold and needle acupuncture in chronic tension-type headache - a double-blind placebo-controlled study | Overlapping publication |
| 13 | Lao.2004 | Observation upon clinical therapeutic effects of the Awaking the brains method of acupuncture in the treatment of tension headache | Unavailable data |
| 14 | Liu.2014 | Clinical observation of cluster needling combined with distal acupoint selection in the treatment of chronic tension-type headache | Unavailable data |
| 15 | Lu.2018 | Acupuncture for Chronic Tension-type Headache: A Study on Clinical Effectiveness and Differentially Expressed Genes | Duplicate publication |
| 16 | Luo.2021 | Clinical effect of the treatment of chronic tension headache by acupuncture needle-retaining at Ashi point in head | Diagnostic criteria didn’t be mentioned |
| 17 | Melchart.2005 | The acupuncture randomised trial (ART) for tension-type headache – details of the treatment | Duplicate publication |
| 18 | So¨derberg.2011 | Subjective Well-being in Patients with Chronic Tension-type Headache: Effect of Acupuncture, Physical Training, and Relaxation Training | Unavailable data |
| 19 | Sliva.2012 | Acupuncture for tension-type headache in pregnancy: A prospective, randomized, controlled study | Intervention combined with exercise |
| 20 | Wylie.1997 | Does psychological testing help to predict the response to acupuncture or massage/relaxation therapy in patients presenting to a general neurology clinic with headache? | Enrolled both migraine and tension type headache |
| 21 | Xu.2000 | Observation on the therapeutic effect of acupuncture and western medicine in the treatment of neck muscle tension headache | Diagnostic criteria didn’t be mentioned |
| 22 | Zhang.2011 | Clinical Observation on Tension-type Headache Treated by Acupuncture Point Selection Based on Root-knot Specimen Theory | Unavailable data |
| 23 | Zhang.2018 | The effect of acupuncture on the brain function of patients with chronic tension-type headache | Unavailable data |
| 24 | Zhang.2018 | Clinical Study on the Treatment of Eight Scalp Needles on Tension Type Headache | Use two or more different types of acupuncture |
| 25 | Zheng.2017 | Clinical observation on Tension—type headache by balanced acupuncture treatment | Use two or more different types of acupuncture |
| 26 | Zheng.2020 | Clinical Observation on Treatment of Tension-type Headache with Balance Acupuncture | Use two or more different types of acupuncture |

**3: Acupoints selection**

| **No.** | **First Author and Year of publication** | **Acupoints** |
| --- | --- | --- |
| 1 | Chassot.2015 | Anatomic needling points. Sixteen needles were inserted, from cephalad to caudal, bilaterally: superior and central area of the tip of the triangular fossa in junction of the superior and inferior crus of the antihelix (ear); helix root (ear); medial aspect of splenius capitis and semispinalis capitis muscles (C1-C2 level); lateral aspect of trapezius; semispinalis capitis muscle (C6-T1 level); levator scapulae muscles (C6-T1 level); abductor pollicis brevis and dorsal interossei muscles (hands). |
| 2 | Duan.2013 | Baihui (GV20), Taiyang (EX-HN5), Touwei(ST8), Fengchi (GB20), Lieque(LU7), Taichong (LR3), Zusanli(ST36), Sanyinjiao(SP6), “Ashi” points. |
| 3 | Deng.2013 | Sishencong (EX-HN1), Fengchi (GB20), Taiyang (EX-HN5) + optional points. |
| 4 | Endres.2007 | Baihui (GV20), Hegu (LI4), Taichong (LR3) [or Xingjian (LR2)], Fengchi (GB20) [or Tianzhu (BL10)] + optional points. |
| 5 | Guo.2019 | Baihui (GV20), Yintang (GV29), Touwei(ST8), Fengchi (GB20), Shuaigu(GB8), Taichong (LR3), Shenmen(HT7), “Ashi” points. + optional acupoints. |
| 6 | Guo.2020 | Baihui (GV20), Taiyang (EX-HN5), Fengchi (GB20), Hegu (LI4), “Ashi” points + optional points. |
| 7 | Huang.2012 | 1. Fengchi(GB20), Baihui (GV20), Xuanlu (GB5), Xiaxi(GB43) Xingjian(LR2) or 2. Baihui (GV20) Shenshu (BL23),Fuliu(KI7), Xuanzhong(GB39) Or 3. Fenglong (ST40), Zhongwan (CV12), Yinglingquan (SP9) or 4. Baihui (GV20), Qihai (BL24), Ganshu (BL18), Pishu (BL20), Hegu (LI4), Zusanli(ST36). |
| 8 | Jeon.2019 | Wangu (GB12), Fengchi(GB20). |
| 9 | Karst.2001 | Fengchi (GB20), Hegu (LI4), Taichong(LR3), Shuaigu(GB8), Yangbai(GB14), Jianjing(GB21), Zulinqi(GB41), Cuanzhu(BL2), Tianzhu(BL10), Kunlun(BL60), Lieque(LU7), Touwei(ST8), Zusanli(ST36), Neiting (ST44), Baihui(DU20), Extra1. |
| 10 | Koran.2021 | Taichong(LR3), Taiyuan(LU9), Taixi(KI3), Taibai(SP3), Hegu (LI4), Zusanli(ST36), Quchi(LI11) |
| 11 | Kwak.2008 | Chosen individually by the physicians on the basis of traditional Chinese medicine diagnosis. |
| 12 | Liu.2020 | (EX-B2)2-4, Fengchi(GB20), Neiguan (PC6), Gongsun (SP4), Lieque(LU7), Zhaohai (KI6), Waiguan(SJ5), Zulinqi(GB41), Shenmai (BL62), Houxi(SI3). |
| 13 | Melchart.2005 | Fengchi (GB20), Jianjing(GB21), Taichong(LR3) + optional points |
| 14 | Nie.2019 | Trigger points. |
| 15 | Schiller.2021 | Baihui (GV20), Taiyang (EX-HN5), Fengchi (GB20), Hegu (LI4), Neiting (ST44), Yintang (GV29), Zulinqi (GB41), Waiguan (SJ5), Kunlun (BL60), Houxi (SI3), Taichong (LR3), Neiguan (PC6), Sishencong (EX-HN1), “Ashi” points. |
| 16 | Söderberg.2006 | Fengchi (GB20), Yangbai (GB 14), Hegu (LI4), Neiting(ST44) + optional points: Neiguan (PC6), Daling(PC7), Sanyinjiao(SP6), Yanglinquan(GB34), Touwei(ST8), Dangyang(EX-HN2), Sishencong (EX-HN1). |
| 17 | Tavola.1992 | The choice of points was made on an individual basis relative to the evaluation of the patient’s ‘energy’ status according to the criteria of traditional Chinese acupuncture. |
| 18 | Wang.2022 | Shenting(DU24), Meichong (BL3), Toulinqi (GB15), Touwei (ST8), Baihui (GV20), Hanyan(GB4),Naokong (GB19), Naohu (GV17) |
| 19 | Wang.2018 | Shangwan(RN13, Zhongwan(RN12, Xiawan(RN10, Tianshu (ST25), Qihai(CV5), Neiguan (PC6), Zusanli(ST36). |
| 20 | White.2000 | Fengchi (GB20), Hegu (LI4) + optional points. |
| 21 | Wu.2016 | Sishencong (EX-HN1), Shenting(DU24), Yintang (GV29), Neiguan (PC6), Sanyinjiao(SP6). |
| 22 | White.1996 | Hegu (LI4), Fengchi (GB20), Jianjing(GB21), Yintang (GV29), Taiyang (EX-HN5), Yangbai(GB14). |
| 23 | Xiang.2015 | Fenglong(ST40), Zusanli(ST36), Taiyang (EX-HN5), Touwei(ST8), Baihui (GV20)，Fengchi (GB20), Jinjiaji(EX-B2), Shenmen(HT7), Taichong (LR3). |
| 24 | Xue.2004 | Hegu(L14), Waiguan (SJ5), Fenglong(ST40), Kunlun(GB60), Taichong(LR3), Xingjian(LR2), Hegu(LI4), Lieque(LU7), Saninjiao(SP6), Zusanli(ST36) Hegu(LI4), Waiguan(SJ5), Taichong(LR3), Taixi(KI3), Waiguan(SJ5). |
| 25 | Yang.2020 | Neiguan (PC6), Sanyinjiao(SP6), Taichong (LR3), Baihui (GV20), Yintang (GV29), Trigger points. |
| 26 | Zhang.2009 | Baihui (GV20), Shenting(DU24), Touwei(ST8), Taiyang (EX-HN5), Taichong (LR3). |
| 27 | Zhang.2021 | Baihui (GV20), Fengchi (GB20), Shuaigu(GB8), Jiaosun(SJ20), Taiyang (EX-HN5), Lieque(LU7), Hegu (LI4), Taichong (LR3), “Ashi”points |
| 28 | Zhou.2015 | Baihui (GV20), Yintang (GV29), Taiyang (EX-HN5), Hegu (LI4), Taichong (LR3), Neiguan (PC6) + optional points. |
| 29 | Zhu.2013 | Baihui (GV20), Fengchi (GB20), Taiyang (EX-HN5), Touwei(ST8), Taichong (LR3), Geshu(BL18), Xuehai(SP10), Hegu (LI4), “Ashi” points + optional points. |
| 30 | Zheng.2022 | Baihui (GV20), Taiyang (EX-HN5), Fengchi (GB20), Taichong (LR3), Hegu (LI4). |

**4:** **Completeness and transparency of reporting of acupuncture (STRICTA 2010 checklist)**

**Chassot.2015**

| **Item** | **Detail** |
| --- | --- |
| **1. Acupuncture rationale** | 1a) Style of acupuncture |
|  | No report |
|  | 1b) Reasoning for treatment provided, based on historical context, literature sources, and/or consensus methods, with references where appropriate |
|  | No report |
|  | 1c) Extent to which treatment was varied |
|  | All patients receiving the same treatment at all sessions |
| **2. Details of needling** | 2a) Number of needle insertions per subject per session (mean and range where relevant) |
|  | A total of sixteen needles were used per subject per session. |
|  | 2b) Names (or location if no standard name) of points used (uni/bilateral) |
|  | Anatomic needling points. Sixteen needles were inserted, from cephalad to caudal, bilaterally: superior and central area of the tip of the triangular fossa in junction of the superior and inferior crus of the antihelix (ear); helix root (ear); medial aspect of splenius capitis and semispinalis capitis muscles (C1-C2 level); lateral aspect of trapezius; semispinalis capitis muscle (C6-T1 level); levator scapulae muscles (C6-T1 level); abductor pollicis brevis and dorsal interossei muscles (hands). |
|  | 2c) Depth of insertion, based on a specified unit of measurement, or on a particular tissue level |
|  | No report |
|  | 2d) Response sought (e.g., de qi or muscle twitch response) |
|  | No report |
|  | 2e) Needle stimulation (e.g., manual, electrical) |
|  | Electrical |
|  | 2f) Needle retention time |
|  | 30 minutes |
|  | 2g) Needle type (diameter, length, and manufacturer or material) |
|  | We used acupuncture needles with guide tubes (Suzhou Huanqiu Acupuncture Medical Appliance Co. Ltd., 218, China) that were 40 mm in length and 0.25 mm in diameter. |
| **3. Treatment regimen** | 3a) Number of treatment sessions |
|  | Ten sessions. |
|  | 3b) Frequency and duration of treatment sessions |
|  | Twice per week, 5 weeks |
| **4. Other components**  **of treatment** | 4a) Details of other interventions administered to the acupuncture group (e.g., moxibustion, cupping, herbs, exercises, lifestyle advice) |
|  | No report |
|  | 4b) Setting and context of treatment, including instructions to practitioners, and information and explanations to patients |
|  | No report |
| **5. Practitioner**  **background** | 5) Description of participating acupuncturists (qualification or professional affiliation, years in acupuncture practice, other relevant experience) |
|  | All treatment sessions were administered by the same trained (F.S.) and experienced (10 years) acupuncturist physician. |
| **6. Control or comparator**  **interventions** | 6a) Rationale for the control or comparator in the context of the research question, with sources that justify this choice |
|  | For the sham intervention, we used an electroacupuncture device, which was adjusted beforehand to prevent the current from passing through the electrodes. No needling was used at all. The electrical connection between the stimulator and the patient was broken at the output jack plug of the stimulator so that no current could pass to the patient. The patients were informed that this was a high-frequency, low-intensity stimulation and that they would most likely feel no sensation from it. The electrodes were placed in the same areas as the real stimulation. The nerve stimulation unit was left in front of the patient for 30 minutes. This positioning ensured that the flashing diode that simulated the electrical stimulus was both visible and audible. The patients were sitting comfortably, so they could see the device’s light sign. |
|  | 6b) Precise description of the control or comparator. If sham acupuncture or any other type of acupuncture-like control is used, provide details as for Items 1 to 3 above. |
|  | Sham acupuncture, the frequency, sessions and duration were same as intervention group |

**Deng.2013**

| **Item** | **Detail** |
| --- | --- |
| **1. Acupuncture rationale** | 1a) Style of acupuncture |
|  | Traditional Chinese Medicine |
|  | 1b) Reasoning for treatment provided, based on historical context, literature sources, and/or consensus methods, with references where appropriate |
|  | Practical internal medicine of traditional Chinese medicine [M]. Shanghai: Shanghai Science and Technology Press. |
|  | 1c) Extent to which treatment was varied |
|  | Use of a fixed set of points to be combined with a set of points to be used flexibly |
| **2. Details of needling** | 2a) Number of needle insertions per subject per session (mean and range where relevant) |
|  | No report |
|  | 2b) Names (or location if no standard name) of points used (uni/bilateral) |
|  | Bilateral: Sishencong (EX-HN1), Fengchi (GB20), Taiyang (EX-HN5) + optional points. |
|  | 2c) Depth of insertion, based on a specified unit of measurement, or on a particular tissue level |
|  | 0.5-0.8 cun |
|  | 2d) Response sought (e.g., de qi or muscle twitch response) |
|  | De qi |
|  | 2e) Needle stimulation (e.g., manual, electrical) |
|  | Electrical |
|  | 2f) Needle retention time |
|  | 30 minutes |
|  | 2g) Needle type (diameter, length, and manufacturer or material) |
|  | 1 cun Huatuo brand stainless steel disposable sterile acupuncture needle (0. 35mm×25mm) |
| **3. Treatment regimen** | 3a) Number of treatment sessions |
|  | 10 sessions |
|  | 3b) Frequency and duration of treatment sessions |
|  | Once a day, 10 days |
| **4. Other components**  **of treatment** | 4a) Details of other interventions administered to the acupuncture group (e.g., moxibustion, cupping, herbs, exercises, lifestyle advice) |
|  | No report |
|  | 4b) Setting and context of treatment, including instructions to practitioners, and information and explanations to patients |
|  | No report |
| **5. Practitioner**  **background** | 5) Description of participating acupuncturists (qualification or professional affiliation, years in acupuncture practice, other relevant experience) |
|  | No report |
| **6. Control or comparator**  **interventions** | 6a) Rationale for the control or comparator in the context of the research question, with sources that justify this choice |
|  | No report |
|  | 6b) Precise description of the control or comparator. If sham acupuncture or any other type of acupuncture-like control is used, provide details as for Items 1 to 3 above. |
|  | Acetaminophen (0.5g, qid, 10 days) + Amitriptyline (25mg, qn, 10 days) |

**Duan.2013**

| **Item** | **Detail** |
| --- | --- |
| **1. Acupuncture rationale** | 1a) Style of acupuncture |
|  | Traditional Chinese Medicine |
|  | 1b) Reasoning for treatment provided, based on historical context, literature sources, and/or consensus methods, with references where appropriate |
|  | Diagnosis and curative effect evaluation standard of headache; National Encephalopathy Emergency Collaborative Group. |
|  | 1c) Extent to which treatment was varied |
|  | All patients receiving the same treatment at all sessions |
| **2. Details of needling** | 2a) Number of needle insertions per subject per session (mean and range where relevant) |
|  | No report |
|  | 2b) Names (or location if no standard name) of points used (uni/bilateral) |
|  | Bilateral: Baihui (GV20), Taiyang (EX-HN5), Touwei(ST8), Fengchi (GB20), Lieque(LU7), Taichong (LR3), Zusanli(ST36), Sanyinjiao(SP6), “Ashi” points. |
|  | 2c) Depth of insertion, based on a specified unit of measurement, or on a particular tissue level |
|  | Fengchi (GB20) was penetrated about 1.2 cun, and the remaining points were conventional depth of insertion. |
|  | 2d) Response sought (e.g., de qi or muscle twitch response) |
|  | De qi |
|  | 2e) Needle stimulation (e.g., manual, electrical) |
|  | Manual |
|  | 2f) Needle retention time |
|  | 40 minutes |
|  | 2g) Needle type (diameter, length, and manufacturer or material) |
|  | Length is 2.5 cun |
| **3. Treatment regimen** | 3a) Number of treatment sessions |
|  | 21 sessions |
|  | 3b) Frequency and duration of treatment sessions |
|  | Once a day, 3 weeks |
| **4. Other components**  **of treatment** | 4a) Details of other interventions administered to the acupuncture group (e.g., moxibustion, cupping, herbs, exercises, lifestyle advice) |
|  | No report |
|  | 4b) Setting and context of treatment, including instructions to practitioners, and information and explanations to patients |
|  | No report |
| **5. Practitioner**  **background** | 5) Description of participating acupuncturists (qualification or professional affiliation, years in acupuncture practice, other relevant experience) |
|  | No report |
| **6. Control or comparator**  **interventions** | 6a) Rationale for the control or comparator in the context of the research question, with sources that justify this choice |
|  | No report |
|  | 6b) Precise description of the control or comparator. If sham acupuncture or any other type of acupuncture-like control is used, provide details as for Items 1 to 3 above. |
|  | Eperisone Hydrochloride Tablets, three times a day, 50mg, oral after meal |

**Endres.2007**

| **Item** | **Detail** |
| --- | --- |
| **1. Acupuncture rationale** | 1a) Style of acupuncture |
|  | Traditional Chinese Medicine |
|  | 1b) Reasoning for treatment provided, based on historical context, literature sources, and/or consensus methods, with references where appropriate |
|  | Literature analyses: Approximately 40 German, U.S., and Chinese (in English translation) acupuncture textbooks were analyzed, and the most commonly used acupuncture points for the respective disorders were extracted |
|  | 1c) Extent to which treatment was varied |
|  | Use of a fixed set of points to be combined with a set of points to be used flexibly |
| **2. Details of needling** | 2a) Number of needle insertions per subject per session (mean and range where relevant) |
|  | 10-25 |
|  | 2b) Names (or location if no standard name) of points used (uni/bilateral) |
|  | Bilateral: Baihui (GV20), Hegu (LI4), Taichong (LR3) [or Xingjian (LR2)], Fengchi (GB20) [or Tianzhu (BL10)] + optional points |
|  | 2c) Depth of insertion, based on a specified unit of measurement, or on a particular tissue level |
|  | Needles were inserted 2–30 mm |
|  | 2d) Response sought (e.g., de qi or muscle twitch response) |
|  | De qi |
|  | 2e) Needle stimulation (e.g., manual, electrical) |
|  | Manual |
|  | 2f) Needle retention time |
|  | 30 minutes |
|  | 2g) Needle type (diameter, length, and manufacturer or material) |
|  | Diameter 0.25–0.30 mm, length 25–40 mm |
| **3. Treatment regimen** | 3a) Number of treatment sessions |
|  | 10 sessions |
|  | 3b) Frequency and duration of treatment sessions |
|  | Twice a week, 6 weeks |
| **4. Other components**  **of treatment** | 4a) Details of other interventions administered to the acupuncture group (e.g., moxibustion, cupping, herbs, exercises, lifestyle advice) |
|  | During the study, patients were allowed to take only one of their pre-baseline oral headache analgesics. They were not allowed to change this analgesic |
|  | 4b) Setting and context of treatment, including instructions to practitioners, and information and explanations to patients |
|  | All patients receiving acupuncture treatment were informed as follows: “It has not yet been proven whether the needles must be inserted at certain, precisely defined locations in order for acupuncture to be effective. Therefore, in this study, a traditional Chinese form of acupuncture will be compared with a form of acupuncture developed especially for this study. |
| **5. Practitioner**  **background** | 5) Description of participating acupuncturists (qualification or professional affiliation, years in acupuncture practice, other relevant experience) |
|  | All physicians agreed to be visited repeatedly by independent clinical monitors (good clinical practice s |
| **6. Control or comparator**  **interventions** | 6a) Rationale for the control or comparator in the context of the research question, with sources that justify this choice |
|  | Sham acupuncture avoided all known verum points or meridians for needling; moreover, no points on the head could be used in the sham group. Needles were inserted superficially (1–3 mm) and were not stimulated, so as to avoid De Qi in the sham group. |
|  | 6b) Precise description of the control or comparator. If sham acupuncture or any other type of acupuncture-like control is used, provide details as for Items 1 to 3 above. |
|  | Sham acupuncture, the frequency, sessions and duration were same as intervention group |

**Guo.2020**

| **Item** | **Detail** |
| --- | --- |
| **1. Acupuncture rationale** | 1a) Style of acupuncture |
|  | Traditional Chinese Medicine |
|  | 1b) Reasoning for treatment provided, based on historical context, literature sources, and/or consensus methods, with references where appropriate |
|  | Acupuncture and moxibustion [M]. Beijing: People's Health Publishing House, 2012. |
|  | 1c) Extent to which treatment was varied |
|  | Use of a fixed set of points to be combined with a set of points to be used flexibly. |
| **2. Details of needling** | 2a) Number of needle insertions per subject per session (mean and range where relevant) |
|  | Nine |
|  | 2b) Names (or location if no standard name) of points used (uni/bilateral) |
|  | Baihui (GV20), Taiyang (EX-HN5), Fengchi (GB20), Hegu (LI4), “Ashi” points + optional points. |
|  | 2c) Depth of insertion, based on a specified unit of measurement, or on a particular tissue level |
|  | 30 mm |
|  | 2d) Response sought (e.g., de qi or muscle twitch response) |
|  | No report |
|  | 2e) Needle stimulation (e.g., manual, electrical) |
|  | Manual |
|  | 2f) Needle retention time |
|  | 30 min |
|  | 2g) Needle type (diameter, length, and manufacturer or material) |
|  | Diameter 0.25mm, length 40mm |
| **3. Treatment regimen** | 3a) Number of treatment sessions |
|  | 12 sessions |
|  | 3b) Frequency and duration of treatment sessions |
|  | Once every other one day, four weeks |
| **4. Other components**  **of treatment** | 4a) Details of other interventions administered to the acupuncture group (e.g., moxibustion, cupping, herbs, exercises, lifestyle advice) |
|  | No report |
|  | 4b) Setting and context of treatment, including instructions to practitioners, and information and explanations to patients |
|  | No report |
| **5. Practitioner**  **background** | 5) Description of participating acupuncturists (qualification or professional affiliation, years in acupuncture practice, other relevant experience) |
|  | No report |
| **6. Control or comparator**  **interventions** | 6a) Rationale for the control or comparator in the context of the research question, with sources that justify this choice |
|  | No report |
|  | 6b) Precise description of the control or comparator. If sham acupuncture or any other type of acupuncture-like control is used, provide details as for Items 1 to 3 above. |
|  | Eperisone Hydrochloride Tablets (50mg, three times a day) + Flunarizine hydrochloride (5mg, orally before bedtime, once a day); Four weeks. |

**Guo.2019**

| **Item** | **Detail** |
| --- | --- |
| **1. Acupuncture rationale** | 1a) Style of acupuncture |
|  | Traditional Chinese Medicine |
|  | 1b) Reasoning for treatment provided, based on historical context, literature sources, and/or consensus methods, with references where appropriate |
|  | The acupoints and related operations were selected and operated in accordance with the "Tenth Five-Year" national planning textbook for general higher education, "Acupuncture and Moxibustion", which was published by China Traditional Chinese Medicine Press in 2002 and edited by Shi Xuemin. |
|  | 1c) Extent to which treatment was varied |
|  | Use of a fixed set of points to be combined with a set of points to be used flexibly |
| **2. Details of needling** | 2a) Number of needle insertions per subject per session (mean and range where relevant) |
|  | No report |
|  | 2b) Names (or location if no standard name) of points used (uni/bilateral) |
|  | Bilateral: Baihui (GV20), Yintang (GV29), Touwei(ST8), Fengchi (GB20), Shuaigu(GB8), Taichong (LR3), Shenmen(HT7), “Ashi” points + optional acupoints. |
|  | 2c) Depth of insertion, based on a specified unit of measurement, or on a particular tissue level |
|  | Baihui (GV20), Yintang (GV29), Touwei(ST8), Shuaigu(GB8), Ashi points are pierced 0.3-0.5 cun, Fengchi (GB20) is pierced straight 0.5-1.0 inch, Taichong (LR3), Shenmen (HT7) are pierced 0.3-0.8 cun |
|  | 2d) Response sought (e.g., de qi or muscle twitch response) |
|  | No report |
|  | 2e) Needle stimulation (e.g., manual, electrical) |
|  | Manual |
|  | 2f) Needle retention time |
|  | 30 min |
|  | 2g) Needle type (diameter, length, and manufacturer or material) |
|  | Diameter 0.25mm, length 25mm |
| **3. Treatment regimen** | 3a) Number of treatment sessions |
|  | 14 sessions |
|  | 3b) Frequency and duration of treatment sessions |
|  | Once every two days, 4 weeks |
| **4. Other components**  **of treatment** | 4a) Details of other interventions administered to the acupuncture group (e.g., moxibustion, cupping, herbs, exercises, lifestyle advice) |
|  | No report |
|  | 4b) Setting and context of treatment, including instructions to practitioners, and information and explanations to patients |
|  | No report |
| **5. Practitioner**  **background** | 5) Description of participating acupuncturists (qualification or professional affiliation, years in acupuncture practice, other relevant experience) |
|  | No report |
| **6. Control or comparator**  **interventions** | 6a) Rationale for the control or comparator in the context of the research question, with sources that justify this choice |
|  | No report |
|  | 6b) Precise description of the control or comparator. If sham acupuncture or any other type of acupuncture-like control is used, provide details as for Items 1 to 3 above. |
|  | Deanxit, each piece of drug be taken at morning and noontime, 4 weeks |

**Huang.2012**

| **Item** | **Detail** |
| --- | --- |
| **1. Acupuncture rationale** | 1a) Style of acupuncture |
|  | Traditional Chinese Medicine |
|  | 1b) Reasoning for treatment provided, based on historical context, literature sources, and/or consensus methods, with references where appropriate |
|  | Treatment methods were chosen individually by the physicians on the basis of traditional Chinese medicine diagnosis. |
|  | 1c) Extent to which treatment was varied |
|  | Fully individualized treatment protocols within which each patient receives a unique and evolving diagnosis and treatment. |
| **2. Details of needling** | 2a) Number of needle insertions per subject per session (mean and range where relevant) |
|  | No report |
|  | 2b) Names (or location if no standard name) of points used (uni/bilateral) |
|  | 1. Fengchi(GB20), Baihui (GV20), Xuanlu (GB5), Xiaxi(GB43) Xingjian(LR2) or 2. Baihui (GV20) Shenshu (BL23),Fuliu(KI7), Xuanzhong(GB39) Or 3. Fenglong (ST40), Zhongwan (CV12), Yinglingquan (SP9) or 4. Baihui (GV20), Qihai (BL24), Ganshu (BL18), Pishu (BL20), Hegu (LI4), Zusanli(ST36). |
|  | 2c) Depth of insertion, based on a specified unit of measurement, or on a particular tissue level |
|  | Regular needling depth |
|  | 2d) Response sought (e.g., de qi or muscle twitch response) |
|  | De qi |
|  | 2e) Needle stimulation (e.g., manual, electrical) |
|  | Manual |
|  | 2f) Needle retention time |
|  | 30 minutes |
|  | 2g) Needle type (diameter, length, and manufacturer or material) |
|  | Huatuo brand disposable sterile acupuncture needles from Suzhou Medical Supplies Company |
| **3. Treatment regimen** | 3a) Number of treatment sessions |
|  | 14 sessions |
|  | 3b) Frequency and duration of treatment sessions |
|  | Once every other one day, 4 weeks |
| **4. Other components**  **of treatment** | 4a) Details of other interventions administered to the acupuncture group (e.g., moxibustion, cupping, herbs, exercises, lifestyle advice) |
|  | No report |
|  | 4b) Setting and context of treatment, including instructions to practitioners, and information and explanations to patients |
|  | No report |
| **5. Practitioner**  **background** | 5) Description of participating acupuncturists (qualification or professional affiliation, years in acupuncture practice, other relevant experience) |
|  | No report |
| **6. Control or comparator**  **interventions** | 6a) Rationale for the control or comparator in the context of the research question, with sources that justify this choice |
|  | No report |
|  | 6b) Precise description of the control or comparator. If sham acupuncture or any other type of acupuncture-like control is used, provide details as for Items 1 to 3 above. |
|  | Acetaminophen, 0.3g, 1-2 times a day |

**Jeon.2019**

| **Item** | **Detail** |
| --- | --- |
| **1. Acupuncture rationale** | 1a) Style of acupuncture |
|  | Korean |
|  | 1b) Reasoning for treatment provided, based on historical context, literature sources, and/or consensus methods, with references where appropriate |
|  | World Health Organization Regional Office for the Western Pacific. WHO Standard Acupuncture Point Locations in the Western Pacific Region. 1st ed. Manila: World Health Organization. 2008:110-24. |
|  | 1c) Extent to which treatment was varied |
|  | All patients receiving the same treatment at all sessions |
| **2. Details of needling** | 2a) Number of needle insertions per subject per session (mean and range where relevant) |
|  | 4 |
|  | 2b) Names (or location if no standard name) of points used (uni/bilateral) |
|  | Wangu (GB12), Fengchi (GB20) |
|  | 2c) Depth of insertion, based on a specified unit of measurement, or on a particular tissue level |
|  | No report |
|  | 2d) Response sought (e.g., de qi or muscle twitch response) |
|  | No report |
|  | 2e) Needle stimulation (e.g., manual, electrical) |
|  | Electrical |
|  | 2f) Needle retention time |
|  | 20 minutes |
|  | 2g) Needle type (diameter, length, and manufacturer or material) |
|  | No report |
| **3. Treatment regimen** | 3a) Number of treatment sessions |
|  | 3 sessions |
|  | 3b) Frequency and duration of treatment sessions |
|  | Three times a week, 1 week |
| **4. Other components**  **of treatment** | 4a) Details of other interventions administered to the acupuncture group (e.g., moxibustion, cupping, herbs, exercises, lifestyle advice) |
|  | No report |
|  | 4b) Setting and context of treatment, including instructions to practitioners, and information and explanations to patients |
|  | No report |
| **5. Practitioner**  **background** | 5) Description of participating acupuncturists (qualification or professional affiliation, years in acupuncture practice, other relevant experience) |
|  | Conducted by a Korean physician with more than 3 years of clinical experience |
| **6. Control or comparator**  **interventions** | 6a) Rationale for the control or comparator in the context of the research question, with sources that justify this choice |
|  | Symmetrically pierce the non-meridian acupoints and were connected to electro-acupuncture, but no actual electrical stimulation was performed |
|  | 6b) Precise description of the control or comparator. If sham acupuncture or any other type of acupuncture-like control is used, provide details as for Items 1 to 3 above. |
|  | Sham acupuncture, the frequency, sessions and duration were same as intervention group |

**Karst.2001**

| **Item** | **Detail** |
| --- | --- |
| **1. Acupuncture rationale** | 1a) Style of acupuncture |
|  | No report |
|  | 1b) Reasoning for treatment provided, based on historical context, literature sources, and/or consensus methods, with references where appropriate |
|  | No report |
|  | 1c) Extent to which treatment was varied |
|  | Use of a fixed set of points to be combined with a set of points to be used flexibly |
| **2. Details of needling** | 2a) Number of needle insertions per subject per session (mean and range where relevant) |
|  | 6-10 |
|  | 2b) Names (or location if no standard name) of points used (uni/bilateral) |
|  | Bilateral: Fengchi (GB20), Hegu (LI4), Taichong(LR3), Shuaigu(GB8), Yangbai(GB14), Jianjing(GB21), Zulinqi(GB41), Cuanzhu(BL2), Tianzhu(BL10), Kunlun(BL60), Lieque(LU7), Touwei(ST8), Zusanli(ST36), Neiting (ST44), Baihui(DU20). |
|  | 2c) Depth of insertion, based on a specified unit of measurement, or on a particular tissue level |
|  | No report |
|  | 2d) Response sought (e.g., de qi or muscle twitch response) |
|  | No report |
|  | 2e) Needle stimulation (e.g., manual, electrical) |
|  | Manual |
|  | 2f) Needle retention time |
|  | 30 minutes |
|  | 2g) Needle type (diameter, length, and manufacturer or material) |
|  | Seirin B-type needles no.8 (0.3*0.3 mm) and no.3 (0.2*0.15 mm) |
| **3. Treatment regimen** | 3a) Number of treatment sessions |
|  | 10 sessions |
|  | 3b) Frequency and duration of treatment sessions |
|  | Twice a day, 5 weeks |
| **4. Other components**  **of treatment** | 4a) Details of other interventions administered to the acupuncture group (e.g., moxibustion, cupping, herbs, exercises, lifestyle advice) |
|  | No report |
|  | 4b) Setting and context of treatment, including instructions to practitioners, and information and explanations to patients |
|  | No report |
| **5. Practitioner**  **background** | 5) Description of participating acupuncturists (qualification or professional affiliation, years in acupuncture practice, other relevant experience) |
|  | No report |
| **6. Control or comparator**  **interventions** | 6a) Rationale for the control or comparator in the context of the research question, with sources that justify this choice |
|  | The tip of the needle is blunt in order to cause a pricking sensation without actually puncturing the skin. The needle was inserted through a cube-shaped elastic foam to obscure the patients' vision on the insertion point. |
|  | 6b) Precise description of the control or comparator. If sham acupuncture or any other type of acupuncture-like control is used, provide details as for Items 1 to 3 above. |
|  | Sham acupuncture, the frequency, sessions and duration were same as intervention group |

**Koran.2021**

| **Item** | **Detail** |
| --- | --- |
| **1. Acupuncture rationale** | 1a) Style of acupuncture |
|  | Korea |
|  | 1b) Reasoning for treatment provided, based on historical context, literature sources, and/or consensus methods, with references where appropriate |
|  | Previous research, literature and experienced researcher agreement |
|  | 1c) Extent to which treatment was varied |
|  | Semi-standardized predetermined groups |
| **2. Details of needling** | 2a) Number of needle insertions per subject per session (mean and range where relevant) |
|  | 2 |
|  | 2b) Names (or location if no standard name) of points used (uni/bilateral) |
|  | Bilateral: Taichong(LR3), Taiyuan(LU9), Taixi(KI3), Taibai(SP3), Hegu (LI4), Zusanli(ST36), Quchi(LI11) |
|  | 2c) Depth of insertion, based on a specified unit of measurement, or on a particular tissue level |
|  | 5mm~3cm varies depending on location |
|  | 2d) Response sought (e.g., de qi or muscle twitch response) |
|  | De qi |
|  | 2e) Needle stimulation (e.g., manual, electrical) |
|  | Manual |
|  | 2f) Needle retention time |
|  | 20 minutes |
|  | 2g) Needle type (diameter, length, and manufacturer or material) |
|  | 0.25 * 40mm acupuncture needle |
| **3. Treatment regimen** | 3a) Number of treatment sessions |
|  | 8 sessions |
|  | 3b) Frequency and duration of treatment sessions |
|  | Three times a day, 4 weeks |
| **4. Other components**  **of treatment** | 4a) Details of other interventions administered to the acupuncture group (e.g., moxibustion, cupping, herbs, exercises, lifestyle advice) |
|  | Drugs not related to headache are allowed |
|  | 4b) Setting and context of treatment, including instructions to practitioners, and information and explanations to patients |
|  | Patients were informed that they would be randomly directed to any of the three groups, and they were informed that they could leave at any stage of the treatment they voluntarily attend. |
| **5. Practitioner**  **background** | 5) Description of participating acupuncturists (qualification or professional affiliation, years in acupuncture practice, other relevant experience) |
|  | Korean medical doctors with more than 8 years of experience in musculoskeletal disorders and acupuncture. |
| **6. Control or comparator**  **interventions** | 6a) Rationale for the control or comparator in the context of the research question, with sources that justify this choice |
|  | In placebo acupuncture group, ear pin was applied to a parallel point 2 cm from the Korean acupuncture points determined according to the TCI survey. In non-intrusive control group, only traditional pharmacological medical treatment (antidepressant + analgesic treatment was given during the trial period. |
|  | 6b) Precise description of the control or comparator. If sham acupuncture or any other type of acupuncture-like control is used, provide details as for Items 1 to 3 above. |
|  | Patients will be convinced that Korean acupuncture has been applied, the TCI Form has been filled in and sessions have been set as 2 sessions per week as in the Korean acupuncture group. The traditional pharmacological treatments (antidepressant + if necessary analgesic treatment (paracetamol, dexketoprofen trometamol ... etc)) the patients took continued to be given them |

**Kwak.2008**

| **Item** | **Detail** |
| --- | --- |
| **1. Acupuncture rationale** | 1a) Style of acupuncture |
|  | Korean |
|  | 1b) Reasoning for treatment provided, based on historical context, literature sources, and/or consensus methods, with references where appropriate |
|  | No report |
|  | 1c) Extent to which treatment was varied |
|  | Fully individualized treatment protocols within which each patient receives a unique and evolving diagnosis and treatment |
| **2. Details of needling** | 2a) Number of needle insertions per subject per session (mean and range where relevant) |
|  | No report |
|  | 2b) Names (or location if no standard name) of points used (uni/bilateral) |
|  | No report |
|  | 2c) Depth of insertion, based on a specified unit of measurement, or on a particular tissue level |
|  | No report |
|  | 2d) Response sought (e.g., de qi or muscle twitch response) |
|  | No report |
|  | 2e) Needle stimulation (e.g., manual, electrical) |
|  | Manual |
|  | 2f) Needle retention time |
|  | 25 minutes |
|  | 2g) Needle type (diameter, length, and manufacturer or material) |
|  | No report |
| **3. Treatment regimen** | 3a) Number of treatment sessions |
|  | 8 sessions |
|  | 3b) Frequency and duration of treatment sessions |
|  | Twice a week, 4 weeks |
| **4. Other components**  **of treatment** | 4a) Details of other interventions administered to the acupuncture group (e.g., moxibustion, cupping, herbs, exercises, lifestyle advice) |
|  | No report |
|  | 4b) Setting and context of treatment, including instructions to practitioners, and information and explanations to patients |
|  | No report |
| **5. Practitioner**  **background** | 5) Description of participating acupuncturists (qualification or professional affiliation, years in acupuncture practice, other relevant experience) |
|  | No report |
| **6. Control or comparator**  **interventions** | 6a) Rationale for the control or comparator in the context of the research question, with sources that justify this choice |
|  | Choose the non-meridian acupoints |
|  | 6b) Precise description of the control or comparator. If sham acupuncture or any other type of acupuncture-like control is used, provide details as for Items 1 to 3 above. |
|  | Sham acupuncture, the frequency, sessions and duration were same as intervention group |

**Liu.2020**

| **Item** | **Detail** |
| --- | --- |
| **1. Acupuncture rationale** | 1a) Style of acupuncture |
|  | Traditional Chinese Medicine |
|  | 1b) Reasoning for treatment provided, based on historical context, literature sources, and/or consensus methods, with references where appropriate |
|  | Referring to the 2006 edition of the national standard "Name and Positioning of Acupoints" (GB/T12346-2006) |
|  | 1c) Extent to which treatment was varied |
|  | All patients receiving the same treatment at all sessions |
| **2. Details of needling** | 2a) Number of needle insertions per subject per session (mean and range where relevant) |
|  | 24 |
|  | 2b) Names (or location if no standard name) of points used (uni/bilateral) |
|  | (EX-B2)2-4, Fengchi(GB20), Neiguan (PC6), Gongsun (SP4), Lieque(LU7), Zhaohai (KI6), Waiguan(SJ5), Zulinqi(GB41), Shenmai (BL62), Houxi(SI3). |
|  | 2c) Depth of insertion, based on a specified unit of measurement, or on a particular tissue level |
|  | 0.8-1.2 cun: (EX-B2)2-4, Fengchi(GB20), Lieque(LU7); The insersion depth of rest acupoints can be operated flexibly according to the patient's tolerance and fatness |
|  | 2d) Response sought (e.g., de qi or muscle twitch response) |
|  | De qi |
|  | 2e) Needle stimulation (e.g., manual, electrical) |
|  | Manual |
|  | 2f) Needle retention time |
|  | 30 minutes |
|  | 2g) Needle type (diameter, length, and manufacturer or material) |
|  | Huatuo brand 0.30mm×40mm (1.5 cun) stainless steel needles (produced by Suzhou Medical Products Factory Co., Ltd.) |
| **3. Treatment regimen** | 3a) Number of treatment sessions |
|  | 20 sessions |
|  | 3b) Frequency and duration of treatment sessions |
|  | Five times a week, 4 weeks |
| **4. Other components**  **of treatment** | 4a) Details of other interventions administered to the acupuncture group (e.g., moxibustion, cupping, herbs, exercises, lifestyle advice) |
|  | No report |
|  | 4b) Setting and context of treatment, including instructions to practitioners, and information and explanations to patients |
|  | No report |
| **5. Practitioner**  **background** | 5) Description of participating acupuncturists (qualification or professional affiliation, years in acupuncture practice, other relevant experience) |
|  | No report |
| **6. Control or comparator**  **interventions** | 6a) Rationale for the control or comparator in the context of the research question, with sources that justify this choice |
|  | No report |
|  | 6b) Precise description of the control or comparator. If sham acupuncture or any other type of acupuncture-like control is used, provide details as for Items 1 to 3 above. |
|  | Escitalopram oxalate,10mg, qd, 4 weeks. |

**Melchart.2005**

| **Item** | **Detail** |
| --- | --- |
| **1. Acupuncture rationale** | 1a) Style of acupuncture |
|  | Traditional Chinese Medicine |
|  | 1b) Reasoning for treatment provided, based on historical context, literature sources, and/or consensus methods, with references where appropriate |
|  | We developed the study interventions in a consensus process with German acupuncture experts and societies |
|  | 1c) Extent to which treatment was varied |
|  | Use of a fixed set of points to be combined with a set of points to be used flexibly. |
| **2. Details of needling** | 2a) Number of needle insertions per subject per session (mean and range where relevant) |
|  | The total number of needles was limited to 25 per sessions. |
|  | 2b) Names (or location if no standard name) of points used (uni/bilateral) |
|  | Bilateral: Fengchi (GB20), Jianjing(GB21), Taichong(LR3) + optional points. |
|  | 2c) Depth of insertion, based on a specified unit of measurement, or on a particular tissue level |
|  | No report |
|  | 2d) Response sought (e.g., de qi or muscle twitch response) |
|  | De qi |
|  | 2e) Needle stimulation (e.g., manual, electrical) |
|  | Manual |
|  | 2f) Needle retention time |
|  | 30 minutes |
|  | 2g) Needle type (diameter, length, and manufacturer or material) |
|  | No report |
| **3. Treatment regimen** | 3a) Number of treatment sessions |
|  | 12 sessions |
|  | 3b) Frequency and duration of treatment sessions |
|  | Two sessions in each of the first four weeks, followed by one session a week in the remaining four weeks; 8 weeks |
| **4. Other components**  **of treatment** | 4a) Details of other interventions administered to the acupuncture group (e.g., moxibustion, cupping, herbs, exercises, lifestyle advice) |
|  | All patients were allowed to treat acute headaches as needed. Treatment was supposed to follow current guidelines5 and had to be documented in the headache diary. |
|  | 4b) Setting and context of treatment, including instructions to practitioners, and information and explanations to patients |
|  | Patients were informed with respect to acupuncture and minimal acupuncture as follows: “In this study, different types of acupuncture will be compared. One type is similar to the acupuncture treatment used in China. The other type does not follow the principles of traditional Chinese medicine, but has also been associated with positive outcomes in clinical studies.” |
| **5. Practitioner**  **background** | 5) Description of participating acupuncturists (qualification or professional affiliation, years in acupuncture practice, other relevant experience) |
|  | Physicians trained (at least 140 hours, median 500 hours) and experienced (median 10 years) in acupuncture delivered the interventions |
| **6. Control or comparator**  **interventions** | 6a) Rationale for the control or comparator in the context of the research question, with sources that justify this choice |
|  | In each session, physicians needled at least five out of 10 predefined distant non-acupuncture points bilaterally (at least 10 needles) and superficially using fine needles. Physicians avoided “de qi” and manual stimulation of the needles. |
|  | 6b) Precise description of the control or comparator. If sham acupuncture or any other type of acupuncture-like control is used, provide details as for Items 1 to 3 above. |
|  | The number, length, and frequency of the sessions in the minimal acupuncture group were the same as for the acupuncture group. |

**Nie.2019**

| **Item** | **Detail** |
| --- | --- |
| **1. Acupuncture rationale** | 1a) Style of acupuncture |
|  | Traditional Chinese Medicine |
|  | 1b) Reasoning for treatment provided, based on historical context, literature sources, and/or consensus methods, with references where appropriate |
|  | Fernandez-de-Las-Penas C,Ge HY,Alonso-Blanco C,GonzalezIglesias J, Arendt-Nielsen L.Referred pain areas of active myofascial trigger points in head,neck,and shoulder muscles, in chronic tension type headache.J Bodyw Mov Ther.2010;14:391-6. |
|  | 1c) Extent to which treatment was varied |
|  | Fully individualized treatment protocols |
| **2. Details of needling** | 2a) Number of needle insertions per subject per session (mean and range where relevant) |
|  | No report |
|  | 2b) Names (or location if no standard name) of points used (uni/bilateral) |
|  | Trigger points |
|  | 2c) Depth of insertion, based on a specified unit of measurement, or on a particular tissue level |
|  | 0.8-1cun |
|  | 2d) Response sought (e.g., de qi or muscle twitch response) |
|  | De qi |
|  | 2e) Needle stimulation (e.g., manual, electrical) |
|  | Manual |
|  | 2f) Needle retention time |
|  | 20 minutes |
|  | 2g) Needle type (diameter, length, and manufacturer or material) |
|  | No report |
| **3. Treatment regimen** | 3a) Number of treatment sessions |
|  | 12 sessions |
|  | 3b) Frequency and duration of treatment sessions |
|  | Three times a week; 4 weeks |
| **4. Other components**  **of treatment** | 4a) Details of other interventions administered to the acupuncture group (e.g., moxibustion, cupping, herbs, exercises, lifestyle advice) |
|  | No report |
|  | 4b) Setting and context of treatment, including instructions to practitioners, and information and explanations to patients |
|  | No report |
| **5. Practitioner**  **background** | 5) Description of participating acupuncturists (qualification or professional affiliation, years in acupuncture practice, other relevant experience) |
|  | No report |
| **6. Control or comparator**  **interventions** | 6a) Rationale for the control or comparator in the context of the research question, with sources that justify this choice |
|  | No report |
|  | 6b) Precise description of the control or comparator. If sham acupuncture or any other type of acupuncture-like control is used, provide details as for Items 1 to 3 above. |
|  | Eperisone hydrochloride, 50mg, three times a day, four weeks. |

**Söderberg.2006**

| **Item** | **Detail** |
| --- | --- |
| **1. Acupuncture rationale** | 1a) Style of acupuncture |
|  | No report |
|  | 1b) Reasoning for treatment provided, based on historical context, literature sources, and/or consensus methods, with references where appropriate. |
|  | The recommended and clinically most used acupuncture points for CTTH were chosen for the area of the pain, segmental points. |
|  | 1c) Extent to which treatment was varied |
|  | Use of a fixed set of points to be combined with a set of points to be used flexibly |
| **2. Details of needling** | 2a) Number of needle insertions per subject per session (mean and range where relevant) |
|  | In total 10–12 needles, of which not more than eight were distal, peripheral needles in the hands and legs |
|  | 2b) Names (or location if no standard name) of points used (uni/bilateral) |
|  | Fengchi (GB20), Yangbai (GB 14), Hegu (LI4), Neiting(ST44) + optional points: Neiguan (PC6), Daling(PC7), Sanyinjiao(SP6), Yanglinquan(GB34), Touwei(ST8), Dangyang(EX-HN2), Sishencong (EX-HN1). |
|  | 2c) Depth of insertion, based on a specified unit of measurement, or on a particular tissue level |
|  | The needles were inserted to a depth of 2–5 mm or 10–30 mm, depending on location |
|  | 2d) Response sought (e.g., de qi or muscle twitch response) |
|  | De qi |
|  | 2e) Needle stimulation (e.g., manual, electrical) |
|  | Manual |
|  | 2f) Needle retention time |
|  | 30 minutes |
|  | 2g) Needle type (diameter, length, and manufacturer or material) |
|  | Disposable needles (Huanchou, Medema, Stockholm, Sweden) with a dimension of 15 * 0.25 mm and 30 or 40 * 0.30 mm were used. The needle length was dependent on the location of the acupuncture point |
| **3. Treatment regimen** | 3a) Number of treatment sessions |
|  | 10-12 sessions |
|  | 3b) Frequency and duration of treatment sessions |
|  | Once a week, 10-12 weeks |
| **4. Other components**  **of treatment** | 4a) Details of other interventions administered to the acupuncture group (e.g., moxibustion, cupping, herbs, exercises, lifestyle advice) |
|  | No report |
|  | 4b) Setting and context of treatment, including instructions to practitioners, and information and explanations to patients |
|  | No report |
| **5. Practitioner**  **background** | 5) Description of participating acupuncturists (qualification or professional affiliation, years in acupuncture practice, other relevant experience) |
|  | Acupuncture was given mainly by five registered physiotherapists at the three different clinics. They used the same technique and all had long experience in treating patients with acupuncture. |
| **6. Control or comparator**  **interventions** | 6a) Rationale for the control or comparator in the context of the research question, with sources that justify this choice |
|  | In 6b |
|  | 6b) Precise description of the control or comparator. If sham acupuncture or any other type of acupuncture-like control is used, provide details as for Items 1 to 3 above. |
|  | In the physical training group, training was performed by five, mainly four, registered physiotherapists working at three different clinics. The patients performed either two 45-min training sessions a week at the clinic for 5 weeks and then a home-training programme three times a week for 5 weeks (a total of 25 training sessions) or one training session at the clinic and a home training programme once or twice a week for 10 weeks (a total of 25 training sessions). In the relaxation group, relaxation was performed mainly by three registered physiotherapists working in three different clinics. The physiotherapists each had long experience and documented skills for treating patients with relaxation training. Eight to 10 sessions of relaxation training were performed individually under the supervision of a physiotherapist once a week. |

**Schiller.2021**

| **Item** | **Detail** |
| --- | --- |
| **1. Acupuncture rationale** | 1a) Style of acupuncture |
|  | Traditional Chinese Medicine |
|  | 1b) Reasoning for treatment provided, based on historical context, literature sources, and/or consensus methods, with references where appropriate |
|  | Acupuncture was performed as a semi-standardized treatment concept based on the principles of traditional Chinese medicine (TCM), the scientific literature and the opinion of international experts. |
|  | 1c) Extent to which treatment was varied |
|  | Use of a fixed set of points to be combined with a set of points to be used flexibly |
| **2. Details of needling** | 2a) Number of needle insertions per subject per session (mean and range where relevant) |
|  | 11-16 |
|  | 2b) Names (or location if no standard name) of points used (uni/bilateral) |
|  | Baihui (GV20), Taiyang (EX-HN5), Fengchi (GB20), Hegu (LI4), Neiting (ST44), Yintang (GV29), Zulinqi (GB41), Waiguan (SJ5), Kunlun (BL60), Houxi (SI3), Taichong (LR3), Neiguan (PC6), Sishencong (EX-HN1), “Ashi” points. |
|  | 2c) Depth of insertion, based on a specified unit of measurement, or on a particular tissue level |
|  | 0.5–0.8 cun: Baihui (GV20), Neiting (ST44), Taichong (LR3), Sishencong (EX-HN1)  0.3–0.5 cun: Taiyang (EX-HN5), Yintang (GV29), Zulinqi (GB41)  0.8–1.2 cun: Fengchi (GB20)  0.5–1.0 cun: Hegu (LI4), Waiguan (SJ5), Kunlun (BL60), Houxi (SI3), Neiguan (PC6) |
|  | 2d) Response sought (e.g., de qi or muscle twitch response) |
|  | De qi |
|  | 2e) Needle stimulation (e.g., manual, electrical) |
|  | Manual |
|  | 2f) Needle retention time |
|  | 30 minutes |
|  | 2g) Needle type (diameter, length, and manufacturer or material) |
|  | Acupuncture needles were used (25–40 mm * 0.25–0.3 mm; Suzhou). |
| **3. Treatment regimen** | 3a) Number of treatment sessions |
|  | 12 sessions |
|  | 3b) Frequency and duration of treatment sessions |
|  | 3 times in weeks 1-2, 2 times in weeks 3-4 and 1 times in weeks 5-6; 6 weeks |
| **4. Other components**  **of treatment** | 4a) Details of other interventions administered to the acupuncture group (e.g., moxibustion, cupping, herbs, exercises, lifestyle advice) |
|  | Initially, all study participants were reminded about the risks of frequently taking abortive headache medication, recalling the use of headache medication for no more than 10 days/month. In addition, the use of preventive medication and non-medication options was communicated in accordance with guidelines for the treatment of TTH. Any abortive (e.g., ibuprofen) and prophylactic (e.g., amitriptyline) drug treatment existing according to these guidelines had to be continued and documented as usual. |
|  | 4b) Setting and context of treatment, including instructions to practitioners, and information and explanations to patients |
|  | Usual care group were informed about the disease and their treatment options and did not receive any additional intervention; the treatment of their headaches was to be continued as usual as outlined in “screening and recruitment”. |
| **5. Practitioner**  **background** | 5) Description of participating acupuncturists (qualification or professional affiliation, years in acupuncture practice, other relevant experience) |
|  | The acupuncture was performed by an experienced acupuncturist with more than 5 years of clinical experience. |
| **6. Control or comparator**  **interventions** | 6a) Rationale for the control or comparator in the context of the research question, with sources that justify this choice |
|  | The exercise treatment was generally based on the principles of medical training therapy and includes a combination of strength, endurance, flexibility and coordination training. |
|  | 6b) Precise description of the control or comparator. If sham acupuncture or any other type of acupuncture-like control is used, provide details as for Items 1 to 3 above. |
|  | Each of the 60-min treatment sessions took place in the clinic’s gym in small groups of four participants, was adapted in intensity to the previously prepared treatment plan and personally supervised by an experienced qualified physiotherapist; 6 weeks of treatment with 12 interventions. |

**Tavola.1992**

| **Item** | **Detail** |
| --- | --- |
| **1. Acupuncture rationale** | 1a) Style of acupuncture |
|  | Traditional Chinese Medicine |
|  | 1b) Reasoning for treatment provided, based on historical context, literature sources, and/or consensus methods, with references where appropriate |
|  | Institute de Medicine Traditionnelle Chinoisede Shangai, Traitd’ Acupuncture, Paris, Masson, 1978. |
|  | 1c) Extent to which treatment was varied |
|  | The choice of points was made on an individual basis relative to the evaluation of the patient’s ‘energy’ status according to the criteria of traditional Chinese acupuncture. |
| **2. Details of needling** | 2a) Number of needle insertions per subject per session (mean and range where relevant) |
|  | 6-10 |
|  | 2b) Names (or location if no standard name) of points used (uni/bilateral) |
|  | No report |
|  | 2c) Depth of insertion, based on a specified unit of measurement, or on a particular tissue level |
|  | 10-20 mm |
|  | 2d) Response sought (e.g., de qi or muscle twitch response) |
|  | De qi |
|  | 2e) Needle stimulation (e.g., manual, electrical) |
|  | Manual |
|  | 2f) Needle retention time |
|  | 20 minutes |
|  | 2g) Needle type (diameter, length, and manufacturer or material) |
|  | 0.3 mm in diameter |
| **3. Treatment regimen** | 3a) Number of treatment sessions |
|  | 8 sessions |
|  | 3b) Frequency and duration of treatment sessions |
|  | Once a week, 8 weeks |
| **4. Other components**  **of treatment** | 4a) Details of other interventions administered to the acupuncture group (e.g., moxibustion, cupping, herbs, exercises, lifestyle advice) |
|  | No report |
|  | 4b) Setting and context of treatment, including instructions to practitioners, and information and explanations to patients |
|  | No report |
| **5. Practitioner**  **background** | 5) Description of participating acupuncturists (qualification or professional affiliation, years in acupuncture practice, other relevant experience) |
|  | No report |
| **6. Control or comparator**  **interventions** | 6a) Rationale for the control or comparator in the context of the research question, with sources that justify this choice |
|  | For placebo, the same number of needles was used, inserted at a profundity of 2-4 mm in the same regions used in actual acupuncture, but in areas without acupuncture points. The actual and false points were localized both anatomically and with a detector to determine the fall of the cutaneous electrical resistance which is characteristic of the acupuncture point. |
|  | 6b) Precise description of the control or comparator. If sham acupuncture or any other type of acupuncture-like control is used, provide details as for Items 1 to 3 above. |
|  | Sham acupuncture, the frequency, sessions and duration were same as intervention group |

**Wang.2022**

| **Item** | **Detail** |
| --- | --- |
| **1. Acupuncture rationale** | 1a) Style of acupuncture |
|  | Traditional Chinese Medicine |
|  | 1b) Reasoning for treatment provided, based on historical context, literature sources, and/or consensus methods, with references where appropriate |
|  | Flat needle punching method |
|  | 1c) Extent to which treatment was varied |
|  | The choice of points was made on an individual basis relative to the evaluation of the patient’s status. |
| **2. Details of needling** | 2a) Number of needle insertions per subject per session (mean and range where relevant) |
|  | No report |
|  | 2b) Names (or location if no standard name) of points used (uni/bilateral) |
|  | Shenting(DU24), Meichong (BL3), Toulinqi (GB15), Touwei (ST8), Baihui (GV20), Hanyan(GB4),  Naokong (GB19), Naohu (GV17) |
|  | 2c) Depth of insertion, based on a specified unit of measurement, or on a particular tissue level |
|  | 1 cun |
|  | 2d) Response sought (e.g., de qi or muscle twitch response) |
|  | No report |
|  | 2e) Needle stimulation (e.g., manual, electrical) |
|  | Manual |
|  | 2f) Needle retention time |
|  | 4-6 hours |
|  | 2g) Needle type (diameter, length, and manufacturer or material) |
|  | 25×0.25 mm needle |
| **3. Treatment regimen** | 3a) Number of treatment sessions |
|  | 9 sessions |
|  | 3b) Frequency and duration of treatment sessions |
|  | Three times a week, 3 weeks |
| **4. Other components**  **of treatment** | 4a) Details of other interventions administered to the acupuncture group (e.g., moxibustion, cupping, herbs, exercises, lifestyle advice) |
|  | No report |
|  | 4b) Setting and context of treatment, including instructions to practitioners, and information and explanations to patients |
|  | No report |
| **5. Practitioner**  **background** | 5) Description of participating acupuncturists (qualification or professional affiliation, years in acupuncture practice, other relevant experience) |
|  | No report |
| **6. Control or comparator**  **interventions** | 6a) Rationale for the control or comparator in the context of the research question, with sources that justify this choice |
|  | No report |
|  | 6b) Precise description of the control or comparator. If sham acupuncture or any other type of acupuncture-like control is used, provide details as for Items 1 to 3 above. |
|  | Eperisone Hydrochloride, 50mg, tid, 3 weeks. |

**White.2000**

| **Item** | **Detail** |
| --- | --- |
| **1. Acupuncture rationale** | 1a) Style of acupuncture |
|  | No report |
|  | 1b) Reasoning for treatment provided, based on historical context, literature sources, and/or consensus methods, with references where appropriate |
|  | This method is similar to that used in previous studies, but without needle retention. |
|  | 1c) Extent to which treatment was varied |
|  | Use of a fixed set of points to be combined with a set of points to be used flexibly |
| **2. Details of needling** | 2a) Number of needle insertions per subject per session (mean and range where relevant) |
|  | No report |
|  | 2b) Names (or location if no standard name) of points used (uni/bilateral) |
|  | Fengchi (GB20), Hegu (LI4) + optional points. |
|  | 2c) Depth of insertion, based on a specified unit of measurement, or on a particular tissue level |
|  | No report |
|  | 2d) Response sought (e.g., de qi or muscle twitch response) |
|  | De qi |
|  | 2e) Needle stimulation (e.g., manual, electrical) |
|  | Manual |
|  | 2f) Needle retention time |
|  | After insertion, the needle was manipulated for 15s or until De qi was felt if that was sooner. |
|  | 2g) Needle type (diameter, length, and manufacturer or material) |
|  | 30*0.30 mm needle (AcuMedic) |
| **3. Treatment regimen** | 3a) Number of treatment sessions |
|  | 6 sessions |
|  | 3b) Frequency and duration of treatment sessions |
|  | Once a week, 6 weeks |
| **4. Other components**  **of treatment** | 4a) Details of other interventions administered to the acupuncture group (e.g., moxibustion, cupping, herbs, exercises, lifestyle advice) |
|  | No report |
|  | 4b) Setting and context of treatment, including instructions to practitioners, and information and explanations to patients |
|  | No report |
| **5. Practitioner**  **background** | 5) Description of participating acupuncturists (qualification or professional affiliation, years in acupuncture practice, other relevant experience) |
|  | Acupuncturists were recruited from the accredited members of he British Medical Acupuncture Society. |
| **6. Control or comparator**  **interventions** | 6a) Rationale for the control or comparator in the context of the research question, with sources that justify this choice |
|  | Subjects in Group B received sham treatment by tapping a blunted cocktail-stick in a guide tube against bony prominences in four standard areas (two selected from the occipital protuberances, spines of scapula, vertex of skull and spinous process of 6th cervical vertebra, and two areas at the middle of 2nd metacarpal bone of each hand) and one area chosen individually for that patient. Areas that were avoided in Group B were the site of the pain, tender areas and known acupuncture points. Gentle pressure was applied at each point while rotating the cocktail stick for 15 s, before moving to the next point in sequence. The pilot study had shown that acupuncture-naive patients could not identify this procedure as a sham when performed by a single practitioner. Subsequently, if the subject had not responded, acupuncturists were free to add further sham points to mimic real management. |
|  | 6b) Precise description of the control or comparator. If sham acupuncture or any other type of acupuncture-like control is used, provide details as for Items 1 to 3 above. |
|  | Sham acupuncture, the frequency, sessions and duration were same as intervention group |

**Wu.2016**

| **Item** | **Detail** |
| --- | --- |
| **1. Acupuncture rationale** | 1a) Style of acupuncture |
|  | Traditional Chinese Medicine |
|  | 1b) Reasoning for treatment provided, based on historical context, literature sources, and/or consensus methods, with references where appropriate |
|  | Refer to New Century (Second Edition) "Acupuncture and Moxibustion" published by China Traditional Chinese Medicine Press |
|  | 1c) Extent to which treatment was varied |
|  | All patients receiving the same treatment at all sessions |
| **2. Details of needling** | 2a) Number of needle insertions per subject per session (mean and range where relevant) |
|  | 10 |
|  | 2b) Names (or location if no standard name) of points used (uni/bilateral) |
|  | Sishencong (EX-HN1), Shenting(DU24), Yintang (GV29), Neiguan (PC6), Sanyinjiao(SP6) |
|  | 2c) Depth of insertion, based on a specified unit of measurement, or on a particular tissue level |
|  | 0.5-0.8 cun: Sishencong (EX-HN1), Shenting(DU24), Yintang (GV29)  0.8-1.3 cun: Neiguan (PC6), Sanyinjiao(SP6) |
|  | 2d) Response sought (e.g., de qi or muscle twitch response) |
|  | De qi |
|  | 2e) Needle stimulation (e.g., manual, electrical) |
|  | Manual |
|  | 2f) Needle retention time |
|  | 30 minutes |
|  | 2g) Needle type (diameter, length, and manufacturer or material) |
|  | The needles are Huatuo brand 0.25×25mm (1.0 cun), 0.25×40mm (1.5 cun) disposable sterile needles |
| **3. Treatment regimen** | 3a) Number of treatment sessions |
|  | 10 sessions |
|  | 3b) Frequency and duration of treatment sessions |
|  | Five times a week, 2 weeks |
| **4. Other components**  **of treatment** | 4a) Details of other interventions administered to the acupuncture group (e.g., moxibustion, cupping, herbs, exercises, lifestyle advice) |
|  | No report |
|  | 4b) Setting and context of treatment, including instructions to practitioners, and information and explanations to patients |
|  | No report |
| **5. Practitioner**  **background** | 5) Description of participating acupuncturists (qualification or professional affiliation, years in acupuncture practice, other relevant experience) |
|  | No report |
| **6. Control or comparator**  **interventions** | 6a) Rationale for the control or comparator in the context of the research question, with sources that justify this choice |
|  | No report |
|  | 6b) Precise description of the control or comparator. If sham acupuncture or any other type of acupuncture-like control is used, provide details as for Items 1 to 3 above. |
|  | Eperisone Hydrochloride Tablets, 50mg, three times a week, 2 weeks |

**Wang.2018**

| **Item** | **Detail** |
| --- | --- |
| **1. Acupuncture rationale** | 1a) Style of acupuncture |
|  | Traditional Chinese Medicine |
|  | 1b) Reasoning for treatment provided, based on historical context, literature sources, and/or consensus methods, with references where appropriate |
|  | No report |
|  | 1c) Extent to which treatment was varied |
|  | All patients receiving the same treatment at all sessions |
| **2. Details of needling** | 2a) Number of needle insertions per subject per session (mean and range where relevant) |
|  | 10 |
|  | 2b) Names (or location if no standard name) of points used (uni/bilateral) |
|  | Shangwan(RN13), Zhongwan(RN12, Xiawan(RN10), Tianshu (ST25), Qihai(CV5), Neiguan (PC6), Zusanli(ST36). |
|  | 2c) Depth of insertion, based on a specified unit of measurement, or on a particular tissue level |
|  | 1.2-1.5 cun: Shangwan(RN13), Zhongwan(RN12, Xiawan(RN10), Qihai(CV5)  1.5-1.8 cun: Tianshu (ST25)  1.0-1.2 cun: Zusanli(ST36). |
|  | 2d) Response sought (e.g., de qi or muscle twitch response) |
|  | De qi |
|  | 2e) Needle stimulation (e.g., manual, electrical) |
|  | Manual |
|  | 2f) Needle retention time |
|  | 30-45 minutes |
|  | 2g) Needle type (diameter, length, and manufacturer or material) |
|  | No report |
| **3. Treatment regimen** | 3a) Number of treatment sessions |
|  | 18 sessions |
|  | 3b) Frequency and duration of treatment sessions |
|  | Three times a week, 7 weeks |
| **4. Other components**  **of treatment** | 4a) Details of other interventions administered to the acupuncture group (e.g., moxibustion, cupping, herbs, exercises, lifestyle advice) |
|  | No report |
|  | 4b) Setting and context of treatment, including instructions to practitioners, and information and explanations to patients |
|  | No report |
| **5. Practitioner**  **background** | 5) Description of participating acupuncturists (qualification or professional affiliation, years in acupuncture practice, other relevant experience) |
|  | No report |
| **6. Control or comparator**  **interventions** | 6a) Rationale for the control or comparator in the context of the research question, with sources that justify this choice |
|  | No report |
|  | 6b) Precise description of the control or comparator. If sham acupuncture or any other type of acupuncture-like control is used, provide details as for Items 1 to 3 above. |
|  | Eperisone Hydrochloride Tablets, 0.05g, three times a day, 7 weeks |

**White.1996**

| **Item** | **Detail** |
| --- | --- |
| **1. Acupuncture rationale** | 1a) Style of acupuncture |
|  | No report |
|  | 1b) Reasoning for treatment provided, based on historical context, literature sources, and/or consensus methods, with references where appropriate |
|  | No report |
|  | 1c) Extent to which treatment was varied |
|  | Use of a fixed set of points to be combined with a set of points to be used flexibly) |
| **2. Details of needling** | 2a) Number of needle insertions per subject per session (mean and range where relevant) |
|  | 2-6 |
|  | 2b) Names (or location if no standard name) of points used (uni/bilateral) |
|  | Fengchi (GB20), Hegu (LI4) + optional points. |
|  | 2c) Depth of insertion, based on a specified unit of measurement, or on a particular tissue level |
|  | No report |
|  | 2d) Response sought (e.g., de qi or muscle twitch response) |
|  | De qi |
|  | 2e) Needle stimulation (e.g., manual, electrical) |
|  | Manual |
|  | 2f) Needle retention time |
|  | No needle retention |
|  | 2g) Needle type (diameter, length, and manufacturer or material) |
|  | Using disposable acupuncture needles (AcuMedic 30 x 0.25) introduced via a guide tube |
| **3. Treatment regimen** | 3a) Number of treatment sessions |
|  | 6 sessions |
|  | 3b) Frequency and duration of treatment sessions |
|  | Once a week, 6weeks |
| **4. Other components**  **of treatment** | 4a) Details of other interventions administered to the acupuncture group (e.g., moxibustion, cupping, herbs, exercises, lifestyle advice) |
|  | No report |
|  | 4b) Setting and context of treatment, including instructions to practitioners, and information and explanations to patients |
|  | No report |
| **5. Practitioner**  **background** | 5) Description of participating acupuncturists (qualification or professional affiliation, years in acupuncture practice, other relevant experience) |
|  | Most treatments were performed by an acupuncturist who had recently attended a basic acupuncture course |
| **6. Control or comparator**  **interventions** | 6a) Rationale for the control or comparator in the context of the research question, with sources that justify this choice |
|  | Subjects in group B, after an identical initial examination for tenderness, received the sham intervention with the plastic guide tube and cocktail stick. The cocktail stick, fitting loosely inside the guide-tube, was tapped on the skin over bone at defined points and rotated with light pressure. Supported by the other hand holding the guide-tube, the stick caused a slight pressure or gentle scratching sensation. |
|  | 6b) Precise description of the control or comparator. If sham acupuncture or any other type of acupuncture-like control is used, provide details as for Items 1 to 3 above. |
|  | Sham acupuncture, the frequency, sessions and duration were same as intervention group |

**Xiang.2015**

| **Item** | **Detail** |
| --- | --- |
| **1. Acupuncture rationale** | 1a) Style of acupuncture |
|  | Traditional Chinese Medicine |
|  | 1b) Reasoning for treatment provided, based on historical context, literature sources, and/or consensus methods, with references where appropriate |
|  | Selecting acupoints according to the standard of "Acupuncture and Moxibustion - Second Edition of the New Century" |
|  | 1c) Extent to which treatment was varied |
|  | All patients receiving the same treatment at all sessions) |
| **2. Details of needling** | 2a) Number of needle insertions per subject per session (mean and range where relevant) |
|  | No report |
|  | 2b) Names (or location if no standard name) of points used (uni/bilateral) |
|  | Fenglong(ST40), Zusanli(ST36), Taiyang (EX-HN5), Touwei(ST8), Baihui (GV20), Fengchi (GB20), Jinjiaji(EX-B2), Shenmen(HT7), Taichong (LR3). |
|  | 2c) Depth of insertion, based on a specified unit of measurement, or on a particular tissue level |
|  | 1-1.5 cun: Fenglong(ST40)  1-2 cun: Zusanli(ST36)  0.3-0.5 cun: Taiyang (EX-HN5), Shenmen(HT7), Jinjiaji(EX-B2)  0.5-1 cun: Baihui (GV20), Taichong (LR3)  0.5-0.8 cun: Touwei(ST8)  0.8-1.2 cun: Fengchi (GB20) |
|  | 2d) Response sought (e.g., de qi or muscle twitch response) |
|  | De qi |
|  | 2e) Needle stimulation (e.g., manual, electrical) |
|  | Manual |
|  | 2f) Needle retention time |
|  | 30 minutes |
|  | 2g) Needle type (diameter, length, and manufacturer or material) |
|  | Huatuo brand acupuncture needles were selected, with a diameter of 0.35mm and a length of 40nun. |
| **3. Treatment regimen** | 3a) Number of treatment sessions |
|  | 24 sessions |
|  | 3b) Frequency and duration of treatment sessions |
|  | 6 times a week, 4 weeks |
| **4. Other components**  **of treatment** | 4a) Details of other interventions administered to the acupuncture group (e.g., moxibustion, cupping, herbs, exercises, lifestyle advice) |
|  | No report |
|  | 4b) Setting and context of treatment, including instructions to practitioners, and information and explanations to patients |
|  | No report |
| **5. Practitioner**  **background** | 5) Description of participating acupuncturists (qualification or professional affiliation, years in acupuncture practice, other relevant experience) |
|  | No report |
| **6. Control or comparator**  **interventions** | 6a) Rationale for the control or comparator in the context of the research question, with sources that justify this choice |
|  | No report |
|  | 6b) Precise description of the control or comparator. If sham acupuncture or any other type of acupuncture-like control is used, provide details as for Items 1 to 3 above. |
|  | Amitriptyline Hydrochloride Tablets, 25mg, three times a week, 4 weeks |

**Xue.2004**

| **Item** | **Detail** |
| --- | --- |
| **1. Acupuncture rationale** | 1a) Style of acupuncture |
|  | Traditional Chinese Medicine |
|  | 1b) Reasoning for treatment provided, based on historical context, literature sources, and/or consensus methods, with references where appropriate |
|  | A traditional Chinese medicine diagnostic procedure was performed for all patients to guide the sub- grouping and acupuncture point selection |
|  | 1c) Extent to which treatment was varied |
|  | Fully individualized treatment protocols within which each patient receives a unique and evolving diagnosis and treatment |
| **2. Details of needling** | 2a) Number of needle insertions per subject per session (mean and range where relevant) |
|  | No report |
|  | 2b) Names (or location if no standard name) of points used (uni/bilateral) |
|  | Hegu(L14), Waiguan (SJ5), Fenglong(ST40), Kunlun(GB60), Taichong(LR3), Xingjian(LR2), Hegu(LI4), Lieque(LU7), Saninjiao(SP6), Zusanli(ST36) Hegu(LI4), Waiguan(SJ5), Taichong(LR3), Taixi(KI3), Waiguan(SJ5). |
|  | 2c) Depth of insertion, based on a specified unit of measurement, or on a particular tissue level |
|  | No report |
|  | 2d) Response sought (e.g., de qi or muscle twitch response) |
|  | De qi |
|  | 2e) Needle stimulation (e.g., manual, electrical) |
|  | Electrical |
|  | 2f) Needle retention time |
|  | 30 minutes |
|  | 2g) Needle type (diameter, length, and manufacturer or material) |
|  | No report |
| **3. Treatment regimen** | 3a) Number of treatment sessions |
|  | 8 sessions |
|  | 3b) Frequency and duration of treatment sessions |
|  | Twice a week, 4 weeks |
| **4. Other components**  **of treatment** | 4a) Details of other interventions administered to the acupuncture group (e.g., moxibustion, cupping, herbs, exercises, lifestyle advice) |
|  | No report |
|  | 4b) Setting and context of treatment, including instructions to practitioners, and information and explanations to patients |
|  | No report |
| **5. Practitioner**  **background** | 5) Description of participating acupuncturists (qualification or professional affiliation, years in acupuncture practice, other relevant experience) |
|  | No report |
| **6. Control or comparator**  **interventions** | 6a) Rationale for the control or comparator in the context of the research question, with sources that justify this choice |
|  | For sham acupuncture, needles were inserted into points that were 5 to 10 mm away from the correct acupoint lo- cations and maintained at a superficial level of insertion to minimize stimulation. I No further manipulation was performed. The sham acupuncture was continuous low-frequency (0.2 Hz) and low-intensity (0.1 volt) stimulation delivered by a modified MME 501 acupuncture instrument. For both real acupuncture and sham acupuncture, electrical stimulation was monitored by an indicator light throughout the treatment period. |
|  | 6b) Precise description of the control or comparator. If sham acupuncture or any other type of acupuncture-like control is used, provide details as for Items 1 to 3 above. |
|  | Sham acupuncture, the frequency, sessions and duration were same as intervention group |

**Yang.2020**

| **Item** | **Detail** |
| --- | --- |
| **1. Acupuncture rationale** | 1a) Style of acupuncture |
|  | Traditional Chinese Medicine |
|  | 1b) Reasoning for treatment provided, based on historical context, literature sources, and/or consensus methods, with references where appropriate |
|  | No report |
|  | 1c) Extent to which treatment was varied |
|  | All patients receiving the same treatment at all sessions. |
| **2. Details of needling** | 2a) Number of needle insertions per subject per session (mean and range where relevant) |
|  | No report |
|  | 2b) Names (or location if no standard name) of points used (uni/bilateral) |
|  | Neiguan (PC6), Sanyinjiao(SP6), Taichong (LR3), Baihui (GV20), Yintang (GV29)+Trigger points. |
|  | 2c) Depth of insertion, based on a specified unit of measurement, or on a particular tissue level |
|  | 0.5-1 cun: Trigger points, Neiguan (PC6), Taichong (LR3)  0.8-1.2 cun: Sanyinjiao(SP6)  0.5-1 cun: Baihui (GV20)  0.3-0.5 cun: Yintang (GV29) |
|  | 2d) Response sought (e.g., de qi or muscle twitch response) |
|  | No report |
|  | 2e) Needle stimulation (e.g., manual, electrical) |
|  | Manual |
|  | 2f) Needle retention time |
|  | 30 minutes |
|  | 2g) Needle type (diameter, length, and manufacturer or material) |
|  | No report |
| **3. Treatment regimen** | 3a) Number of treatment sessions |
|  | 14 sessions |
|  | 3b) Frequency and duration of treatment sessions |
|  | Once a day, 2 weeks |
| **4. Other components**  **of treatment** | 4a) Details of other interventions administered to the acupuncture group (e.g., moxibustion, cupping, herbs, exercises, lifestyle advice) |
|  | No report |
|  | 4b) Setting and context of treatment, including instructions to practitioners, and information and explanations to patients |
|  | No report |
| **5. Practitioner**  **background** | 5) Description of participating acupuncturists (qualification or professional affiliation, years in acupuncture practice, other relevant experience) |
|  | No report |
| **6. Control or comparator**  **interventions** | 6a) Rationale for the control or comparator in the context of the research question, with sources that justify this choice |
|  | No report |
|  | 6b) Precise description of the control or comparator. If sham acupuncture or any other type of acupuncture-like control is used, provide details as for Items 1 to 3 above. |
|  | Ibuprofen Sustained Release Capsules, 0.3g, twice a day, two weeks. |

**Zhou.2015**

| **Item** | **Detail** |
| --- | --- |
| **1. Acupuncture rationale** | 1a) Style of acupuncture |
|  | Traditional Chinese Medicine |
|  | 1b) Reasoning for treatment provided, based on historical context, literature sources, and/or consensus methods, with references where appropriate |
|  | No report |
|  | 1c) Extent to which treatment was varied |
|  | Use of a fixed set of points to be combined with a set of points to be used flexibly |
| **2. Details of needling** | 2a) Number of needle insertions per subject per session (mean and range where relevant) |
|  | No report |
|  | 2b) Names (or location if no standard name) of points used (uni/bilateral) |
|  | Baihui (GV20), Yintang (GV29), Taiyang (EX-HN5), Hegu (LI4), Taichong (LR3), Neiguan (PC6) + optional points. |
|  | 2c) Depth of insertion, based on a specified unit of measurement, or on a particular tissue level |
|  | 0.5-1 cun: Baihui (GV20), Taiyang (EX-HN5), Hegu (LI4), Neiguan (PC6)  0.3-0.5 cun: Taiyang (EX-HN5) |
|  | 2d) Response sought (e.g., de qi or muscle twitch response) |
|  | De qi |
|  | 2e) Needle stimulation (e.g., manual, electrical) |
|  | Manual |
|  | 2f) Needle retention time |
|  | 30 minutes |
|  | 2g) Needle type (diameter, length, and manufacturer or material) |
|  | Huatuo brand 1.0 cun, 1.5 cun stainless steel needle |
| **3. Treatment regimen** | 3a) Number of treatment sessions |
|  | 20 sessions |
|  | 3b) Frequency and duration of treatment sessions |
|  | 5 times a week, 4 weeks |
| **4. Other components**  **of treatment** | 4a) Details of other interventions administered to the acupuncture group (e.g., moxibustion, cupping, herbs, exercises, lifestyle advice) |
|  | During the trial period, avoiding other medicines for the treatment of TTH. The patients in both groups were instructed to adjust their lifestyles and take appropriate physical exercise. |
|  | 4b) Setting and context of treatment, including instructions to practitioners, and information and explanations to patients |
|  | No report |
| **5. Practitioner**  **background** | 5) Description of participating acupuncturists (qualification or professional affiliation, years in acupuncture practice, other relevant experience) |
|  | No report |
| **6. Control or comparator**  **interventions** | 6a) Rationale for the control or comparator in the context of the research question, with sources that justify this choice |
|  | No report |
|  | 6b) Precise description of the control or comparator. If sham acupuncture or any other type of acupuncture-like control is used, provide details as for Items 1 to 3 above. |
|  | Amitriptyline Hydrochloride Tablets, 25mg, once a day. |

**Zhang.2009**

| **Item** | **Detail** |
| --- | --- |
| **1. Acupuncture rationale** | 1a) Style of acupuncture |
|  | Traditional Chinese Medicine |
|  | 1b) Reasoning for treatment provided, based on historical context, literature sources, and/or consensus methods, with references where appropriate |
|  | No report |
|  | 1c) Extent to which treatment was varied |
|  | Use of a fixed set of points to be combined with a set of points to be used flexibly |
| **2. Details of needling** | 2a) Number of needle insertions per subject per session (mean and range where relevant) |
|  | No report |
|  | 2b) Names (or location if no standard name) of points used (uni/bilateral) |
|  | Baihui (GV20), Shenting(DU24), Touwei(ST8), Taiyang (EX-HN5), Taichong (LR3). |
|  | 2c) Depth of insertion, based on a specified unit of measurement, or on a particular tissue level |
|  | No report |
|  | 2d) Response sought (e.g., de qi or muscle twitch response) |
|  | De qi |
|  | 2e) Needle stimulation (e.g., manual, electrical) |
|  | Manual |
|  | 2f) Needle retention time |
|  | 30 minutes |
|  | 2g) Needle type (diameter, length, and manufacturer or material) |
|  | Diameter 0.35mm, length 25mm; Huatuo brand acupuncture needles. |
| **3. Treatment regimen** | 3a) Number of treatment sessions |
|  | 12 sessions |
|  | 3b) Frequency and duration of treatment sessions |
|  | 3 times a week, 4 weeks |
| **4. Other components**  **of treatment** | 4a) Details of other interventions administered to the acupuncture group (e.g., moxibustion, cupping, herbs, exercises, lifestyle advice) |
|  | No report |
|  | 4b) Setting and context of treatment, including instructions to practitioners, and information and explanations to patients |
|  | No report |
| **5. Practitioner**  **background** | 5) Description of participating acupuncturists (qualification or professional affiliation, years in acupuncture practice, other relevant experience) |
|  | No report |
| **6. Control or comparator**  **interventions** | 6a) Rationale for the control or comparator in the context of the research question, with sources that justify this choice |
|  | No report |
|  | 6b) Precise description of the control or comparator. If sham acupuncture or any other type of acupuncture-like control is used, provide details as for Items 1 to 3 above. |
|  | Sham acupuncture (three times a week) + oral amitriptyline (0.5mg, take once every night); Four weeks. |

**Zhang.2021**

| **Item** | **Detail** |
| --- | --- |
| **1. Acupuncture rationale** | 1a) Style of acupuncture |
|  | Traditional Chinese Medicine |
|  | 1b) Reasoning for treatment provided, based on historical context, literature sources, and/or consensus methods, with references where appropriate |
|  | With reference to the "Criteria for Diagnosis and Efficacy Evaluation of Headache" formulated by the National Encephalopathy Emergency Collaborative Group of the State Administration of Traditional Chinese Medicine |
|  | 1c) Extent to which treatment was varied |
|  | All patients receiving the same treatment at all sessions. |
| **2. Details of needling** | 2a) Number of needle insertions per subject per session (mean and range where relevant) |
|  | No report |
|  | 2b) Names (or location if no standard name) of points used (uni/bilateral) |
|  | Bilateral: Baihui (GV20), Fengchi (GB20), Shuaigu(GB8), Jiaosun(SJ20), Taiyang (EX-HN5), Lieque(LU7), Hegu (LI4), Taichong (LR3), “Ashi”points |
|  | 2c) Depth of insertion, based on a specified unit of measurement, or on a particular tissue level |
|  | 5-10mm: Jiaosun(SJ20), Lieque(LU7), Taiyang (EX-HN5)  13-20mm: Baihui (GV20), Hegu (LI4) Shuaigu(GB8),  20-30mm: Fengchi (GB20)  13-25mm: Taichong (LR3) |
|  | 2d) Response sought (e.g., de qi or muscle twitch response) |
|  | No report |
|  | 2e) Needle stimulation (e.g., manual, electrical) |
|  | Manual |
|  | 2f) Needle retention time |
|  | 30 minutes |
|  | 2g) Needle type (diameter, length, and manufacturer or material) |
|  | Use Huatuo brand stainless steel filigree needles (produced by Suzhou Medical Supplies Factory) of 0.30 mm×25 mm or 0.30 mm×50 mm |
| **3. Treatment regimen** | 3a) Number of treatment sessions |
|  | 28 sessions |
|  | 3b) Frequency and duration of treatment sessions |
|  | Once a day, 4 weeks |
| **4. Other components**  **of treatment** | 4a) Details of other interventions administered to the acupuncture group (e.g., moxibustion, cupping, herbs, exercises, lifestyle advice) |
|  | No report |
|  | 4b) Setting and context of treatment, including instructions to practitioners, and information and explanations to patients |
|  | No report |
| **5. Practitioner**  **background** | 5) Description of participating acupuncturists (qualification or professional affiliation, years in acupuncture practice, other relevant experience) |
|  | No report |
| **6. Control or comparator**  **interventions** | 6a) Rationale for the control or comparator in the context of the research question, with sources that justify this choice |
|  | No report |
|  | 6b) Precise description of the control or comparator. If sham acupuncture or any other type of acupuncture-like control is used, provide details as for Items 1 to 3 above. |
|  | Amitriptyline (75mg, once a day, four weeks) |

**Zhu.2013**

| **Item** | **Detail** |
| --- | --- |
| **1. Acupuncture rationale** | 1a) Style of acupuncture |
|  | Traditional Chinese Medicine |
|  | 1b) Reasoning for treatment provided, based on historical context, literature sources, and/or consensus methods, with references where appropriate |
|  | No report |
|  | 1c) Extent to which treatment was varied |
|  | Use of a fixed set of points to be combined with a set of points to be used flexibly |
| **2. Details of needling** | 2a) Number of needle insertions per subject per session (mean and range where relevant) |
|  | No report |
|  | 2b) Names (or location if no standard name) of points used (uni/bilateral) |
|  | Baihui (GV20), Fengchi (GB20), Taiyang (EX-HN5), Touwei(ST8), Taichong (LR3), Geshu(BL18), Xuehai(SP10), Hegu (LI4), “Ashi” points + optional points. |
|  | 2c) Depth of insertion, based on a specified unit of measurement, or on a particular tissue level |
|  | No report |
|  | 2d) Response sought (e.g., de qi or muscle twitch response) |
|  | De qi |
|  | 2e) Needle stimulation (e.g., manual, electrical) |
|  | Manual |
|  | 2f) Needle retention time |
|  | 30 minutes |
|  | 2g) Needle type (diameter, length, and manufacturer or material) |
|  | No report |
| **3. Treatment regimen** | 3a) Number of treatment sessions |
|  | 24 sessions |
|  | 3b) Frequency and duration of treatment sessions |
|  | Six times a week, 4 weeks, |
| **4. Other components**  **of treatment** | 4a) Details of other interventions administered to the acupuncture group (e.g. moxibustion, cupping, herbs, exercises, lifestyle advice) |
|  | No report |
|  | 4b) Setting and context of treatment, including instructions to practitioners, and information and explanations to patients |
|  | No report |
| **5. Practitioner**  **background** | 5) Description of participating acupuncturists (qualification or professional affiliation, years in acupuncture practice, other relevant experience) |
|  | No report |
| **6. Control or comparator**  **interventions** | 6a) Rationale for the control or comparator in the context of the research question, with sources that justify this choice |
|  | No report |
|  | 6b) Precise description of the control or comparator. If sham acupuncture or any other type of acupuncture-like control is used, provide details as for Items 1 to 3 above. |
|  | Amitriptyline (25mg, twice a day) + Oryzanol (30mg, three times a day); Four weeks. |

**Zheng.2022**

| **Item** | **Detail** |
| --- | --- |
| **1. Acupuncture rationale** | 1a) Style of acupuncture |
|  | Traditional Chinese Medicine |
|  | 1b) Reasoning for treatment provided, based on historical context, literature sources, and/or consensus methods, with references where appropriate |
|  | Referring to the 2006 edition of the national standard "Name and Positioning of Acupoints" (GB/T12346-2006) and "Code of Practice for Acupuncture and Moxibustion" (GB/T21709.22-2013) |
|  | 1c) Extent to which treatment was varied |
|  | All patients receiving the same treatment at all sessions |
| **2. Details of needling** | 2a) Number of needle insertions per subject per session (mean and range where relevant) |
|  | Nine |
|  | 2b) Names (or location if no standard name) of points used (uni/bilateral) |
|  | Baihui (GV20), Taiyang (EX-HN5), Fengchi (GB20), Taichong (LR3), Hegu (LI4). |
|  | 2c) Depth of insertion, based on a specified unit of measurement, or on a particular tissue level |
|  | Baihui (GV20) is pierced 0.5-1 cun, Taiyang (EX-HN5) is pierced 0.3-0.5 cun, Fengchi (GB20) is pierced 0.8-1.2 cun, Taichong (LR3), Hegu (LI4) are pierced 0.5-0.8 cun. |
|  | 2d) Response sought (e.g., de qi or muscle twitch response) |
|  | De qi |
|  | 2e) Needle stimulation (e.g., manual, electrical) |
|  | Manual |
|  | 2f) Needle retention time |
|  | 30 minutes |
|  | 2g) Needle type (diameter, length, and manufacturer or material) |
|  | The acupuncture needles used in this study were "Huatuo brand" disposable sterile needles with needle tube, produced by Suzhou Medical Supplies Factory Co., Ltd., with specifications of 0.30×25mm and 0.30×40mm. |
| **3. Treatment regimen** | 3a) Number of treatment sessions |
|  | 20 sessions |
|  | 3b) Frequency and duration of treatment sessions |
|  | Three times a week in 1-4 weeks, twice a week in 5-8 weeks, 8 weeks |
| **4. Other components**  **of treatment** | 4a) Details of other interventions administered to the acupuncture group (e.g., moxibustion, cupping, herbs, exercises, lifestyle advice) |
|  | The participants in both groups were given headache educations in each clinic visits, which included the methods to record headache diaries, avoiding predisposing factors for headaches, and suggestions for the selection of acute drugs |
|  | 4b) Setting and context of treatment, including instructions to practitioners, and information and explanations to patients |
|  | During the study period, subjects were advised to avoid painkiller. However, if the headache is severe and exceeds the patient's tolerance, it is allowed to take acute analgesic drugs in principle, but it is not allowed to take TTH preventive drugs. |
| **5. Practitioner**  **background** | 5) Description of participating acupuncturists (qualification or professional affiliation, years in acupuncture practice, other relevant experience) |
|  | Acupuncturists qualified for the study had at least 5 years of training and had experience in participating in clinical trials. |
| **6. Control or comparator**  **interventions** | 6a) Rationale for the control or comparator in the context of the research question, with sources that justify this choice |
|  | The acupoint location is the same as in the acupuncture group. The tube needle break the skin about 2mm, no needle transmission, try to avoid de qi. |
|  | 6b) Precise description of the control or comparator. If sham acupuncture or any other type of acupuncture-like control is used, provide details as for Items 1 to 3 above. |
|  | Sham acupuncture, the frequency, sessions and duration were same as intervention group |

**5:** **Risk of bias**

**Chassot.2015**

| **Bias** | **Authors' judgement** | **Support for judgement** |
| --- | --- | --- |
| **Randomization process** | Low | The patients were randomized into the two groups (electroacupuncture or sham). We used simple randomization to allocate the interventions, using computer software. The simple randomization relies on independent and equal probabilities to receive each intervention, for each subject. The clinical and demographic characteristics of the subjects according to the sequence allocation were comparable and are shown in Table 1. |
| **Deviations from intended interventions** | Low | The non-adherence to intervention was unrelated to the experimental, and appropriate analysis had been applied, |
| **Missing outcome data** | Low | Twenty-nine patients completed the study, and two participants in the EA first phase discontinued, one participant because of financial problems and the other participants because of personal problems. In the phase receiving sham first, three patients dropped out for different reason. |
| **Measurement of the outcome** | Low | Although the outcome assessor, the care provider, and the patient were blinded (as recommended in the Delphi List for the quality of clinical trials), the attending acupuncturist was not because that was impossible. |
| **Selection of the reported result** | Low | Hospital de Clínicas de Porto Alegre (HCPA) (Institutional Review Board IRB 0000921 - application no.09-259) and was performed in accordance with the Declaration of Helsinki (Resolution 196/96 of the National Health Council). |
| **Overall bias** | Low |  |

**Duan.2013**

| **Bias** | **Authors' judgement** | **Support for judgement** |
| --- | --- | --- |
| **Randomization process** | Some concerns | Patients were randomized with a standard table of random numbers. No conceal of allocation was described. |
| **Deviations from intended interventions** | Some concerns | Acupuncture practitioner and patients may know the assignment. |
| **Missing outcome data** | Some concerns | No information about missing outcome data. |
| **Measurement of the outcome** | Some concerns | No blinding of outcome assessment was described. |
| **Selection of the reported result** | Some concerns | No pre-registration of the trial protocol |
| **Overall bias** | Some concerns |  |

**Deng.2013**

| **Bias** | **Authors' judgement** | **Support for judgement** |
| --- | --- | --- |
| **Randomization process** | Some concerns | Patients were randomized with a standard table of random numbers. No conceal of allocation and baseline comparison was described. |
| **Deviations from intended interventions** | Some concerns | Acupuncture practitioner and patients may know the assignment and without appropriate analysis. |
| **Missing outcome data** | Low | All participants completed the therapeutic program and were analyzed. |
| **Measurement of the outcome** | Some concerns | No blinding of outcome assessment was described. |
| **Selection of the reported result** | Some concerns | No pre-registration of the trial protocol |
| **Overall bias** | Some concerns |  |

**Endres.2007**

| **Bias** | **Authors' judgement** | **Support for judgement** |
| --- | --- | --- |
| **Randomization process** | Low | Randomization was performed centrally by telephone using a secure, password-protected database. Block sizes were of variable length unknown to trial physicians. This ensured full allocation concealment: allocation could not be guessed before a patient was unambiguously registered on trial or altered afterwards. Independent clinical monitors regularly visited all study sites. Baseline demographic and clinical characteristics were similar between groups. |
| **Deviations from intended interventions** | Low | The non-adherence to intervention was unrelated to the experimental, and appropriate analysis had been applied. |
| **Missing outcome data** | Low | Compliance was excellent: only six patients (1 verum, 5 sham) did not receive  planned treatment. |
| **Measurement of the outcome** | Low | Both interviewers and patients were blind to the criterion for additional treatment. |
| **Selection of the reported result** | Low | Provided pre-registration of the trial protocol |
| **Overall bias** | Low |  |

**Guo.2020**

| **Bias** | **Authors' judgement** | **Support for judgement** |
| --- | --- | --- |
| **Randomization process** | Some concerns | Patients were randomized with a standard table of random numbers. No conceal of allocation was described. |
| **Deviations from intended interventions** | Some concerns | Blinding the acupuncture practitioner was impossible due to methodological reasons and without appropriate analysis. |
| **Missing outcome data** | Low | All participants completed the therapeutic program and were analyzed. |
| **Measurement of the outcome** | Some concerns | No blinding of outcome assessment was described. |
| **Selection of the reported result** | Some concerns | No pre-registration of the trial protocol |
| **Overall bias** | Some concerns |  |

**Guo.2019**

| **Bias** | **Authors' judgement** | **Support for judgement** |
| --- | --- | --- |
| **Randomization process** | Low | Patients were randomized with a standard table of random numbers. No conceal of allocation was described. |
| **Deviations from intended interventions** | Some concerns | Blinding the acupuncture practitioner was impossible due to methodological reasons. |
| **Missing outcome data** | Low | Almost all participants completed the therapeutic program and were analyzed. |
| **Measurement of the outcome** | Some concerns | No blinding of outcome assessment was described. |
| **Selection of the reported result** | Some concerns | No pre-registration of the trial protocol |
| **Overall bias** | Some concerns |  |

**Huang.2012**

| **Bias** | **Authors' judgement** | **Support for judgement** |
| --- | --- | --- |
| **Randomization process** | Some concerns | Patients were randomized with a standard table of random numbers. No conceal of allocation was described. |
| **Deviations from intended interventions** | Some concerns | Blinding the acupuncture practitioner was impossible due to methodological reasons and without appropriate analysis. |
| **Missing outcome data** | Low | Almost all participants completed the therapeutic program and were analyzed. |
| **Measurement of the outcome** | Some concerns | No blinding of outcome assessment was described. |
| **Selection of the reported result** | Some concerns | No pre-registration of the trial protocol |
| **Overall bias** | Some concerns |  |

**Jeon.2019**

| **Bias** | **Authors' judgement** | **Support for judgement** |
| --- | --- | --- |
| **Randomization process** | Some concerns | No conceal of allocation was described. |
| **Deviations from intended interventions** | Low | The non-adherence to intervention was unrelated to the experimental, and appropriate analysis had been applied. |
| **Missing outcome data** | Low | Almost all participants completed the therapeutic program and were analyzed. |
| **Measurement of the outcome** | Some concerns | No blinding of outcome assessment was described. |
| **Selection of the reported result** | Some concerns | No pre-registration of the trial protocol |
| **Overall bias** | Some concerns |  |

**Karst.2001**

| **Bias** | **Authors' judgement** | **Support for judgement** |
| --- | --- | --- |
| **Randomization process** | Some concerns | Subjects were randomly assigned to placebo or verum condition in a single-blind, placebo-controlled design. No conceal of allocation was described. Prior to treatment, placebo and verum groups did not differ significantly in terms of VAS, headache frequency, sociodemographic data and quality of life parameters, except for more minimizing and wishful thinking (FQCI, subscale 5) in the placebo group (Wilcoxon-test: Z=x2.125, P=0.034) (Table 1). |
| **Deviations from intended interventions** | Some concerns | Blinding the acupuncture practitioner was impossible due to methodological reasons and without appropriate analysis. |
| **Missing outcome data** | Some concerns | No report about the number of patients who dropped out. |
| **Measurement of the outcome** | Low | Both the investigator performing follow-up examinations and statistical procedures and the patients were blind to treatment condition (placebo vs. verum). |
| **Selection of the reported result** | Some concerns | No pre-registration of the trial protocol |
| **Overall bias** | Some concerns |  |

**Koran.2021**

| **Bias** | **Authors' judgement** | **Support for judgement** |
| --- | --- | --- |
| **Randomization process** | Some concerns | They were informed that they would be randomly directed to any of the three groups, and they were informed that they could leave at any stage of the treatment they voluntarily attend. No report about the concealment of allocation groups. |
| **Deviations from intended interventions** | Some concerns | Blinding the acupuncture practitioner was impossible due to methodological reasons and without appropriate analysis. |
| **Missing outcome data** | Some concerns | No report about the number of patients who dropped out. |
| **Measurement of the outcome** | Some concerns | No report about the blinding of outcome assessor. |
| **Selection of the reported result** | Some concerns | No pre-registration of the trial protocol |
| **Overall bias** | Some concerns |  |

**Kwak.2008**

| **Bias** | **Authors' judgement** | **Support for judgement** |
| --- | --- | --- |
| **Randomization process** | Some concerns | Random allocation was performed immediately before the treatment, and no report about the concealment of allocation groups. |
| **Deviations from intended interventions** | Some concerns | Blinding the acupuncture practitioner was impossible due to methodological reasons and without appropriate analysis. |
| **Missing outcome data** | Some concerns | No report about the number of patients who dropped out. |
| **Measurement of the outcome** | Some concerns | No report about the blinding of outcome assessor. |
| **Selection of the reported result** | Some concerns | No pre-registration of the trial protocol |
| **Overall bias** | Some concerns |  |

**Liu.2020**

| **Bias** | **Authors' judgement** | **Support for judgement** |
| --- | --- | --- |
| **Randomization process** | Some concerns | Random allocation was performed immediately before the treatment, and no description about the concealment of allocation groups. |
| **Deviations from intended interventions** | Some concerns | Blinding the acupuncture practitioner was impossible due to methodological reasons and without appropriate analysis. |
| **Missing outcome data** | Low | Almost all participants completed the therapeutic program and were analyzed. |
| **Measurement of the outcome** | Some concerns | The outcome assessor and the patient were unblinded. |
| **Selection of the reported result** | Some concerns | No pre-registration of the trial protocol |
| **Overall bias** | Some concerns |  |

**Melchart.2005**

| **Bias** | **Authors' judgement** | **Support for judgement** |
| --- | --- | --- |
| **Randomization process** | Low | After a baseline phase of four weeks, we used a centralised telephone randomisation procedure (random list generated with sample size 2.0 by the statistician) to randomise patients, stratified by centre (block size 12 unknown to trial physicians), in a 2:1:1 ratio (acupuncture:minimal acupuncture:waiting list). Groups were comparable at baseline in most respects. |
| **Deviations from intended interventions** | Low | The non-adherence to intervention was unrelated to the experimental, and appropriate analysis had been applied. |
| **Missing outcome data** | Low | Almost all participants completed the therapeutic program and were analyzed. |
| **Measurement of the outcome** | Low | Two blinded evaluators analyzed headache diaries. |
| **Selection of the reported result** | Low | The methods of the trial have been described in detail elsewhere.Trial registration number ISRCTN9737659. |
| **Overall bias** | Low |  |

**Nie.2019**

| **Bias** | **Authors' judgement** | **Support for judgement** |
| --- | --- | --- |
| **Randomization process** | Some concerns | No conceal of allocation was described. |
| **Deviations from intended interventions** | Low | The non-adherence to intervention was unrelated to the experimental, and appropriate analysis had been applied. |
| **Missing outcome data** | Low | Almost all participants completed the therapeutic program and were analyzed. |
| **Measurement of the outcome** | Lows | Blinding of outcome assessment was described. |
| **Selection of the reported result** | Some concerns | No pre-registration of the trial protocol |
| **Overall bias** | Some concerns |  |

**Söderberg.2006**

| **Bias** | **Authors' judgement** | **Support for judgement** |
| --- | --- | --- |
| **Randomization process** | Low | Ninety patients were randomized, using sealed envelopes, into one of three different treatment groups: an acupuncture group (n = 30), a physical training group (n = 30) or a relaxation training group (n = 30). Differences of sex, living arrangements, education, work and headache duration were non-significant between the three treatment groups before treatment. |
| **Deviations from intended interventions** | Low | All participants completed the trial after treatment and appropriate analysis was used |
| **Missing outcome data** | Low | All patients completed the treatment and the first follow-up directly after treatment with collected diaries. |
| **Measurement of the outcome** | Low | The nursing assistant interviewed the subjects in a separate room and remained unaware of their group allocation. |
| **Selection of the reported result** | Some concerns | No pre-registration of the trial protocol |
| **Overall bias** | Some concerns |  |

**Schiller.2020**

| **Bias** | **Authors' judgement** | **Support for judgement** |
| --- | --- | --- |
| **Randomization process** | Low | By means of pre-generated randomisation lists (i.e. group ratio of 1:1:1) with varying permutation block sizes by an independent statistician, all participants were assigned to one of the four intervention groups. The written assignment sheets were handed over to the treating study physicians in opaque sealed envelopes. The assignment was made by the study participant randomly drawing one of the opaque randomization envelopes after inclusion in the study. All data were homogeneously distributed with regard to group differences and did not show significant differences (p > 0.05). |
| **Deviations from intended interventions** | Some concerns | Most withdrawal reasons were not specified. |
| **Missing outcome data** | Low | Two hundred and twelve persons were screened, 96 were randomised to one of the four groups. Overall, 16 participants were excluded or withdrew consent during the course of the study. |
| **Measurement of the outcome** | Some concerns | No blinding of outcome assessment was described. |
| **Selection of the reported result** | Low | Reported number of the trial protocol |
| **Overall bias** | Some concerns |  |

**Tavola.1992**

| **Bias** | **Authors' judgement** | **Support for judgement** |
| --- | --- | --- |
| **Randomization process** | Some concerns | After the diagnostic interview for admission and after 1 month of baseline observation, the patients were randomly and blindly assigned to two subject groups, one treated with Chinese traditional acupuncture and the other by sham acupuncture. The two groups of acupuncture and placebo treated patients were similar with regard to age, male/female ratio, educational level, and duration and the age of onset of the condition (Table 1). |
| **Deviations from intended interventions** | Some concerns | People delivering the interventions probably aware of their assignment and no appropriate analysis was used. |
| **Missing outcome data** | Low | All participants completed the follow-up. |
| **Measurement of the outcome** | Low | In order to come as close as possible to the double-blind experimental condition, as suggested by Hansen and Hansrn (1983). Each patient was followed by two physicians; the first who administered the treatment had knowledge of the group to which the patient belonged while the second physician, completely ignoring the patient’s treatment, was assigned to collect the diary. |
| **Selection of the reported result** | Some concerns | No pre-registration of the trial protocol |
| **Overall bias** | Some concerns |  |

**White.2000**

| **Bias** | **Authors' judgement** | **Support for judgement** |
| --- | --- | --- |
| **Randomization process** | Low | Centralized random allocation by telephone was performed immediately before the treatment. Randomization lists (block-size of four) were stratied for each centre, prepared from computer-generated random numbers by staff unconnected with the study, and held at a Hospital Pharmacy. No meaningful differences were identified in the prognostic features of the two groups at baseline (Table 1). |
| **Deviations from intended interventions** | Low | The non-adherence to intervention was unrelated to the experimental, and appropriate analysis had been applied. |
| **Missing outcome data** | Low | One subject (sham group) dropped out at the end of the early follow up because of lack of response; one subject (acupuncture group) dropped out at the end of the early follow up as she had started a course of spinal manipulation for her headache. The remaining subjects missed their appointments and did not reply to reminders. |
| **Measurement of the outcome** | Low | The nursing assistant interviewed the subjects in a separate room and remained unaware of their group allocation. Subjects were asked to show headache diaries to the nurse but not to the acupuncturist. |
| **Selection of the reported result** | Some concerns | No pre-registration of the trial protocol |
| **Overall bias** | Some concerns |  |

**Wu.2016**

| **Bias** | **Authors' judgement** | **Support for judgement** |
| --- | --- | --- |
| **Randomisation process** | Some concerns | No conceal of allocation was described. |
| **Deviations from intended interventions** | Some concerns | Blinding the acupuncture practitioner was impossible due to methodological reasons. |
| **Missing outcome data** | Low | All participants completed the follow-up. |
| **Measurement of the outcome** | Some concerns | No blinding of outcome assessment was described. |
| **Selection of the reported result** | Some concerns | No pre-registration of the trial protocol |
| **Overall bias** | Some concerns |  |

**Wang.2018**

| **Bias** | **Authors' judgement** | **Support for judgement** |
| --- | --- | --- |
| **Randomization process** | Some concerns | No conceal of allocation was described. |
| **Deviations from intended interventions** | Some concerns | Blinding the acupuncture practitioner was impossible due to methodological reasons. |
| **Missing outcome data** | Low | All participants completed the follow-up. |
| **Measurement of the outcome** | Some concerns | No blinding of outcome assessment was described. |
| **Selection of the reported result** | Some concerns | No pre-registration of the trial protocol |
| **Overall bias** | Some concerns |  |

**White.1996**

| **Bias** | **Authors' judgement** | **Support for judgement** |
| --- | --- | --- |
| **Randomization process** | Some concerns | No conceal of allocation was described. At the end of the run-in period each subject was randomly allocated to one of two groups by telephoning a secretary who consulted a list derived from computer generated block randomized numbers. After randomization there was no further discussion between doctor and patient about the condition or the treatment, except to indicate tenderness or de qi. |
| **Deviations from intended interventions** | Some concerns | Blinding the acupuncture practitioner was impossible due to methodological reasons and without appropriate analysis. |
| **Missing outcome data** | Low | Almost all participants completed the therapeutic program and were analyzed. |
| **Measurement of the outcome** | Low | Questions, discussion and assessments were handled by the assistant, who remained blinded to the subiect's group allocation. |
| **Selection of the reported result** | Some concerns | No pre-registration of the trial protocol |
| **Overall bias** | Some concerns |  |

**Wang.2022**

| **Bias** | **Authors' judgement** | **Support for judgement** |
| --- | --- | --- |
| **Randomization process** | Some concerns | No conceal of allocation was described. |
| **Deviations from intended interventions** | Some concerns | Blinding the acupuncture practitioner was impossible due to methodological reasons and without appropriate analysis. |
| **Missing outcome data** | Low | All participants completed the therapeutic program and were analyzed. |
| **Measurement of the outcome** | Some concerns | No blinding of outcome assessment was described. |
| **Selection of the reported result** | Some concerns | No pre-registration of the trial protocol |
| **Overall bias** | Some concerns |  |

**Xiang.2015**

| **Bias** | **Authors' judgement** | **Support for judgement** |
| --- | --- | --- |
| **Randomization process** | Some concerns | No conceal of allocation was described and no description of baseline between two groups. |
| **Deviations from intended interventions** | Some concerns | Blinding the acupuncture practitioner was impossible due to methodological reasons and no appropriate analysis was used. |
| **Missing outcome data** | Low | Almost all participants completed the therapeutic program and were analyzed. |
| **Measurement of the outcome** | Some concerns | No blinding of outcome assessment was described. |
| **Selection of the reported result** | Some concerns | No pre-registration of the trial protocol |
| **Overall bias** | Some concerns |  |

**Xue.2004**

| **Bias** | **Authors' judgement** | **Support for judgement** |
| --- | --- | --- |
| **Randomization process** | Low | Each patient was randomly assigned to either group A or group B by drawing an envelope containing a randomization number generated by a computer program. After the participant agreed to participate in the trial and baseline assessments were performed, the opaque envelope was opened by the investigator responsible for the intervention application. There were no statistically significant differences in these parameters between the 2 groups. |
| **Deviations from intended interventions** | Some concerns | Blinding the acupuncture practitioner was impossible due to methodological reasons and without appropriate analysis. |
| **Missing outcome data** | Low | Almost all participants completed the therapeutic program and were analyzed.··· |
| **Measurement of the outcome** | Some concerns | No blinding of outcome assessment was described. |
| **Selection of the reported result** | Low | The protocol for the trial is presented in Figure 1. |
| **Overall bias** | Some concerns |  |

**Yang.2020**

| **Bias** | **Authors' judgement** | **Support for judgement** |
| --- | --- | --- |
| **Randomization process** | Some concerns | No conceal of allocation was described. |
| **Deviations from intended interventions** | Some concerns | Blinding the acupuncture practitioner was impossible due to methodological reasons and without appropriate analysis. |
| **Missing outcome data** | Low | Almost all participants completed the therapeutic program and were analyzed. |
| **Measurement of the outcome** | Some concerns | No blinding of outcome assessment was described. |
| **Selection of the reported result** | Some concerns | No pre-registration of the trial protocol |
| **Overall bias** | Some concerns |  |

**Zhou.2015**

| **Bias** | **Authors' judgement** | **Support for judgement** |
| --- | --- | --- |
| **Randomisation process** | Some concerns | Patients were randomized with a standard table of random numbers. No conceal of allocation was described. |
| **Deviations from intended interventions** | Some concerns | Blinding the acupuncture practitioner was impossible due to methodological reasons and without appropriate analysis. |
| **Missing outcome data** | Low | Almost all participants completed the therapeutic program and were analyzed. |
| **Measurement of the outcome** | Some concerns | No blinding of outcome assessment was described. |
| **Selection of the reported result** | Some concerns | No pre-registration of the trial protocol |
| **Overall bias** | Some concerns |  |

**Zhang.2009**

| **Bias** | **Authors' judgement** | **Support for judgement** |
| --- | --- | --- |
| **Randomization process** | Low | Patients were randomized with a standard table of random numbers. The random number table and assignment are responsible by special personnel. No report about the baseline compare between two groups. |
| **Deviations from intended interventions** | Some concerns | Blinding the acupuncture practitioner was impossible due to methodological reasons and without appropriate analysis. |
| **Missing outcome data** | Low | Almost all participants completed the therapeutic program and were analyzed. |
| **Measurement of the outcome** | Some concerns | No blinding of outcome assessment was described. |
| **Selection of the reported result** | Some concerns | No pre-registration of the trial protocol |
| **Overall bias** | Some concerns |  |

**Zhang.2021**

| **Bias** | **Authors' judgement** | **Support for judgement** |
| --- | --- | --- |
| **Randomization process** | Some concerns | Randomized digital table. No conceal of allocation was described. |
| **Deviations from intended interventions** | Some concerns | Blinding the acupuncture practitioner was impossible due to methodological reasons and without appropriate analysis. |
| **Missing outcome data** | Low | Almost all participants completed the therapeutic program and were analyzed. |
| **Measurement of the outcome** | Some concerns | No blinding of outcome assessment was described. |
| **Selection of the reported result** | Some concerns | No pre-registration of the trial protocol |
| **Overall bias** | Some concerns |  |

**Zhu.2013**

| **Bias** | **Authors' judgement** | **Support for judgement** |
| --- | --- | --- |
| **Randomization process** | Some concerns | No conceal of allocation was described. |
| **Deviations from intended interventions** | Some concerns | Blinding the acupuncture practitioner was impossible due to methodological reasons and without appropriate analysis. |
| **Missing outcome data** | Low | All participants completed the therapeutic program and were analyzed. |
| **Measurement of the outcome** | Some concerns | No blinding of outcome assessment was described. |
| **Selection of the reported result** | Some concerns | No pre-registration of the trial protocol |
| **Overall bias** | Some concerns |  |

**Zheng.2022**

| **Bias** | **Authors' judgement** | **Support for judgement** |
| --- | --- | --- |
| **Randomization process** | Low | Patients were randomized with computer-generated allocation sequence. |
| **Deviations from intended interventions** | Low | The non-adherence to intervention was unrelated to the experimental, and appropriate analysis had been applied. |
| **Missing outcome data** | Low | Almost all patients complete the trial. |
| **Measurement of the outcome** | Low | The outcome assessor and the patient were blinded |
| **Selection of the reported result** | Low | All relevant outcomes were described. |
| **Overall bias** | Low |  |

**6:** **Effects of Acupuncture on TTH**


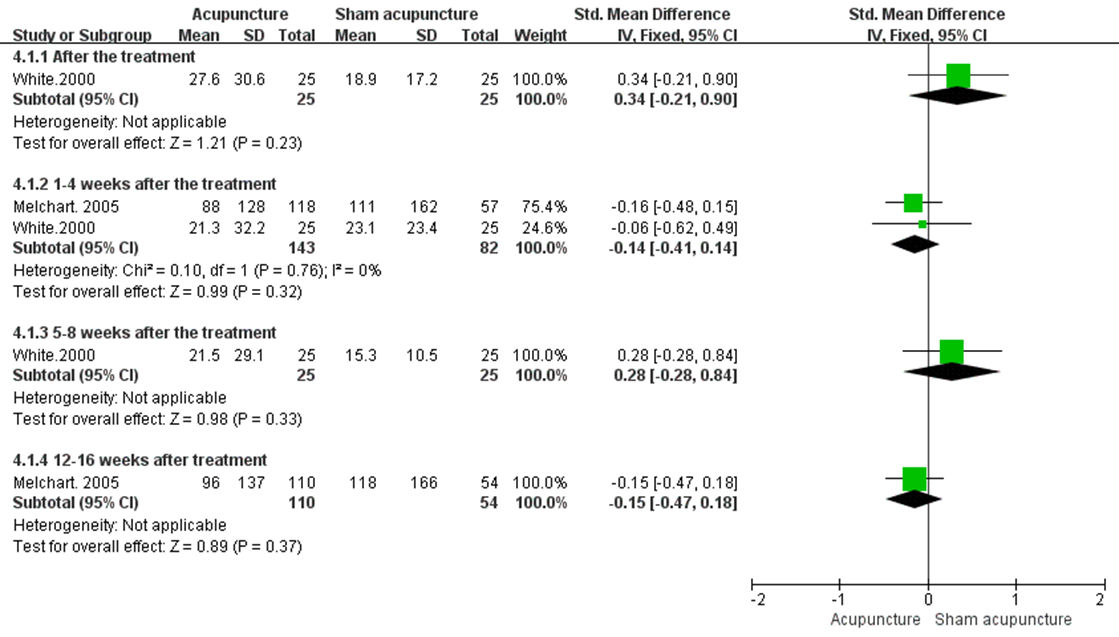


***Figure 1. Headache duration in comparison of acupuncture vs. sham acupuncture***


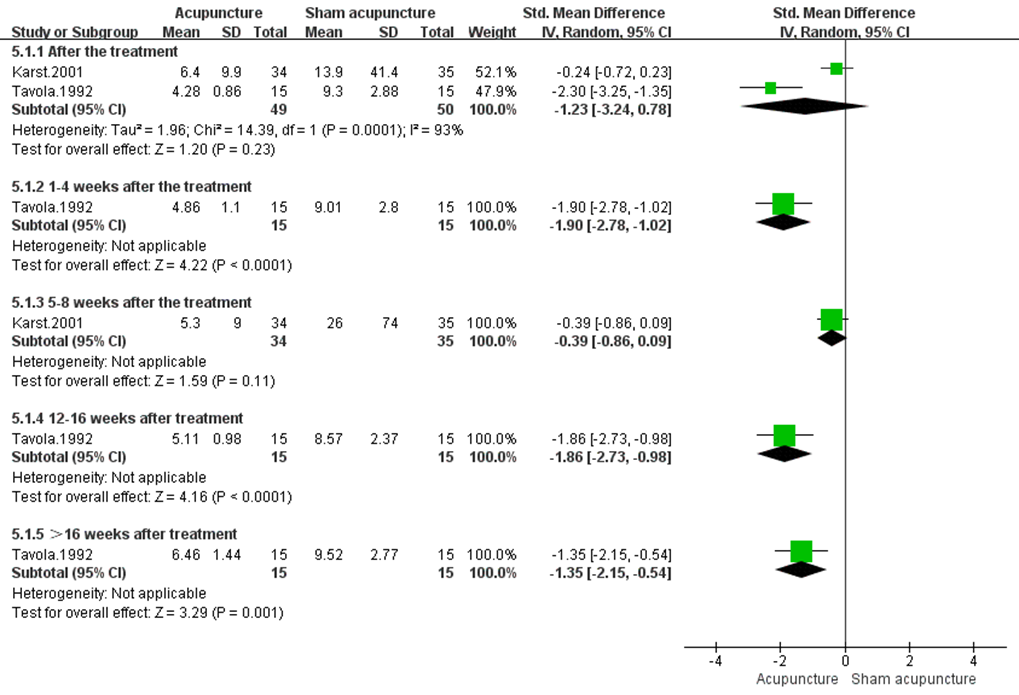


***Figure 2. Consumption of medication in comparison of acupuncture vs. sham acupuncture***


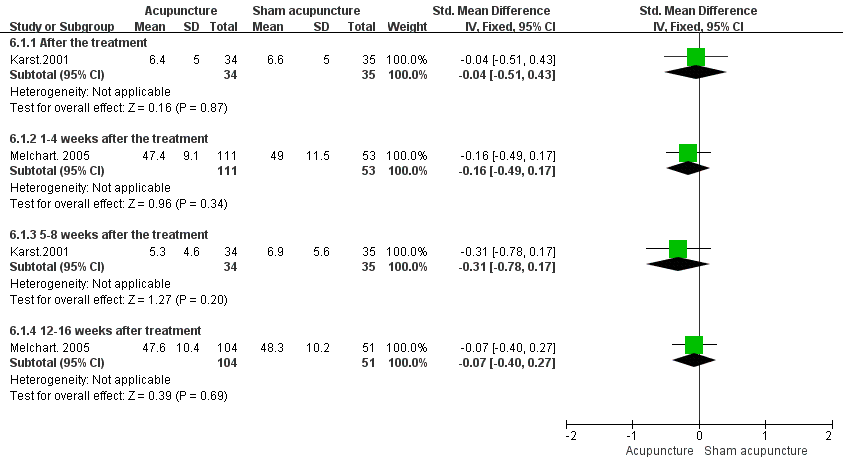


***Figure 3. Depression in comparison of acupuncture vs. sham acupuncture***


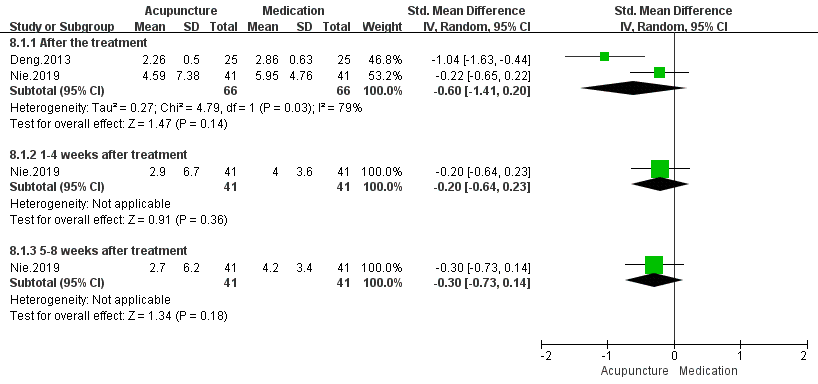


***Figure 4. Headache frequency in comparison of acupuncture vs. medication***


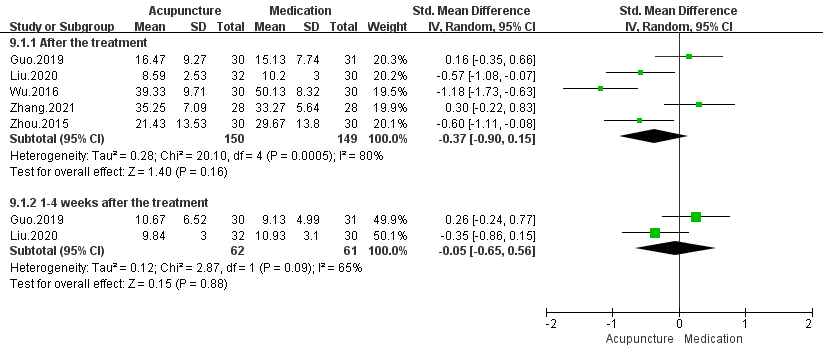


***Figure 5. Depression in comparison of acupuncture vs. medication***


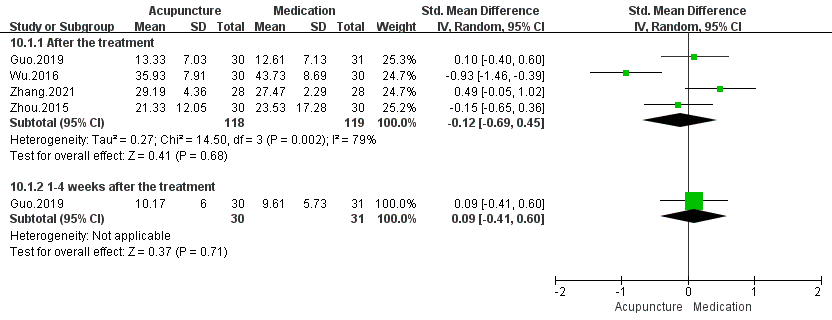


***Figure 6. Anxiety in comparison of acupuncture vs. medication***


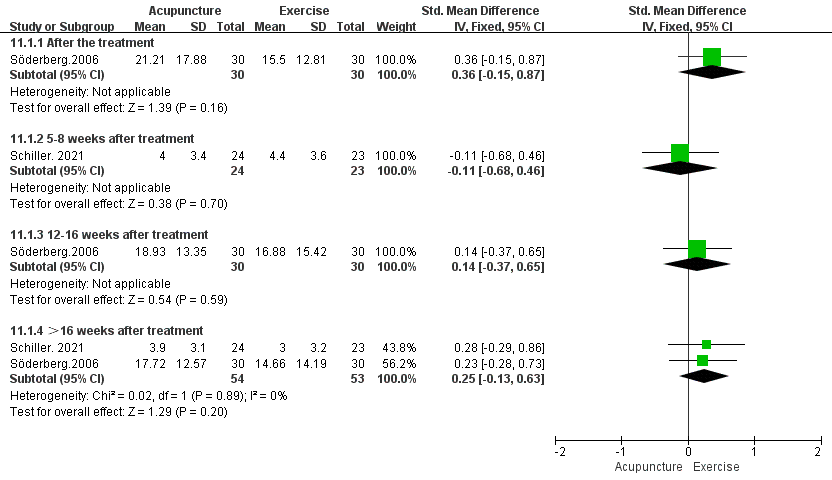


***Figure 7. Pain intensity in comparison of acupuncture vs. exercise***

**7: Subgroup analysis**

***7.1 Responder rate in comparison of acupuncture vs. Sham (Subgroup by frequency of acupuncture)***

***7.2 Headache frequency in comparison of acupuncture vs. Sham (Subgroup by frequency of acupuncture)***

***7.3 Pain intensity in comparison of acupuncture vs. Sham (Subgroup by frequency of acupuncture)***


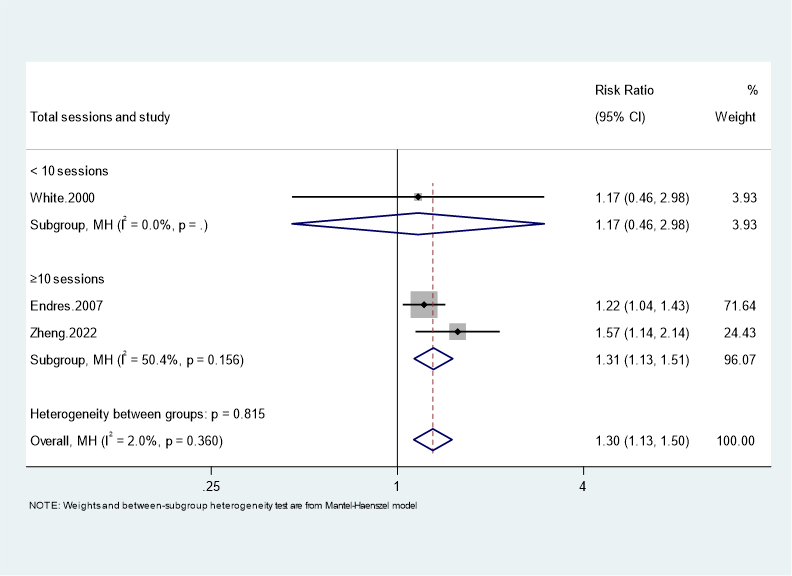


***7.4 Responder rate in comparison of acupuncture vs. Sham (Subgroup by total sessions)***


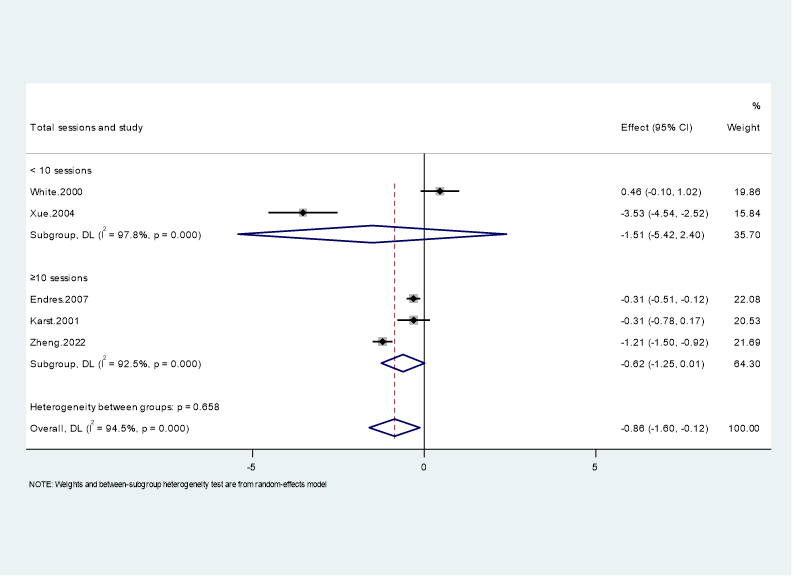


***7.5 Headache frequency in comparison of acupuncture vs. Sham (Subgroup by total sessions)***


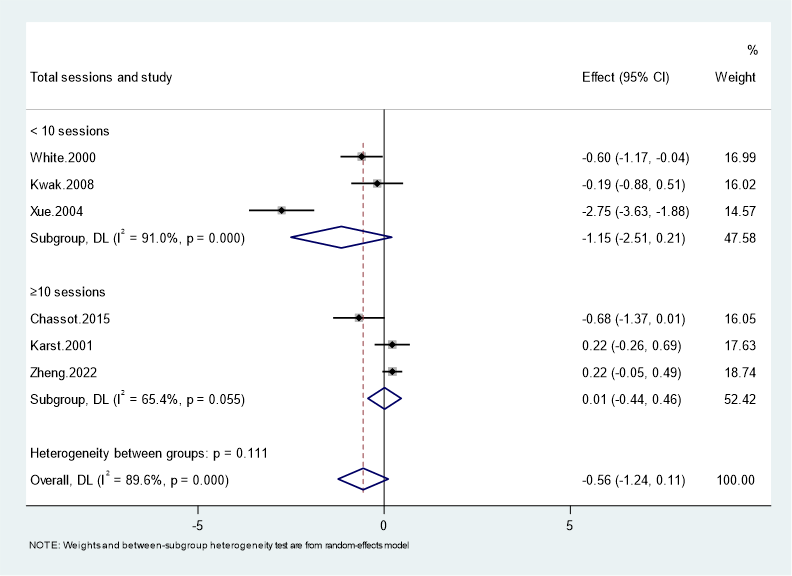


***7.6 Pain intensity in comparison of acupuncture vs. Sham (Subgroup by total sessions)***

***7.7 Headache frequency in comparison of acupuncture vs. Sham (Subgroup by treatment duration)***

***7.8 Pain intensity in comparison of acupuncture vs. Sham (Subgroup by treatment duration)***

***7.9 Responder rate in comparison of acupuncture vs. Sham (Subgroup by needle retention)***

***7.10 Headache frequency in comparison of acupuncture vs. Sham (Subgroup by needle retention)***

***7.11 Pain intensity in comparison of acupuncture vs. Sham (Subgroup by needle retention)***

***7.12 Headache frequency in comparison of acupuncture vs. Sham***

***(Subgroup by type of acupuncture)***

***7.13 Pain intensity in comparison of acupuncture vs. Sham (Subgroup by type of acupuncture)***

***7.14 Pain intensity in comparison of acupuncture vs. medication***

***(Subgroup by categories of medication)***

******

***7.15 Depression in comparison of acupuncture vs. medication***

***(Subgroup by categories of medication)***

******

***7.16 Anxiety in comparison of acupuncture vs. medication (Subgroup by categories of medication)***

**8: Sensitivity analysis**

***8.1.*** ***Responder rate in comparison of acupuncture vs. sham acupuncture (after treatment)***

***8.2. Pain intensity in comparison of acupuncture vs. sham acupuncture (after treatment)***

***8.3. Headache frequency in comparison of acupuncture vs. sham acupuncture (after treatment)***

******

***8.4. Pain intensity in comparison of acupuncture vs. medication***

***8.5. Depression in comparison of acupuncture vs. medication***

***8.6. Anxiety in comparison of acupuncture vs. medication***

**9: Publication bias**

***Responder rate in comparison of acupuncture vs. sham (after treatment)***


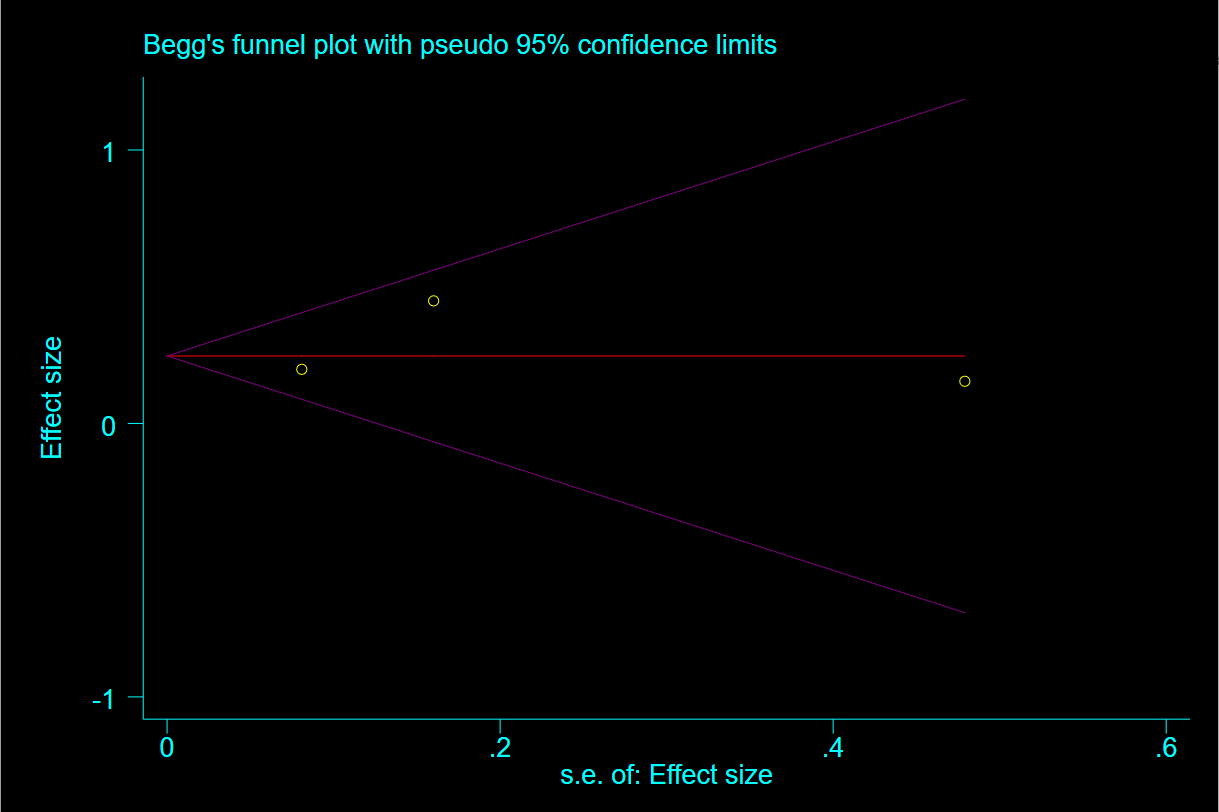


*Begg's* Test: *P*= 1.000


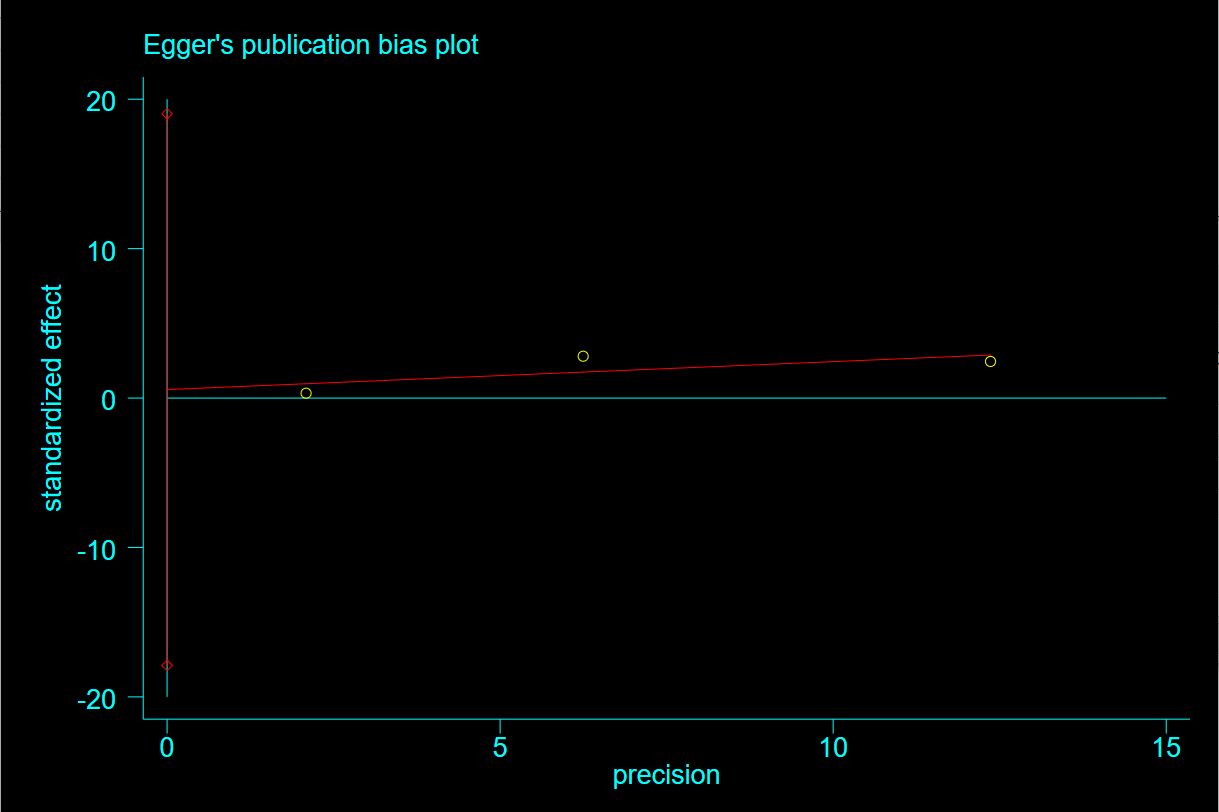


*Egger's* test: *P*= 0.764

**10: Summary of findings table from GRADE profiler**

| **Certainty assessment** | | | | | | | **№ of patient** | | **Effect** | | | | **Certainty** |
| --- | --- | --- | --- | --- | --- | --- | --- | --- | --- | --- | --- | --- | --- |
| ***№* of studies** | **Study**  **design** | **Risk of**  **bias** | **Inconsistency** | **Indirectness** | **Imprecision** | **Publication**  **bias** | **I** | **C** | **Relative**  **(95% CI)** | | **Absolute**  **(95% CI)** | |  |
| *Acupuncture vs Sham acupuncture* | | | | | | | | | | | | | |
| **Responder rate (After treatment)** | | | | | | | | | | | | | |
| 3 | RCT | Not serious | Not serious | Not serious | Serious | Undetected | 339 | 324 | RR 1.30 (1.13 to1.50) | | / | | **⊕⊕⊕**〇 Moderate |
| **Responder rate (1-4 weeks after treatment)** | | | | | | | | | | | | | |
| \| 3 \| RCT \| Not serious \| Not serious \| Not serious \| Serious \| Undetected \| 267 \| 196 \| RR 1.55 (1.25 to1.92) \| / \| **⊕⊕⊕**〇 Moderate \| \| --- \| --- \| --- \| --- \| --- \| --- \| --- \| --- \| --- \| --- \| --- \| --- \| | | | | | | | | | | | | | |
| **Responder rate (5-8 weeks after treatment)** | | | | | | | | | | | | | |
| \| 3 \| RCT \| Not serious \| Not serious \| Not serious \| Serious \| Undetected \| 334 \| 325 \| RR 1.30 (1.13 to1.50) \| / \| **⊕⊕⊕**〇 Moderate \| \| --- \| --- \| --- \| --- \| --- \| --- \| --- \| --- \| --- \| --- \| --- \| --- \| | | | | | | | | | | | | | |
| **Responder rate (12-16 weeks after treatment)** | | | | | | | | | | | | | |
| \| 1 \| RCT \| Not serious \| Not serious \| Not serious \| Very serious \| Undetected \| 110 \| 108 \| RR 1.39 (1.10 to1.75) \| / \| **⊕⊕**〇〇 Low \| \| --- \| --- \| --- \| --- \| --- \| --- \| --- \| --- \| --- \| --- \| --- \| --- \| | | | | | | | | | | | | | |
| **Responder rate (＞16 weeks after treatment)** | | | | | | | | | | | | | |
| \| 2 \| RCT \| Not serious \| Not serious \| Not serious \| Serious \| Undetected \| 314 \| 302 \| RR 1.26 (1.11 to1.44) \| / \| **⊕⊕⊕**〇 Moderate \| \| --- \| --- \| --- \| --- \| --- \| --- \| --- \| --- \| --- \| --- \| --- \| --- \| | | | | | | | | | | | | | |
| \| **Certainty assessment** \| \| \| \| \| \| \| **№ of patient** \| \| **Effect** \| \| **Certainty** \| \| --- \| --- \| --- \| --- \| --- \| --- \| --- \| --- \| --- \| --- \| --- \| --- \| \| ***№* of studies** \| **Study**  **design** \| **Risk of**  **bias** \| **Inconsistency** \| **Indirectness** \| **Imprecision** \| **Publication**  **bias** \| **I** \| **C** \| **Relative**  **(95% CI)** \| **Absolute**  **(95% CI)** \| \| **Headache frequency (After treatment)** \| \| \| \| \| \| \| \| \| \| \| \| \| \| 5 \| RCT \| Serious \| Very serious \| Not serious \| Not serious \| Undetected \| 393 \| 379 \| / \| SMD −0.85 (−1.58, −0.12) **⊕**〇〇〇 Very low \| \| --- \| --- \| --- \| --- \| --- \| --- \| --- \| --- \| --- \| --- \| --- \| \| \| \| \| \| \| \| \| \| \| \| \| \| **Headache frequency (1-4 weeks after treatment)** \| \| \| \| \| \| \| \| \| \| \| \| \| \| 4 \| RCT \| Serious \| Very serious \| Not serious \| Serious \| Undetected \| 268 \| 205 \| / \| SMD −0.59 (−1.48, 0.30) **⊕**〇〇〇 Very low \| \| --- \| --- \| --- \| --- \| --- \| --- \| --- \| --- \| --- \| --- \| --- \| \| \| \| \| \| \| \| \| \| \| \| \| \| **Headache frequency (5-8 weeks after treatment)** \| \| \| \| \| \| \| \| \| \| \| \| \| \| 4 \| RCT \| Serious \| Very serious \| Not serious \| Serious \| Undetected \| 368 \| 360 \| / \| SMD −0.44 (−1.18, 0.30) \| **⊕**〇〇〇 Very low \| \| --- \| --- \| --- \| --- \| --- \| --- \| --- \| --- \| --- \| --- \| --- \| --- \| \| \| \| \| \| \| \| \| \| \| \| \| \| **Headache frequency (12-16 weeks after treatment)** \| \| \| \| \| \| \| \| \| \| \| \| \| \| \| 2 \| RCT \| Not serious \| Very serious \| Not serious \| Very serious \| Undetected \| 222 \| 163 \| / \| SMD −0.84 (−2.31, 0.62) \| **⊕**〇〇〇 Very low \| \| --- \| --- \| --- \| --- \| --- \| --- \| --- \| --- \| --- \| --- \| --- \| --- \| \| \| --- \| --- \| --- \| --- \| --- \| --- \| --- \| --- \| --- \| --- \| --- \| --- \| --- \| \| \| \| \| \| \| \| \| \| \| \| \| \| **Headache frequency (＞16 weeks after treatment)** \| \| \| \| \| \| \| \| \| \| \| \| \| \| 3 \| RCT \| Not serious \| Very serious \| Not serious \| Serious \| Undetected \| 348 \| 337 \| / \| SMD −0.63 (−1.48, 0.21) \| **⊕**〇〇〇 Very low \| \| --- \| --- \| --- \| --- \| --- \| --- \| --- \| --- \| --- \| --- \| --- \| --- \| \| \| \| \| \| \| \| \| \| \| \| \|   **Pain intensity (After treatment)**   \| \| 6 \| RCT \| Serious \| Very serious \| Not serious \| Serious \| Undetected \| 223 \| 203 \| / \| SMD −0.55 (−1.21, 0.11) \| **⊕**〇〇〇 Very low \| \| --- \| --- \| --- \| --- \| --- \| --- \| --- \| --- \| --- \| --- \| --- \| --- \| \| \| --- \| --- \| --- \| --- \| --- \| --- \| --- \| --- \| --- \| --- \| --- \| --- \| --- \|  \| **Certainty assessment** \| \| \| \| \| \| \| ***№* of patient** \| \| **Effect** \| \| **Certainty** \| \| --- \| --- \| --- \| --- \| --- \| --- \| --- \| --- \| --- \| --- \| --- \| --- \| \| ***№* of studies** \| **Study**  **design** \| **Risk of**  **Bias** \| **Inconsistency** \| **Indirectness** \| **Imprecision** \| **Publication bias** \| **I** \| **C** \| **Relative**  **(95% CI)** \| **Absolute**  **(95% CI)** \| | | | | | | | | | | | | | |
| **Pain intensity (1-4 weeks after treatment)** | | | | | | | | | | | | | |
| \| \| 6 \| RCT \| Serious \| Very serious \| Not serious \| Serious \| Undetected \| 326 \| 262 \| / \| SMD −0.42 (−0.93, 0.09) \| **⊕**〇〇〇 Very low \| \| --- \| --- \| --- \| --- \| --- \| --- \| --- \| --- \| --- \| --- \| --- \| --- \| \| \| --- \| --- \| --- \| --- \| --- \| --- \| --- \| --- \| --- \| --- \| --- \| --- \| --- \| | | | | | | | | | | | | | |
| **Pain intensity (5-8 weeks after treatment)** | | | | | | | | | | | | | |
| \| \| 5 \| RCT \| Serious \| Not Serious \| Not serious \| Not serious \| Undetected \| 384 \| 374 \| / \| SMD −0.17 (−0.32, −0.01) \| **⊕⊕⊕**〇 Moderate \| \| --- \| --- \| --- \| --- \| --- \| --- \| --- \| --- \| --- \| --- \| --- \| --- \| \| \| --- \| --- \| --- \| --- \| --- \| --- \| --- \| --- \| --- \| --- \| --- \| --- \| --- \| | | | | | | | | | | | | | |
| **Pain intensity (12-16 weeks after treatment)** | | | | | | | | | | | | | |
| \| \| 4 \| RCT \| Serious \| Serious \| Not serious \| Not serious \| Undetected \| 280 \| 218 \| / \| SMD −0.35 (−0.70, −0.01) \| **⊕⊕**〇〇 Low \| \| --- \| --- \| --- \| --- \| --- \| --- \| --- \| --- \| --- \| --- \| --- \| --- \| \| \| --- \| --- \| --- \| --- \| --- \| --- \| --- \| --- \| --- \| --- \| --- \| --- \| --- \| | | | | | | | | | | | | | |
| **Pain intensity (＞16 weeks after treatment)** | | | | | | | | | | | | | |
| \| \| 3 \| RCT \| Not serious \| Not serious \| Not serious \| Not serious \| Undetected \| 348 \| 337 \| / \| SMD −0.26 (−0.44, −0.07) \| **⊕⊕⊕⊕** High \| \| --- \| --- \| --- \| --- \| --- \| --- \| --- \| --- \| --- \| --- \| --- \| --- \| \| \| --- \| --- \| --- \| --- \| --- \| --- \| --- \| --- \| --- \| --- \| --- \| --- \| --- \| | | | | | | | | | | | | | |
| **Headache duration (After treatment)** | | | | | | | | | | | | | |
| \| 1 \| RCT \| Serious \| Not serious \| Not serious \| Very serious \| Undetected \| 25 \| 25 \| / \| SMD 0.34 (−0.21, 0.90) **⊕**〇〇〇 Very low \| \| --- \| --- \| --- \| --- \| --- \| --- \| --- \| --- \| --- \| --- \| --- \| | | | | | | | | | | | | | |
| **Headache duration (1-4 weeks after treatment)** | | | | | | | | | | | | | |
| \| 2 \| RCT \| Serious \| Not serious \| Not serious \| Very serious \| Undetected \| 143 \| 82 \| / \| SMD −0.14 (−0.41, 0.14) **⊕**〇〇〇 Very low \| \| --- \| --- \| --- \| --- \| --- \| --- \| --- \| --- \| --- \| --- \| --- \| | | | | | | | | | | | | | |
| \| \| **Certainty assessment** \| \| \| \| \| \| \| ***№* of patient** \| \| **Effect** \| \| **Certainty** \| \| --- \| --- \| --- \| --- \| --- \| --- \| --- \| --- \| --- \| --- \| --- \| --- \| \| ***№* of studies** \| **Study**  **design** \| **Risk of**  **Bias** \| **Inconsistency** \| **Indirectness** \| **Imprecision** \| **Publication bias** \| **I** \| **C** \| **Relative**  **(95% CI)** \| **Absolute**  **(95% CI)** \| \| \| --- \| --- \| --- \| --- \| --- \| --- \| --- \| --- \| --- \| --- \| --- \| --- \| --- \| --- \| --- \| --- \| --- \| --- \| --- \| --- \| --- \| --- \| --- \| --- \|   **Headache duration (5-8 weeks after treatment)**   \| \| 1 \| RCT \| Serious \| Not serious \| Not serious \| Very serious \| Undetected \| 25 \| 25 \| / \| SMD 0.28 (−0.28, 0.84) **⊕**〇〇〇 Very low \| \| --- \| --- \| --- \| --- \| --- \| --- \| --- \| --- \| --- \| --- \| --- \| \| \| --- \| --- \| --- \| --- \| --- \| --- \| --- \| --- \| --- \| --- \| --- \| --- \|   **Headache duration (12-16 weeks after treatment)**   \| 1 \| RCT \| Not serious \| Not serious \| Not serious \| Very serious \| Undetected \| 110 \| 54 \| / \| SMD −0.15 (−0.47, 0.18) **⊕⊕**〇〇 Low \| \| --- \| --- \| --- \| --- \| --- \| --- \| --- \| --- \| --- \| --- \| --- \| | | | | | | | | | | | | | |
| **Consumption of medication (After treatment)** | | | | | | | | | | | | | |
| \| 2 \| RCT \| Serious \| Very serious \| Not serious \| Very serious \| Undetected \| 49 \| 50 \| / \| SMD −1.23 (−3.24, 0.78) \| **⊕**〇〇〇Very low \| \| --- \| --- \| --- \| --- \| --- \| --- \| --- \| --- \| --- \| --- \| --- \| --- \| | | | | | | | | | | | | | |
| **Consumption of medication (1-4 weeks after treatment)** | | | | | | | | | | | | | |
| \| 1 \| RCT \| Serious \| Not serious \| Not serious \| Serious \| Undetected \| 15 \| 15 \| / \| SMD −1.90 (−2.78, −1.02) \| **⊕⊕**〇〇 Low \| \| --- \| --- \| --- \| --- \| --- \| --- \| --- \| --- \| --- \| --- \| --- \| --- \| | | | | | | | | | | | | | |
| **Consumption of medication (5-8 weeks after treatment)** | | | | | | | | | | | | | |
| \| 1 \| RCT \| Serious \| Not serious \| Not serious \| Very serious \| Undetected \| 34 \| 35 \| / \| SMD −0.39 (−0.86, 0.09) \| **⊕**〇〇〇Very low \| \| --- \| --- \| --- \| --- \| --- \| --- \| --- \| --- \| --- \| --- \| --- \| --- \| | | | | | | | | | | | | | |
| **Consumption of medication (12-16 weeks after treatment)**   \| 1 \| RCT \| Serious \| Not serious \| Not serious \| Serious \| Undetected \| 15 \| 15 \| / \| SMD −1.86 (−2.73, −0.98) \| **⊕⊕**〇〇 Low \| \| --- \| --- \| --- \| --- \| --- \| --- \| --- \| --- \| --- \| --- \| --- \| --- \| | | | | | | | | | | | | | |
|  | | | | | | | | | | | | | |
| \| **Certainty assessment** \| \| \| \| \| \| \| ***№* of patient** \| \| **Effect** \| \| **Certainty** \| \| --- \| --- \| --- \| --- \| --- \| --- \| --- \| --- \| --- \| --- \| --- \| --- \| \| ***№* of studies** \| **Study**  **design** \| **Risk of**  **Bias** \| **Inconsistency** \| **Indirectness** \| **Imprecision** \| **Publication bias** \| **I** \| **C** \| **Relative**  **(95% CI)** \| **Absolute**  **(95% CI)** \|   **Consumption of medication (＞16 weeks after treatment)**   \| 1 \| RCT \| Serious \| Not serious \| Not serious \| Serious \| Undetected \| 15 \| 15 \| / \| SMD −1.35 (−2.15, −0.54) \| **⊕⊕**〇〇 Low \| \| --- \| --- \| --- \| --- \| --- \| --- \| --- \| --- \| --- \| --- \| --- \| --- \|   **Depression (After treatment)** | | | | | | | | | | | | | |
| 1 | RCT | Serious | Not serious | Not serious | Very serious | Undetected | 34 | 35 | / | SMD −0.04 (−0.51, 0.43) | | **⊕**〇〇〇Very low | |
| **Depression (1-4 weeks after treatment)** | | | | | | | | | | | | | |
| 1 | RCT | Not serious | Not serious | Not serious | Very serious | Undetected | 111 | 53 | / | SMD −0.16 (−0.49, 0.17) | | **⊕⊕**〇〇 Low | |
| **Depression (5-8 weeks after treatment)** | | | | | | | | | | | | | |
| 1 | RCT | Serious | Not serious | Not serious | Very serious | Undetected | 34 | 35 | / | SMD −0.31 (−0.78, 0.17) | | **⊕**〇〇〇Very low | |
| **Depression (12-16 weeks after treatment)** | | | | | | | | | | | | | |
| 1 | RCT | Not serious | Not serious | Not serious | Very serious | Undetected | 104 | 51 | / | SMD −0.07 (−0.40, 0.27) | | **⊕⊕**〇〇 Low | |
| *Acupuncture vs* *Medication* | | | | | | | | | | | | | |
| **Pain intensity (After treatment)** | | | | | | | | | | | | | |
| 9 | RCT | Serious | Serious | Not serious | Not serious | Undetected | 441 | 390 | / | SMD −0.62 (−0.86, −0.38) | | **⊕⊕**〇〇 Low | |
| \| **Certainty assessment** \| \| \| \| \| \| \| ***№* of patient** \| \| **Effect** \| \| **Certainty** \| \| --- \| --- \| --- \| --- \| --- \| --- \| --- \| --- \| --- \| --- \| --- \| --- \| \| ***№* of studies** \| **Study**  **design** \| **Risk of**  **Bias** \| **Inconsistency** \| **Indirectness** \| **Imprecision** \| **Publication bias** \| **I** \| **C** \| **Relative**  **(95% CI)** \| **Absolute**  **(95% CI)** \|   **Pain intensity (1-4 weeks after treatment)** | | | | | | | | | | | | | |
| 2 | RCT | Serious | Very serious | Not serious | Serious | Undetected | 81 | 89 | / | SMD −1.35 (−2.49, −0.21) | | **⊕**〇〇〇Very low | |
| **Pain intensity (5-8 weeks after treatment)** | | | | | | | | | | | | | |
| 1 | RCT | Serious | Not serious | Not serious | Serious | Undetected | 41 | 41 | / | SMD −1.03 (−1.49, −0.57) | | **⊕⊕**〇〇 Low | |
| **Pain intensity (12-16 weeks after treatment)** | | | | | | | | | | | | | |
| 1 | RCT | Serious | Not serious | Not serious | Serious | Undetected | 40 | 48 | / | SMD −0.94 (−1.39, −0.50) | | **⊕⊕**〇〇 Low | |
| **Headache frequency (After treatment)** | | | | | | | | | | | | | |
| 2 | RCT | Serious | Very serious | Not serious | Very serious | Undetected | 66 | 66 | / | SMD −0.60 (−1.41, 0.20) | | **⊕**〇〇〇 Very low | |
| **Headache frequency (1-4 weeks after treatment)** | | | | | | | | | | | | | |
| 1 | RCT | Serious | Not serious | Not serious | Very serious | Undetected | 41 | 41 | / | SMD −0.20 (−0.64, 0.23) | | **⊕**〇〇〇 Very low | |
| **Headache frequency (5-8 weeks after treatment)** | | | | | | | | | | | | | |
| 1 | RCT | Serious | Not serious | Not serious | Very serious | Undetected | 41 | 41 | / | SMD −0.30 (−0.73, 0.14) | | **⊕**〇〇〇 Very low | |
| **Depression (After treatment)** | | | | | | | | | | | | | |
| 5 | RCT | Serious | Very serious | Not serious | Very serious | Undetected | 150 | 149 | / | SMD −0.37 (−0.90, 0.15) | | **⊕**〇〇〇 Very low | |
| \| **Certainty assessment** \| \| \| \| \| \| \| ***№* of patient** \| \| **Effect** \| \| **Certainty** \| \| --- \| --- \| --- \| --- \| --- \| --- \| --- \| --- \| --- \| --- \| --- \| --- \| \| ***№* of studies** \| **Study**  **design** \| **Risk of**  **Bias** \| **Inconsistency** \| **Indirectness** \| **Imprecision** \| **Publication bias** \| **I** \| **C** \| **Relative**  **(95% CI)** \| **Absolute**  **(95% CI)** \|   **Depression (1-4 weeks after treatment)** | | | | | | | | | | | | | |
| 2 | RCT | Serious | Serious | Not serious | Very serious | Undetected | 62 | 61 | / | SMD −0.05 (−0.65, 0.56) | | **⊕**〇〇〇 Very low | |
| **Anxiety (After treatment)** | | | | | | | | | | | |  | |
| 4 | RCT | Serious | Very serious | Not serious | Very serious | Undetected | 118 | 119 | / | SMD −0.12 (−0.69, 0.45) | | **⊕**〇〇〇 Very low | |
| **Anxiety (1-4 weeks after treatment)** | | | | | | | | | | | | | |
| 1 | RCT | Serious | Not serious | Not serious | Very serious | Undetected | 30 | 31 | / | SMD 0.09 (−0.41, 0.60) | | **⊕**〇〇〇 Very low | |
| *Acupuncture vs* *Exercise* | | | | | | | | | | | | | |
| **Pain intensity (After treatment)** | | | | | | | | | | | | | |
| \| 1 \| RCT \| Serious \| Not serious \| Not serious \| Very serious \| Undetected \| 30 \| 30 \| / \| SMD 0.36 (−0.15, 0.87) \| **⊕**〇〇〇 Very low \| \| --- \| --- \| --- \| --- \| --- \| --- \| --- \| --- \| --- \| --- \| --- \| --- \| | | | | | | | | | | | | | |
| **Pain intensity (5-8 weeks after treatment)** | | | | | | | | | | | | | |
| \| 1 \| RCT \| Serious \| Not serious \| Not serious \| Very serious \| Undetected \| 24 \| 23 \| / \| SMD −0.11 (−0.68, 0.46) \| **⊕**〇〇〇 Very low \| \| --- \| --- \| --- \| --- \| --- \| --- \| --- \| --- \| --- \| --- \| --- \| --- \| \| **Pain intensity (12-16 weeks after treatment)** \| \| \| \| \| \| \| \| \| \| \| \| \| 1 \| RCT \| Serious \| Not serious \| Not serious \| Very serious \| Undetected \| 30 \| 30 \| / \| SMD 0.14 (−0.37, 0.65) \| **⊕**〇〇〇 Very low \| \| **Pain intensity (＞16 weeks after treatment)** \| \| \| \| \| \| \| \| \| \| \| \| \| 2 \| RCT \| Serious \| Not serious \| Not serious \| Very serious \| Undetected \| 54 \| 53 \| / \| SMD 0.25 (−0.13, 0.63) \| **⊕**〇〇〇 Very low \| | | | | | | | | | | | | | |
| Abbreviation: CI, confidence interval; SMD, standardized mean difference; RR, risk ratio | | | | | | | | | | | |  | |
